# Reversible Light-Induced Dimerization of Secondary Face Azobenzene-Functionalized β-Cyclodextrin Derivatives

**DOI:** 10.1021/acs.joc.3c00564

**Published:** 2023-06-21

**Authors:** Gonzalo Rivero-Barbarroja, Carlos Fernández-Clavero, Cristina García-Iriepa, Gema Marcelo, M. Carmen Padilla-Pérez, Tania Neva, Juan M. Benito, Stéphane Maisonneuve, Carmen Ortiz Mellet, Juan Xie, José M. García Fernández, Francisco Mendicuti

**Affiliations:** † Department of Organic Chemistry, Faculty of Chemistry, University of Seville, Sevilla 41012, Spain; ‡ Departamento de Química Analítica, Química Física e Ingeniería Química and Instituto de Investigación Química “Andrés del Rio”, Universidad de Alcalá, Alcalá de Henares, Madrid 28805, Spain; § Instituto de Investigaciones Químicas (IIQ), CSIC − Universidad de Sevilla, Américo Vespucio 49, 41092 Sevilla, Spain; ∥ ENS Paris-Saclay, CNRS, Photophysique et Photochimie Supramoléculaires et Macromoléculaires, Université Paris-Saclay, Gif-sur-Yvette 91190, France

## Abstract

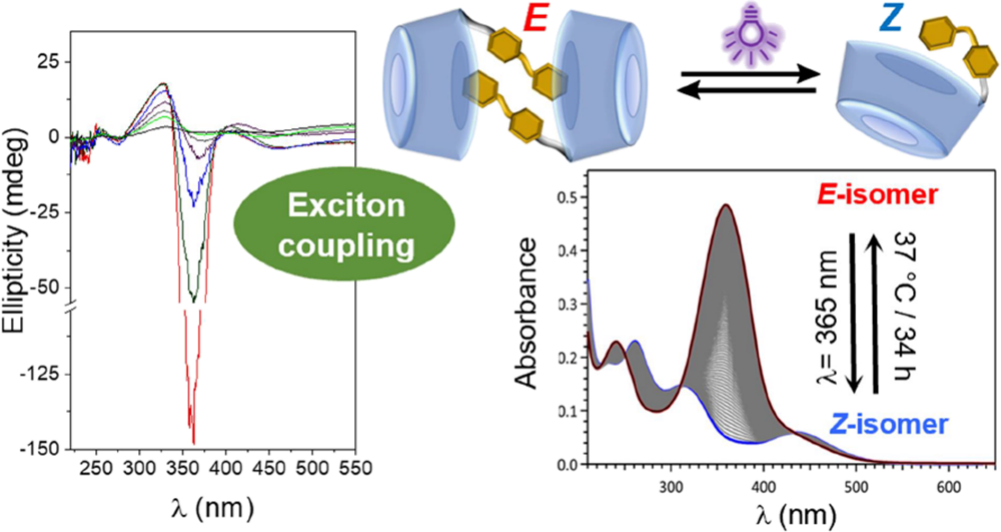

β-cyclodextrin (βCyD) derivatives equipped with aromatic appendages at the secondary face exhibit tailorable self-assembling capabilities. The aromatic modules can participate in inclusion phenomena and/or aromatic–aromatic interactions. Supramolecular species can thus form that, at their turn, can engage in further co-assembling with third components in a highly regulated manner; the design of nonviral gene delivery systems is an illustrative example. Endowing such systems with stimuli responsiveness while keeping diastereomeric purity and a low synthetic effort is a highly wanted advancement. Here, we show that an azobenzene moiety can be “clicked” to a single secondary O-2 position of βCyD affording 1,2,3-triazole-linked βCyD-azobenzene derivatives that undergo reversible light-controlled self-organization into dimers where the monomer components face their secondary rims. Their photoswitching and supramolecular properties have been thoroughly characterized by UV–vis absorption, induced circular dichroism, nuclear magnetic resonance, and computational techniques. As model processes, the formation of inclusion complexes between a water-soluble triazolylazobenzene derivative and βCyD as well as the assembly of native βCyD/βCyD-azobenzene derivative heterodimers have been investigated in parallel. The stability of the host–guest supramolecules has been challenged against the competitor guest adamantylamine and the decrease of the medium polarity using methanol–water mixtures. The collective data support that the *E*-configured βCyD-azobenzene derivatives, in aqueous solution, form dimers stabilized by the interplay of aromatic–aromatic and aromatic-βCyD cavity interactions after partial reciprocal inclusion. Photoswitching to the *Z*-isomer disrupts the dimers into monomeric species, offering opportunity for the spatiotemporal control of the organizational status by light.

## Introduction

The ability to program the reversible assemblage of discrete molecules into well-defined supramolecular edifices is both fundamentally fascinating and central for applications ranging from adjustable data storage/decoding to controlled drug delivery.^1^ Cyclooligosacharides, among which cyclodextrins (CyDs) are iconic examples, are stellar players in the field.^2^ In addition to their intrinsically biocompatible nature, they provide distinct architectures exposing various sets of hydroxyl groups that are addressable through precision chemistry strategies.^3^ Functional elements for specific biomolecular recognition, self-assembly, or stimuli responsiveness can then be installed with predictable spatial orientation while keeping full structural control from the atomic to the nanometric level,^4^ thus fulfilling the notion of “molecular nanoparticles.”^5^ Their cage-like character further enables host–guest approaches that can be implemented in the design of advanced supramolecular materials.^6^

The incorporation of an aromatic module onto a CyD platform has proven particularly useful to preorganize the system for a next supramolecular event.^7^ For example, secondary face aromatic-CyD derivatives can undergo dimer formation in a highly regulated manner. The monomer components in the supramolecular species face their secondary rims with no or only partial inclusion of the aromatic moiety in the cavity of the neighbor CyD partner; they are so-called face-to-face contact dimers.^8^ We have previously used this strategy to access hierarchically assembled nanocomplexes as artificial viruses for nucleic acid delivery.^9^ Most interestingly, the stability of the dimer entity can be made sensitive to the milieu, which offers an appealing way to predetermine nanocomplex fate and cargo release as a function of the environment. Using an azobenzene appendage as the dimerization-promoting aromatic component might add light-responsive capabilities on top, broadening the possibilities for spatiotemporal control of the assembling/dissociation equilibria. On the one hand, azobenzene can reversibly switch between the *E-* and *Z*-isomers upon irradiation at the appropriate wavelength, which has a strong impact in the molecular topology and is expected to affect self-organization processes.^10^ On the other hand, the extended *E*-isomer fits the cavity of β-cyclodextrin (βCyD) and readily forms the corresponding 1:1 inclusion complex, whereas the twisted *Z* diastereomer generally^11^ (although not always)^12^ exhibits a significantly lower association constant. The later property has been broadly exploited to engineer multi(macro)molecular host–guest light-responsive materials.^13^ Surprisingly, reports on cyclodextrin-azobenzene conjugates are rather scarce and supramolecular studies are essentially limited to self- versus intermolecular inclusion complex formation in primary face-linked derivatives.^14^ Regarding secondary face-appended representatives, Jog and Gin^15^ prepared an *O*-2-linked βCyD-azobenzene derivative with a flexible butyl tether in 7.6% yield by direct alkylation of commercial βCyD. The authors showed that this compound forms the expected self-inclusion complex in the *E*-form but not in the *Z*-form, behaving as a gated ion channel. Casas-Solvas and Vargas-Berenguel^16^ reported the synthesis of a similar *O*-2-linked adduct with a rigid alkyne connector between the βCyD and the azobenzene moiety through a Sonogashira-type reaction in 64% yield. To the far of our knowledge, the supramolecular properties were not further investigated. Pursuing our ongoing efforts on input-responsive cyclooligosaccharides with self-assembling behaviors,^17,18^ here, we show that the azobenzene motif can be efficiently installed at the *O*-2 position of βCyD derivatives through copper(I) catalyzed azide-alkyne coupling (CuAAC), the archetypical “click”-type reaction.^19^ The ability of the resulting adducts to reversibly form supramolecular dimers depending on the configurational status of the azo-group, which can be exchanged by light irradiation, has been confirmed by a range of spectroscopic and computational techniques.

## Results and Discussion

### Design Criteria and Synthesis

Preliminary molecular modeling showed that azobenzene moieties appended at the secondary face of βCyD through a rigid 1,4-disubstituted 1,2,3-triazole ring would be unable to undergo significant self-inclusion complex formation in either the *E-* or *Z*-isomer. Indeed, molecular mechanics (MM) calculations showed that conformations locating the azobenzene module, or part of it, inside the βCyD cavity suffer from considerable molecular strain. They rapidly evolved during molecular dynamics (MD) simulations to out-of-the-cavity conformers (data not shown). The rather short triazole linker would also hinder the formation of reciprocal face-to-face dimers with deep reciprocal inclusion of the azobenzene modules due to steric repulsions between the interplaying βCyD macrorings. A thorough description of the computational experiments is provided later on in the Molecular Mechanics and Molecular Dynamics Calculations section (see also the SI for experimental details). We hypothesized that the formation of contact dimers, similar to what has been observed for βCyD-aromatic derivatives with reduced flexibility, would be favored. In this case, there would be either no penetration or only partial penetration of the outermost phenyl portion into the cavity of the co-associating βCyD partner. This scenario has previously proven advantageous to impart reversibility in hierarchical self-assembling schemes.^9^ We also considered the fact that the presence of triazolyl and alkoxy substituents on the azobenzene module ensures a slow equilibration rate between the *E* and *Z*-isomers under thermal conditions. This allows us to assess the impact of light-induced isomerization on the supramolecular properties.^20^ To test these ideas, a systematic assessment of the photochemistry of the triazolylazobenzene system, the consequences of *E*/*Z* configurational switching in the abilities to interact with βCyD, and the impact of “click” conjugation in such processes were mandatory. A tailored three-level working strategy was devised, comprising (i) studying the photochromic behavior of the model triazolyl-armed azobenzene derivative **1**, intentionally equipped with a terminal amine functionality (as the corresponding hydrochloride salt) to warrant water solubility, in comparison with the triazole-connected βCyD-azobenzene compounds **2** and **3**; (ii) investigating the thermodynamic and structural features of supramolecular complex formation between **1** or **3**, each in the two possible isomeric forms, and native βCyD; and (iii) examining the propensity of compounds **2** and **3** to undergo supramolecular dimerization, analyzing the structure of the resulting species and probing the effect of photoisomerization in the corresponding self-assembling equilibria.

**Scheme 1:**
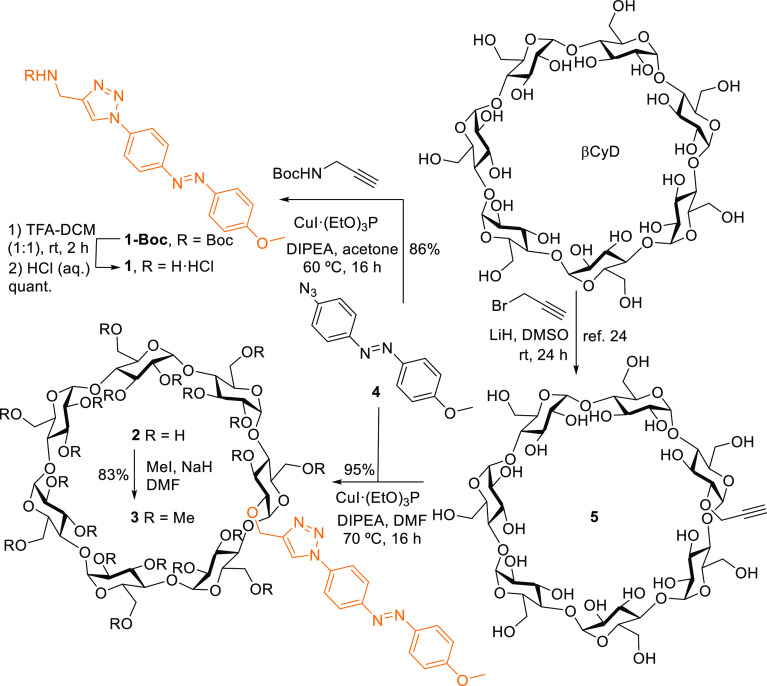
Structures and Synthesis of the Triazolylazobenzene Model Compound **1** and the Triazole-Linked βCyD-Azobenzene Derivatives **2** and **3**

The triazolyl-equipped control azobenzene derivative **1**, devoid from the βCyD component, was assembled by CuAAC of 4-azido-4′-methoxyazobezene **4** and propargylamine in acetone, using copper(I) iodide-triethylphosphite [CuI·(EtO)_3_P] as the Cu(I) source (81% yield).^21^ Precursor **4**, previously obtained over five steps by diazo formation on solid support,^22^ was readily accessed by methylation of the phenol group in 4-azido-4′-hydroxyazobezene (98% yield).^23^ The same CuAAC procedure starting from **4** and the known (2-*O*)-monopropargylated βCyD derivative **5**, accessible in a single step from commercial βCyD,^24^ in DMF at 70 °C afforded compound **2** in 95% yield. Finally, conventional methylation of **2** with methyl iodide and sodium hydride provided the corresponding eicosa-*O*-methyl-βCyD-azobenzene derivative **3** (83% yield; Scheme 1 and the Supporting Information, Figures S1–S4).

The decision to include the methylated derivative **3**, alongside the hydroxylated conjugate **2**, in this study, was based on previous literature findings, suggesting that the increased secondary rim diameter ratio and enhanced cavity flexibility in permethylated βCyD, compared to native βCyD, can confer induced-fitting and discrimination abilities toward azobenzene guests.^25^ We found, however, virtually identical behaviors for both compounds in all the experimental settings used in this work. To avoid unnecessary duplication, representative data for either **2** or **3** will be presented; unless otherwise stated, they are transposable.

### UV–Vis Absorption Spectra and *Z*/*E* Photoswitching Behavior of **1–3**

To prepare stock solutions of compounds **1**–**3** for spectroscopic studies, deionized water was used, and the solutions were prepared by stirring in the dark at 20 °C for 16 h. The resulting stock solutions were then stored in the dark at 4 °C prior to spectroscopic measurements; sample solutions were prepared by diluting the stock solutions to the desired concentration and also stored in the dark at 4 °C. Control experiments were conducted to verify the stability of the compounds under these conditions, and it was observed that the compounds remained stable and the relative proportion of the *E* and *Z* azobenzene isomers remained unchanged for over 15 days. In this study, the term “freshly prepared aqueous solutions” refers to the sample solutions prepared and processed following this procedure without irradiation. Prior to conducting spectroscopic measurements, the sample solutions were thermally equilibrated in the dark, and the recording time typically ranged from 4 to 7 min.

**Figure 1:**
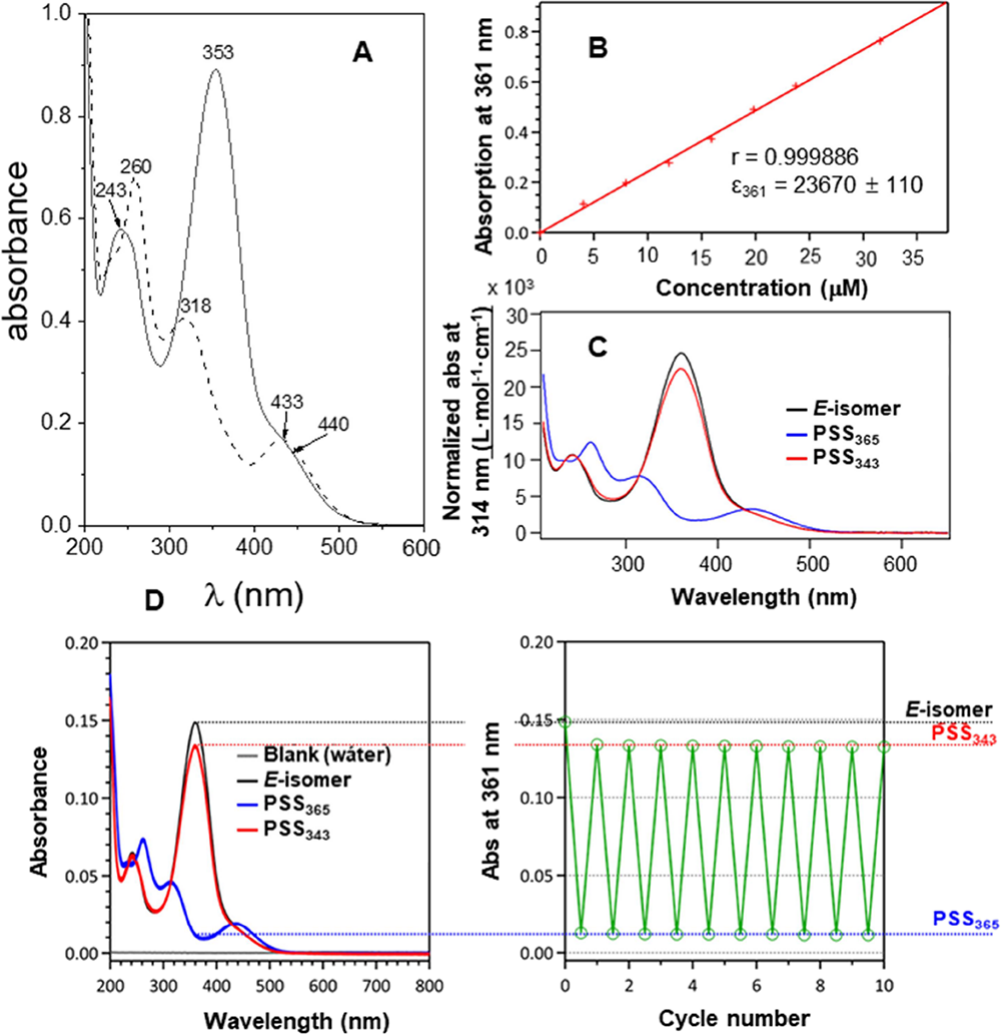
(A) Absorption spectra for 0.5 mM of **1** in H_2_O before (continuous line; *E*-isomer) and after irradiation at 365 nm (dashed line; mostly *Z*-isomer). (B) Determination of the molar absorption coefficient for **2** in H_2_O from the plot of the absorbance at 361 nm vs concentration, and the corresponding (red crosses) linear regression. (C) Normalized spectra of **2** in H_2_O; the black spectrum corresponds to the freshly prepared solution (**2**-*E*), the blue one is recorded after irradiation at 365 nm (mostly **2**-*Z*), and the red one is recorded after irradiation at 543 nm (mostly **2**-*E*). (D) Fatigue resistance of **2** monitored by measuring the intensity of the absorption band at 361 nm under alternate 365 nm (*P* = 7.5 mW·cm^2^)/543 nm (*P* = 5.4 mW·cm^2^) irradiation cycles in H_2_O. Measurements were performed at 25 °C.

The UV–vis absorption spectra of a 0.5 mM freshly prepared aqueous solution of the model compound **1** exhibited two bands centered at approximately 243 and 353 nm and a shoulder located near 440 nm (Figure 1A). Inspection of the corresponding ^1^H NMR spectrum revealed a content of >95% of the *E*-isomer. Upon irradiation at 365 nm, the *E*-isomer partially photoisomerized to the *Z*-form, reaching an approximately 1:4 *E*:*Z* relative proportion at the photostationary state (PSS) as seen by proton NMR (Figure S5, ESI). Control experiments showed that the 365 nm filter affords indeed the best *E* → *Z* photoconversion yield. The absorption spectrum of the resulting aqueous solution showed bands at 260, 318, and 433 nm (Figure 1A). The intense band at 353 nm in the solution before irradiation was attributed to the π → π* transition for the *E*-isomer, which decreased in intensity and shifted to the blue (318 nm) for the *Z*-isomer. The weaker band (or shoulder) located at around 440 nm for the *E*-isomer and 433 nm for the Z-isomer is ascribable to the n → π* electronic transition. From this form, upon irradiation at 456 nm, the *E-*isomer is recovered to a great extent.

The *E*-form of azobenzene derivatives can also be recovered by heating the *Z*-form solution. This thermal process is usually much slower (milliseconds or larger timescale) compared with photochemically promoted isomerization (typically in the picosecond timescale).^26^ To investigate thermal conversion in the present case, freshly prepared aqueous solutions of **1** (>95% *E*-isomer) and samples of the same solutions after irradiation at 365 nm (PSS_365_, ≈80% *Z*-isomer) were stored at different temperatures (5 to 45 °C) for 30 min, and the absorption spectra were recorded. As expected, the thermal **1**-*Z*-to **1**-*E* conversion rate increased with temperature. For instance, the intensity of the n → π* electronic transition band at 433 nm decreased on going from 5 to 45 °C, with a concomitant small displacement to the red. Thermal **1**-*Z* to **1**-*E* conversion at 37 °C was also studied by monitoring the intensity of the π → π* transition band with time, from which a thermal half-life conversion (τ_1/2_) of 384 min was calculated (Supporting Information, Figure S6).

The photochromic properties of the βCyD-azobenzene derivatives **2** and **3** in aqueous solution showed similar features to those above commented for the model compound **1**, indicating that the presence of the cyclooligosaccharide portion does not affect significantly the photochromic behavior of the azobenzene appendage. The corresponding UV–vis normalized absorption spectra, i.e., the spectra divided by the concentration, are shown in Figure 1B,C (normalized spectra were used for the determination of the molar absorption coefficient, ε, according to the Beer–Lambert law, see the Supporting Information for details). The π → π* transition band characteristic of the *E*-isomer was observable at λ_max_ = 361 nm for **2** (ε = 23,673 L·mol^–1^·cm^–1^) and λ_max_ = 363 nm for **3** (ε = 25,907 L·mol^–1^·cm^–1^). Irradiation at 365 nm provoked a decrease of the intensity of this band and the appearance of a new band at 433 nm arising from the n → π* transition of the *Z*-isomer. Four isosbestic points could be observed, located at 234, 247, 314, and 429 nm for **2** and at 236, 246, 317, and 425 nm for **3**. The *Z*:*E* ratio at the PSS was determined by integration of the proton NMR aromatic signals for each stereoisomer (Supporting Information, Figure S7). At PSS_365_, >95% of **2**-*E* or **3**-*E* photoconverted to the corresponding *Z*-isomer. Thermal stability studies at 37 °C (the relevant temperature for future biological activity studies) afforded τ_1/2_ values of 359 and 467 min for **2**-*Z* and **3**-*Z*, respectively (Supporting Information, Figure S8). Alternatively, the *Z*-isomers could be isomerized to the *E*-isomers by irradiation at 543 (or 456) nm. Both photosystems **2** and **3** showed high fatigue resistances: several compound photoswitching cycles could be performed by successive alternate irradiations of the corresponding aqueous solutions at 365 and 543 nm with no observable degradation (Figure 1D; see also the Supporting Information, Figure S9, for the analogous experiments conducted on compound **3**).

### Induced Circular Dichroism

Attempts to investigate the photochromic and supramolecular properties of compounds **1**–**3** by fluorescence spectroscopy provided very limited information due to the efficient photoisomerization of azobenzenes in the photoexcited state (see the Supporting Information, Figure S10).^27^ Alternatively, circular dichroism (CD) studies were conducted. βCyD is a chiral molecule that does not absorb in the UV–vis region and, consequently, it does not exhibit an electronic CD spectrum. An achiral chromophore, such as the azobenzene motif in **1**–**3**, does not yield a CD spectrum either. However, the interaction of the azobenzene chromophore with the βCyD chiral cavity upon formation of a self-inclusion or an intermolecular inclusion complex can induce chirality, leading to CD signals in the region where azobenzene absorbs.^28^ The induced CD (ICD) spectra provide information on the association constants and on the structure of the resulting complexes. Thus, the intensities and signs of the ICD signals exhibit variations based on several factors. These include the extent of the chromophore’s inclusion within the βCyD cavity, as well as the orientation of the electronic transition moment of the guest in relation to the (pseudo)sevenfold axis of βCyD. It is important to note that the C_7_-symmetry applies only to native βCyD in solution or derivatives with homogeneous substitutions at equivalent positions and not to single position-modified derivatives like compounds **2** and **3**. A parallel (perpendicular) orientation gives a positive (negative) ICD band. The sign of the bands is reversed if the chromophore interacts with the cavity but is totally or partially outside located.^29^

#### ICD Spectra for **1**:βCyD Complexes

The UV–vis absorption spectra of aqueous solutions containing the model compound **1** in the presence of βCyD at various concentrations showed similar λ_max_ values to those observed in the spectra of neat **1** in water before irradiation, indicating nearly 100% of the *E*-isomer. After irradiation at 365 nm, mostly the *Z*-isomer was present. Similarly, the **1**-*Z* isomer in βCyD-containing samples converted back to the **1**-*E* isomer upon irradiation at 456 nm for 5 h. The intensities of the main band at 360 nm in the latter solutions, when βCyD was present at each concentration, were slightly lower compared to the intensities observed before irradiation at 365 nm for the initial βCyD solutions. However, plotting absorbance against [βCyD] in both cases did not reveal significant changes (Supporting Information, Figure S11). It is inferred that this magnitude is not very sensitive to complexation.

**Figure 2:**
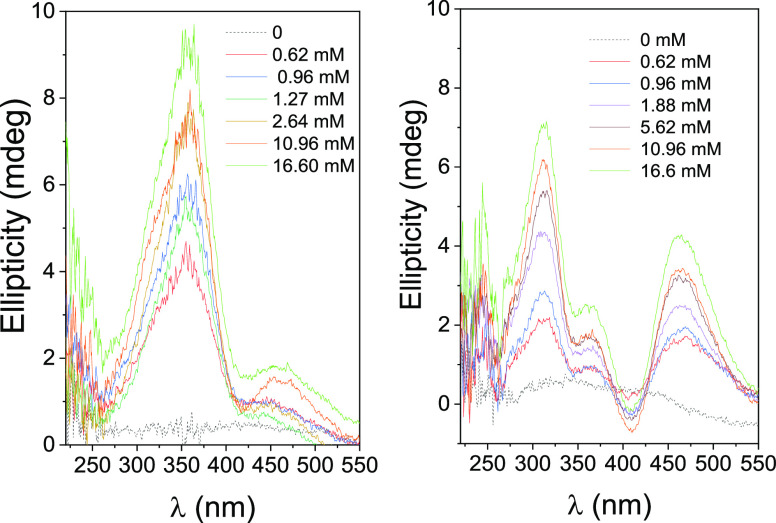
ICD spectra (ellipticities versus wavelength plots) for the freshly prepared diluted solutions of **1** (*E*-isomer) in H_2_O, in the absence (dashed lines) and in the presence of increasing concentrations of βCyD before (left panel), and after irradiation to reach PSS_365_ (mostly *Z*-isomer; right panel) at 25 °C.

Figure 2 shows ICD spectra for freshly prepared aqueous solutions of **1** (0.094 mM constant concentration) in the presence of βCyD at different concentrations ranging from 0 to 16.6 mM, and after irradiation at 365 nm at 25 °C. Analogous experiments were conducted at 5, 15, and 35 °C (Supporting Information, Figures S12 and S13). Ellipticity values remained rather low irrespective of the major isomeric form present in the sample (*E* or *Z*, respectively), with bands located at positions similar to those distinguishable in their respective absorption spectra. Thus, **1**-*E*/βCyD samples exhibited a main positive band at ∼360 nm and a less intense one at ∼455 nm. Upon irradiation at 365 nm, two positive bands for **1**-*Z*, located at around ∼313 nm and near ∼460 nm were noticed, together with a band at 360 nm that can be attributed to the remaining proportion of the *E*-isomer in the solution (20%). The fact that the intensity of this band only slightly increased with temperature correlates with the low thermal conversion of the *Z*-isomer to the most stable *E*-form during the runtime of the experiments. The positive sign of the bands in the ICD spectra of **1**-*E* and **1**-*Z* supports that both stereoisomers form inclusion complexes with βCyD. In these complexes, the dipole moments associated with the S_0_ → S_1_ (n → π*) and S_0_ → S_2_ (π → π*) transitions, which are nearly colinear in the *E*-isomer and differ by 15.5 degrees in the *Z*-isomer (as shown in the Supporting Information, Figure S32), orient quasi-parallel to the βCyD axis. The intensity of the π → π* band increased with temperature, while the intensity of the n → π band exhibited the opposite trend. However, the ratio between the two bands remained essentially unchanged regardless of the concentration of βCyD (Supporting Information, Figure S14). In contrast, the individual ellipticity intensities increased with [βCyD] at each temperature, unambiguously corroborating that both the *E*- and *Z*-forms interact with the cyclooligosaccharide. The ellipticities at the most intense π → π* transitions, placed at λ_max_ 360 nm for **1**-*E* and 313 nm for **1**-*Z*, represent an absolute measure of the fraction of the corresponding isomer that is complexed with βCyD.

**Figure 3:**
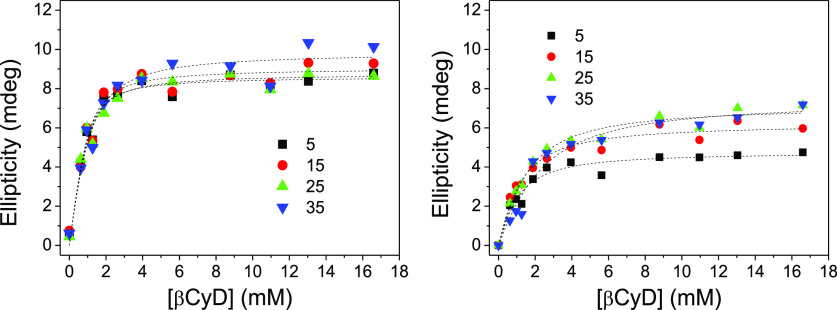
Ellipticities vs βCyD concentration at different temperatures, for **1** (0.094 mM)/βCyD aqueous solutions before (left panel; *E*-isomer) and after irradiation to reach PSS_365_ (right panel; mostly *Z*-isomer) obtained from the analysis of the corresponding ICD spectra.

Figure 3 depicts the plots of ellipticity changes (in mdeg) for the above **1**-*E* and **1**-*Z* aqueous solutions ([**1**] = 0.094 mM) versus the concentration of βCyD. As advanced, no signal was detected in the absence of βCyD. Ellipticities increased with [βCyD] and approached a plateau at the highest βCyD concentration values. Least squares fitting of the experimental data (see the Supporting Information, eq S3) were consistent with 1:1 complex stoichiometries (see also the Supporting Information, Figure S15, for linear fittings of the data to eq S4) and yielded the corresponding association constant (*K*) at each temperature. Values at 25 °C were *K* = (14.5 ± 2.0) × 10^2^ and (6.1 ± 0.7) × 10^2^ M^–1^ for the **1**-*E*:βCyD and **1**-*Z*:βCyD complex, respectively. *K* values at other temperatures are collected in Table 1. Although complexation of **1** with βCyD is more favorable in the *E* than in the *Z*-form, no strong differences are observed in the affinity constants from a global standpoint taking into account the accuracy of the determined values. This is in line with the literature reports on complexes of water-soluble azobenzene derivatives and βCyD.^12^

**Table 1:** Association Constants for the **1**:βCyD (1:1) Complexes at Different Temperatures Obtained from the Nonlinear Analysis (Supporting Information, Eq S3) of ICD Spectra of the **1**/βCyD Solutions before Irradiation (*K_E_*) and after Irradiation at 365 nm (*K_Z_*)

temperature (°C)	10^–2^ *K_E_* (M^–1^)	10^–2^ *K_Z_* (M^–1^)
5	16.4 ± 2.6	9.6 ± 1.8
15	14.7 ± 2.7	8.5 ± 1.1
25	14.5 ± 2.0	6.1 ± 0.7
35	9.8 ± 1.2	3.9 ± 0.8

Table 2 collects the thermodynamic parameters obtained for the complexation of the *E* and *Z*-forms of the model compound **1** with βCyD from the linear van’t Hoff equation (Supporting Information, Figure S16). Both are enthalpy-driven association processes (Δ*H*^0^ < 0), which should be attributed to the interplay of attractive van der Waals, electrostatic and C–H···π interactions. These are strongly dependent on the relative size/shape between host and guest molecules and the geometry of the host–guest complex.^30^ As concerns the entropic term, it is favorable (*T*Δ*S*^0^ > 0) for the *E*-isomer but negatively contributes to complex formation in the case of the *Z*-isomer (*T*Δ*S*^0^ < 0). The sign of Δ*S*_0_ is typically determined by the balance of two opposing effects. On the one hand, there is the loss of rotational and translational degrees of freedom, which contributes positively to the entropy change. On the other hand, there is the disruption of water shells that solvate the inner macroring cavity and the guest during the complexation process, resulting in a negative entropic term. Typically, the second one predominates if the flexibility of the cavity is not much reduced upon deep inclusion of the guest in the βCyD cavity. The data suggest that this is the case for the **1**-*E*/βCyD complex, but not for the **1**-*Z*/βCyD complex. Indeed, there are computational and experimental precedents showing that “straight” *E*-azobenzene derivatives preferentially form axial-type deep inclusion complexes with βCyD and that the flexibility of the cavity before and after the inclusion remains pretty the same.^31^ Differently, “folded” *Z*-azobenzene derivatives can interact closer to the cavity entrance, with a lower number of atoms found inside, significantly reducing host flexibility without benefiting from a strong hydrophobic effect.

**Table 2:** Δ*H*^0^ and Δ*S*^0^ Values for the **1**:βCyD Complexes Formed in Water before Irradiation (∼100% of *E*-Isomer) and after Irradiation at 365 nm (∼80% of *Z*-Isomer)

thermodynamic parameter	Δ*H*^0^ (kJmol^–1^)	Δ*S*^0^ (J K^–1^ mol^–1^)
*E*-isomer	–11.1 ± 4.2	+22.1 ± 14.3
*Z*-isomer	–21.7 ± 4.3	–20.2 ± 14.6

To further assess the stability of **1**:βCyD complexes, the ICD spectra for a freshly prepared aqueous solution of **1**-*E* (0.094 mM) containing βCyD (8.31 mM) in the absence and in the presence of increasing amounts of 2-aminoadamantane hydrochloride (AdaNH_2_) were monitored. Water-soluble adamantane derivatives form strong inclusion complexes with βCyD (*K* > 10^4^ M^–1^) very well suited for competition experiments.^32^ These solutions were later irradiated at 365 nm to promote photoconversion into the *Z*-isomer (PSS_365_), and the spectra were recorded again. In the absence of AdaNH_2_, the fraction of *E* (*Z*) isomer in complex with βCyD before (after) irradiation at 25 °C was 0.92 (0.83), as determined from the corresponding association constant values. In both cases, the addition of AdaNH_2_ led to a significant decrease in the intensity of the characteristic ICD bands (Supporting Information, Figure S17). This observation indicates that the inclusion of the adamantane guest in the βCyD cavity competes with the formation of both the **1**-*E*:βCyD and **1**-*Z*:βCyD complexes.

#### ICD for βCyD-Azobenzene Derivatives **2** and **3**: Dimer Formation

**Figure 4:**
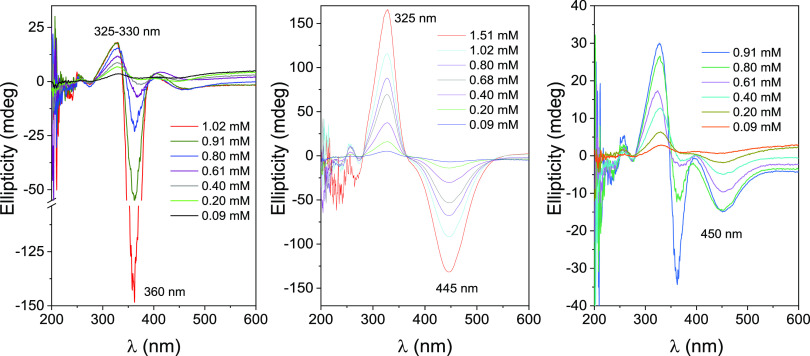
ICD spectra (ellipticities versus wavelength plots) of the freshly prepared aqueous solutions of **3** at different concentrations in H_2_O before irradiation (left panel; *E*-isomer), after irradiation to reach PSS_365_ (middle panel; mostly *Z*-isomer) and after irradiation of the latter solution 456 nm for 5 h (right panel; mostly *E*-isomer). Measurements were performed at 25 °C.

The above data unequivocally evidence that the triazolyl-azobenzene motif can undergo inclusion complex formation with βCyD in either the *E* or *Z*-form. Our next goal was to determine whether or not attaching this motif to the secondary rim of βCyD affects the supramolecular properties. ICD spectra for freshly prepared aqueous solutions of **3** (exceedingly major **3**-*E* isomer; 0.095 to 1.51 mM concentrations) at 25 °C are depicted in Figure 4 (left panel). The spectra show a weak negative band centered very close to the n → π transition (∼450 nm). The band corresponding to the π → π* transition at ∼360 nm was not observed even when measurements were performed at rather low concentrations ([**3**] = 6 × 10^–6^ M). Instead, this band split into a positive band centered at 325–330 nm and a negative, more intense band at 360–365 nm. The intensity of both bands, especially the negative one, increased with concentration. This bisignal can be attributed to an exciton coupling (EC), which occurs when two chromophores are close in the space and at least one of them exhibits relatively large molar absorptivity.^33^ It must arise from the intermolecular interaction between two azobenzene modules, supporting the formation of a face-to-face **3**-*E*-isomer dimeric species.^34^

Photoisomerization of the above **3**-*E* solutions by irradiation at 365 nm resulted in concomitant disappearance of the EC bisignal in the ICD spectra. Instead, rather symmetrical positive and negative bands centered at 325 and 445 nm, ascribable to the **3**-*Z*-isomer π → π* and n → π transitions, were detected (Figure 4, middle panel). Upon subsequent irradiation at 456 nm, the initial EC signal was partially restored, resulting in bands in the ICD spectra that resembled those of the initial solutions. However, the intensity of these bands was lower compared to the original spectra (Figure 4, right panel). A similar behavior was deducted when studying the ICD spectra of aqueous solutions of the nonmethylated βCyD-azobenzene compound **2**, with just some shifts of the corresponding bands to the blue (Figure S18, ESI).

**Figure 5:**
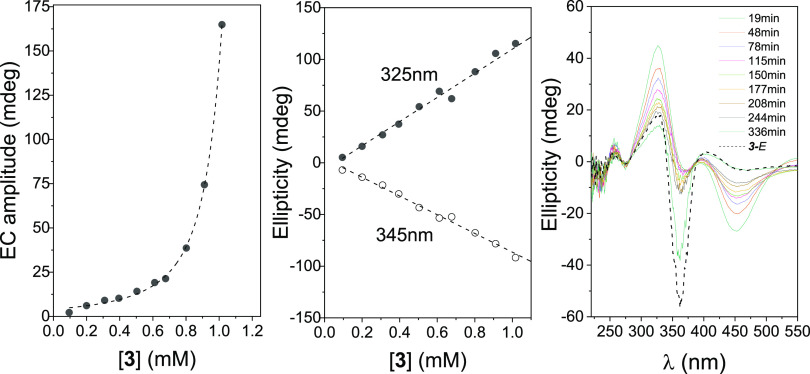
Exciton coupling amplitude obtained from ICD spectra for the freshly prepared aqueous solutions of **3** at different concentrations (*E*-isomer; left panel), ellipticity values for the main bands obtained after irradiation at 365 nm (mostly *Z*-isomer; middle panel) versus [**3**], and ICD spectra monitored at different times for 0.911 mM **3** in H_2_O after irradiation at 365 nm (mostly *Z*-isomer; right panel; the curved labeled “**3**-*E*” corresponds to the spectrum for the freshly prepared aqueous solution of **3** before irradiation (*E*-isomer) under the same experimental conditions).

Figure 5, left panel, shows the variation of the EC amplitude (total intensity, sum of both positive and negative bands) with increasing concentrations of **3**, obtained from the analysis of ICD spectra of freshly prepared aqueous solutions (**3**-*E*-isomer). The nonlinearity of the representation further supports the understanding that the observed EC signal does not originate from intramolecular phenomena. Instead, it is primarily attributed to the interaction between the azobenzene moieties of two molecules arranged in a supramolecular dimer with antiparallel orientations. The relative proportion of these supramolecular dimers in the solution is expected to be concentration-dependent. Sharply differently, ellipticities measured at the maximum of the π → π* and n → π main transition bands after irradiation at 365 nm (Figure 5, middle panel) exhibited linearity with concentration. This is consistent with dimer dissociation following *E*-to-*Z* photoisomerization. The ellipticities then arise exclusively from the intramolecular interaction of the azobenzene module with the βCyD macroring in **3**-*Z* individual molecules. The process is reversible: either irradiation- (456 nm) or thermal-promoted *Z*-to-*E* conversion was accompanied by the recovery of EC due to dimer formation. Thermal isomerization was actually as efficient as for the model azobenzene derivative **1**. From the increase in the intensity of the negative EC band measured at 360 nm with time, a thermal **3**-*Z* half-life of τ_1/2_ ≈ 420 min at 37 °C was estimated (Figure 5, right panel). The *E*-isomer of **2** forms a stable head-to-head dimer in aqueous solution, where the two azobenzene modules are closely positioned. Upon photoswitching to the *Z*-isomer, the dimer dissociates, thus demonstrating an interesting light-sensitive, self-assembling prototype.

Unfortunately, it was not possible to determine an association constant for dimer formation by analyzing the amplitude of the EC signal against compound concentration plots (Figure 5, left panel) because the ICD signal in the EC zone was influenced not only by intermolecular interactions between azobenzene moieties but also by intramolecular effects involving azobenzene-βCyD interactions.

**Figure 6:**
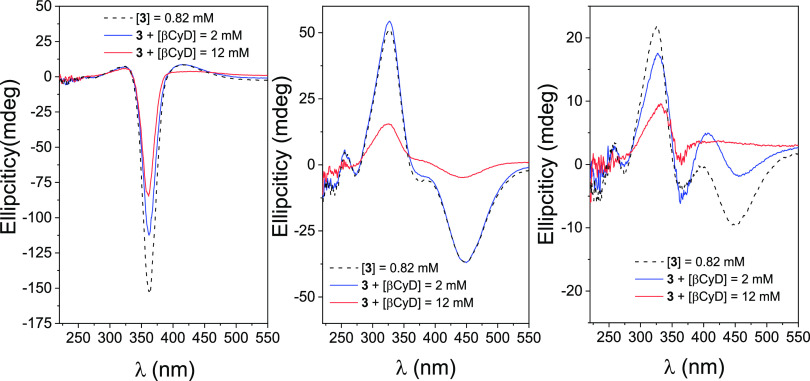
ICD spectra for a 0.82 mM freshly prepared aqueous solution of **3** (major *E*-isomer) in the absence and in the presence of βCyD at 2 and 12 mM concentrations (left panel), idem for the same solutions after irradiation at 365 nm (mostly *Z*-isomer, middle panel) and after later irradiation of the previous solutions at 456 nm (mostly *E*-isomer, right panel).

To get a deeper view on dimer stability, we first conducted competition experiments with compound **3** (Figure 6 and Supporting Information, Figure S21) and βCyD. Thus, a 0.82 mM aqueous solution of **3**-*E* was freshly prepared, to which βCyD was added to reach 2 and 12 mM concentrations. The EC intensity of the prominent negative signal at 360 nm (and of the positive one at 320 nm) was only reduced by approximately 40% at the highest βCyD concentration, meaning that the head-to-head (**3**-*E*)_2_ homodimer is rather stable. The above solutions were next irradiated (365 nm) to provoke the isomerization of **3**-*E* to **3**-*Z*. No noticeable changes were observed in the ICD spectra obtained in the absence and presence of βCyD at a concentration of 2 mM, suggesting that the **3**-*Z* isomer does not form significant heterodimer species with βCyD, unless a large excess of βCyD (12 mM) is used. Irradiation at 456 nm of these βCyD-containing solutions resulted in partial recovery of the EC signal, indicating that the presence of the competing βCyD host did not hinder reversible photoisomerization or the formation of (**3**-*E*)_2_ dimers. A more quantitative study of the potential formation of heterodimers between **3**, in the *E-* o *Z*-form, and βCyD was performed using a 10 mm path cell, allowing to work with lower concentrations of the βCyD-azobenzene derivative. The corresponding ICD spectra of a freshly prepared 0.1 mM solution of **3**-*E* in water at 25 °C, in the absence of βCyD, still showed a weak EC bisignal, denoting the presence of a small fraction of dimer even at such dilution (Supporting Information, Figure S21, left panel). Upon βCyD addition, the intensity of the EC band decreased and the spectra evolved to finally exhibit two positive bands at around 360 and 440 nm at the highest βCyD concentration. This scenario closely resembles the previous findings observed for the inclusion complex between the model compound **1**-*E* and βCyD (Figure 2). These similarities strongly suggest that the azobenzene module in **3**-*E* can also enter the βCyD cavity, leading to the formation of an inclusion-type heterodimer. A similar competition experiment was performed after irradiating the 0.1 mM solutions of **3**, both in the absence and presence of βCyD, at 365 nm to induce *E*-to-*Z* photoisomerization (Supporting Information, Figure S21, right panel). The data revealed a similar trend: the symmetrical positive and negative bands in the ICD spectra of **3**-*Z* transformed into two positive bands, located at λ_max_ ≈ 312 and ≈450 nm, in the presence of an excess of βCyD. This observation mirrors the behavior observed for the complexation of **1**-*Z* with βCyD (Figure 2), providing further support for the notion that the azobenzene module in **3**-*Z* can also interact with βCyD and form a heterodimer. Fitting the change of the n → π* transition band intensity with [βCyD] (Supporting Information, eq S3) yielded association constants *K* = 450 ± 110 and 600 ± 60 M^–1^ at 25 °C for the **3**-*E*:βCyD and **3**-*Z*:βCyD complexes, respectively, compared to 1450 ± 200 and 610 ± 70 M^–1^ for the corresponding **1**-*E*:βCyD and **1**-*Z*:βCyD complexes (Table 1). Efficient inclusion of the azobenzene appendage of **3** into the cavity of the neighboring βCyD in the **3**-*E*:βCyD heterodimer is likely hampered by steric hindrance when the intervening macrorings approach each other. Conversely, the association constant of compound **3** in the *Z*-form with βCyD is identical to that of the model compound **1**-*Z*. In other words, the ability of the *Z*-triazolylazobenzene motif to interact with native βCyD remains unaffected even after being coupled to the secondary *O*-2 position of another βCyD. This further supports the notion that complexation in this case does not involve deep inclusion within the βCyD cavity.

**Figure 7:**
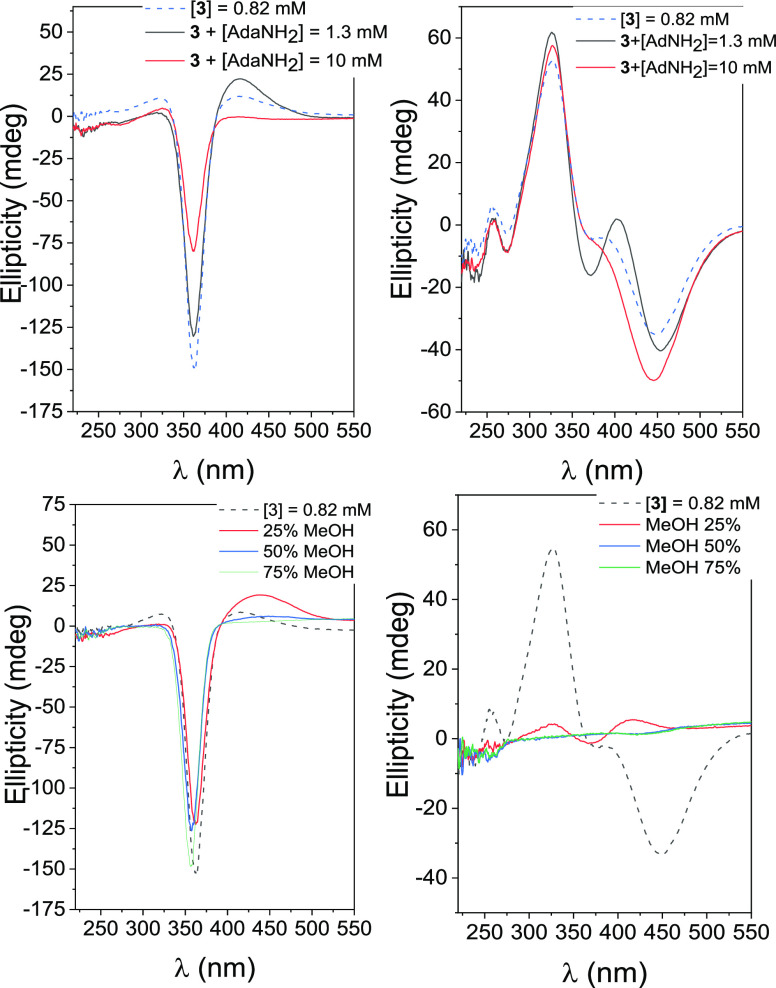
ICD spectra for a 0.82 mM freshly prepared aqueous solution of **3** (*E*-isomer) in the absence and in the presence of AdaNH_2_ at 1.3 and 10 mM concentrations (upper-left panel); idem for the same solutions after irradiation at 365 nm (mostly *Z*-isomer; upper-right); ICD spectra for a 0.82 mM freshly prepared aqueous solution of **3** (*E*-isomer) in mixtures of water/methanol with different MeOH contents (% v/v; bottom-left panel); idem for the same solutions after irradiation at 365 nm (bottom-right panel).

The effect of the AdaNH_2_ guest on the self-assembling properties of compound **3**, which competently dissociated the complexes of the model azobenzene derivative **1** with βCyD in both the *E-* and *Z*-forms, was subsequently examined. As observed in the ICD spectra shown in Figure 7 (upper-left panel), the (**3**-*E*)_2_ dimer in a freshly prepared aqueous solution of **3** (0.82 mM) did not completely dissociate even in the presence of a large excess (10 mM) of AdaNH_2_. This finding is somewhat counterintuitive, considering the strong affinity of the adamantane motif toward the βCyD cavity, which is higher than the estimated affinity for the azobenzene moiety of **3**-*E* in the aforementioned heterodimer formation experiment. It can be concealed assuming that formation of (**3**-*E*)_2_ may not require a deep reciprocal inclusion of the azobenzene modules and is compatible with the presence of the adamantane guest inside the cavity of the βCyD component. No significant changes were observed in the ICD spectra of the irradiated solution of **3** (mostly 3-*Z*) upon the addition of AdaNH_2_, indicating the presence of monomolecular species where the relative orientation of the azobenzene and βCyD moieties remains essentially unchanged, regardless of the occupancy of the cavity by the AdaNH_2_ guest (Figure 7 upper-right panel).

In a further series of experiments, the effect of solvent polarity on (**3**-*E*)_2_ dimer formation was assessed by checking the changes in the ICD spectra of freshly prepared solutions of **3** (0.82 mM) in water/methanol mixtures (Figure 7, bottom-left panel). Reducing the polarity of the solvent by increasing the proportion of methanol significantly reduces the propensity of hydrophobic motifs to undergo inclusion. This is because the substrate experiences an environment in solution that resembles the microenvironment provided by the cyclodextrin cavity. However, even in a 75% (v/v) aqueous methanol solution, the negative EC signal characteristic of the dimer only slightly decreases, indicating that inclusion is likely not the main driving force behind the dimerization of **3**-*E*. In contrast, the bands in the ICD spectrum of aqueous **3**-*Z* quickly disappear upon the addition of methanol to the medium (Figure 7, bottom-right panel). It becomes evident that the appended azobenzene moiety of **3**-*Z* interacts intramolecularly with the βCyD secondary rim, without fully penetrating the cavity, but this interaction is shielded in a less polar solvent. The EC bisignal is partially recovered after irradiation at 456 nm.

### Supramolecular and Structural Investigations by NMR

**Figure 8:**
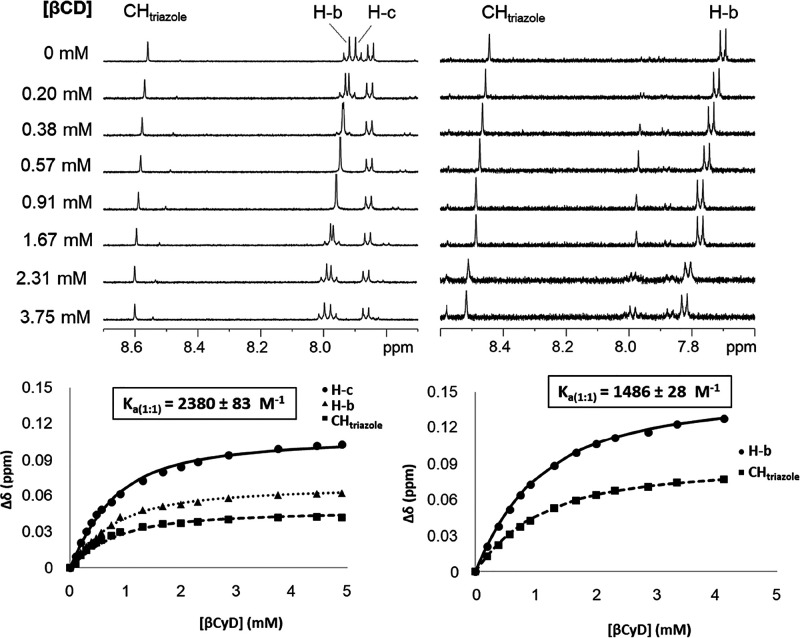
Selected region (triazole CH and aromatic protons) of the ^1^H NMR spectra of 0.6 mM solutions of **1**-*E* (upper-left) and **1**-*Z* (upper-right) in D_2_O in the absence and in the presence of increasing amounts of βCyD; the corresponding binding isotherm (titration) plots, with indication of the resulting association constant for the corresponding 1:1 complexes, are also depicted (lower left and right panels, respectively).

Addition of increasing amounts of βCyD to a 0.6 mM solution of **1**-*E* in D_2_O provoked ^1^H NMR chemical shift displacements affecting all aromatic resonances as well as the triazole, the methylene and the methyl group proton signals (Figures 8 and S22, ESI). The chemical shifts for the H-3 and H-5 protons of βCyD, located inside the cavity, and the methylene H-6a,b signals likewise underwent significant movement in the presence of the guest, a scenario that is characteristic of deep inclusion complex formation (Figure S23, ESI). A parallel titration experiment starting from an irradiated (365 nm) solution of **1** in D_2_O, containing mostly **1**-*Z*, let inferred a similar conclusion (Figures 8, S24, and S25, ESI). Least-square fitting of the corresponding binding isotherms at 25 °C afforded *K* values of 2380 ± 83 and 1486 ± 28 M^–1^, respectively, for the corresponding 1:1 hot-guest binding equilibria. Although these values are higher than *K* values determined by ICD (1450 ± 200 and 610 ± 70 M^–1^), the data show identical trends: both the *E*- and *Z*-isomers of the triazolylazobenzene model compound **1** can enter the βCyD cavity and form inclusion complexes with affinities that are comparable.

**Figure 9:**
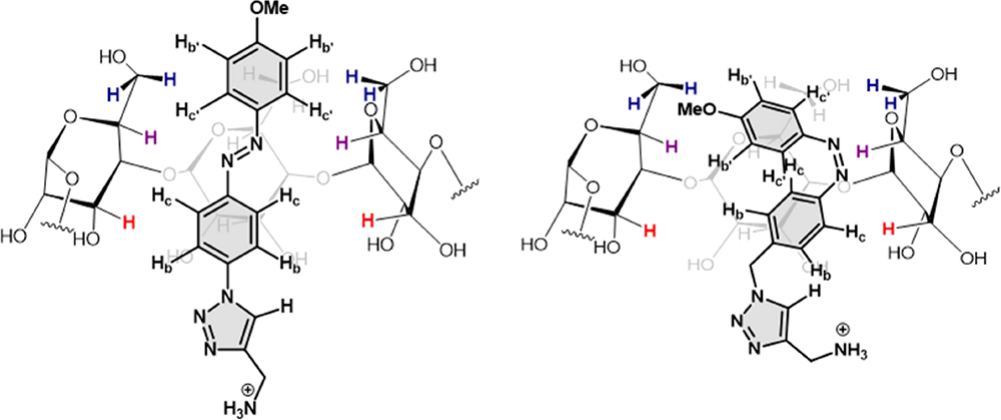
Schematic representation of **1**-*E* (left) and **1**-*Z* (right) inside the cavity of βCyD (only three monosaccharide residues are shown) according to the chemical shift displacements observed in the corresponding titration experiments and the intermolecular NOE contacts. The βCyD protons H-3, H-5, and H-6a, b are colored in red, magenta, and deep blue, respectively.

Attempts to get structural information on the orientation of the guest in the cyclooligosaccharide interior by NOESY spectra were hampered by the fact that the molecular weight of the supramolecular 1:1 complex falls within the very small-to-zero NOE molecular weight range (700–1200 Da).^35^ Nevertheless, tiny cross-peaks between the signals of the aromatic protons H_c_ and H_c’_ and the signal for the H-5 protons of βCyD were observable in the case of **1**-*E* (Supporting Information, Figure S26). These contacts are compatible with the insertion of the azobenzene system longitudinally to the main βCyD axis, with the phenyl ring bearing the methoxy groups pointing to the primary rim and the hydrophilic aminomethyltriazole moiety to the secondary rim (Figure 9, left). In the case of **1**-*Z*, all aromatic azobenzene protons seemed to have NOE contacts with H-5 βCyD protons (Supporting Information, Figure S27). Indeed, the folded disposition of the azobenzene segment in the *Z*-isomer places the protons on the two aromatic rings in proximity. A tiny H_b_/H-3 cross-peak is also observed, suggesting that the polar portion orients toward the wider rim, as in the **1**-*E*:βCyD complex (Figure 9, right).

**Figure 10:**
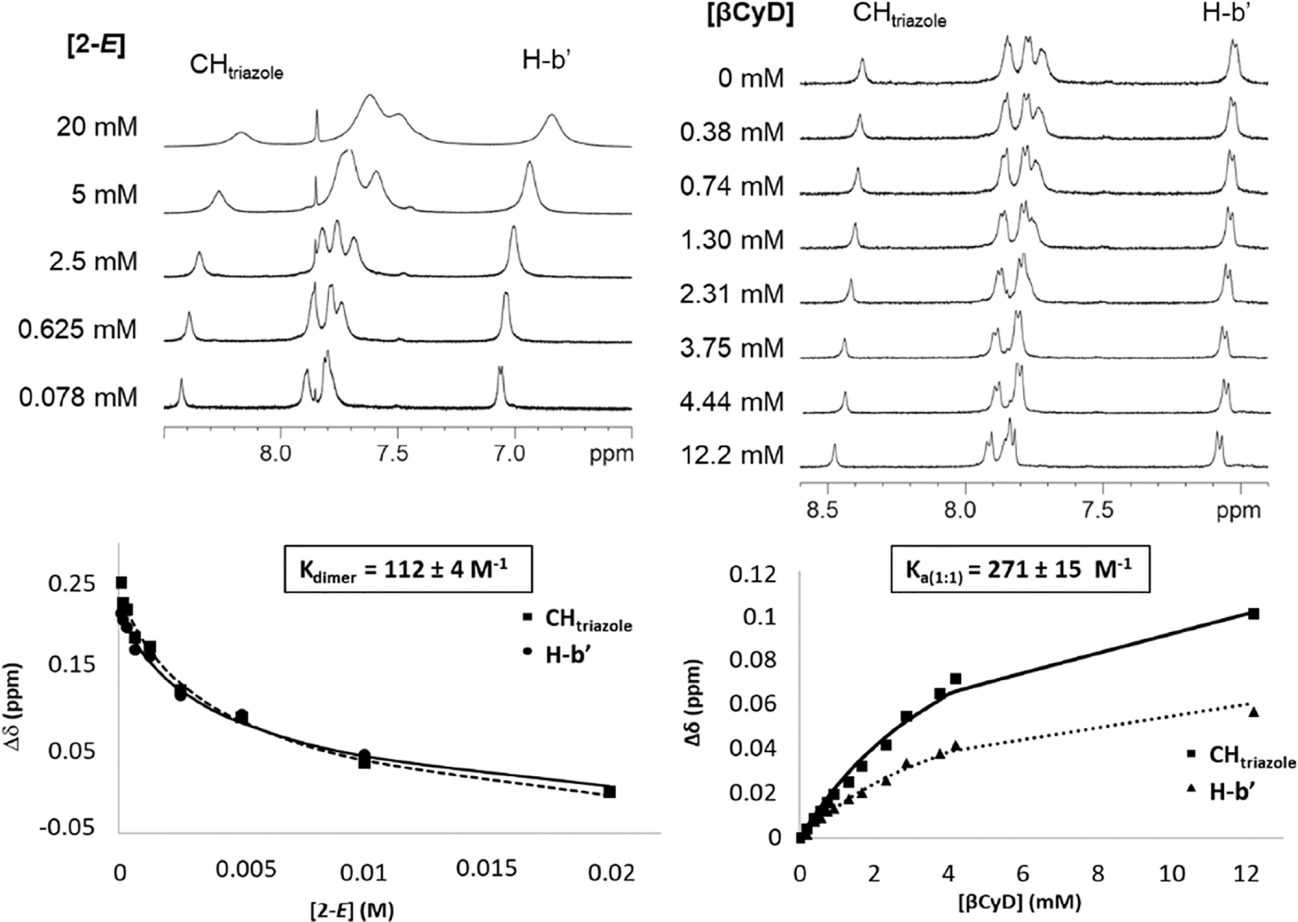
Selected region (triazole CH and aromatic protons) of the ^1^H NMR spectra of **2**-*E* in D_2_O upon serial dilution experiments (upper-left) and upon addition of increasing amounts of βCyD to a 1 mM solution (upper-right); the corresponding homo and heterodimerization binding isotherms, with indication of the resulting association constants, are also shown (lower left and right panels, respectively).

^1^H NMR serial dilution experiments in D_2_O confirmed significant differences in the self-assembling behavior of the βCyD-azobenzene derivative **2**, which possesses the same triazolylazobenzene module as the model compound **1**, depending on its configurational status. The spectra of **2**-*E* exhibited noticeable line broadening across the tested concentration range (20 to 0.078 mM), indicative of dynamic aggregation equilibria that occur relatively slowly on the NMR timescale (Figure 10, left; see Supporting Information, Figure S28 for the complete data set). Line broadening decreased upon dilution, accompanied by chemical shift displacements. The data were well-fitted to a 1:1 dimerization equilibrium, yielding a dimerization constant (*K*_d_) of 112 ± 4 M^–1^. Upon photoisomerization of **2**-*E*, the resulting 20 mM D_2_O solution primarily composed of **2**-*Z* formed a precipitate that dissipated upon dilution. ^1^H NMR spectra were recorded in the range of 10–0.156 mM, and line resolution improved with dilution, but no apparent chemical shift changes were observed in this case (Supporting Information, Figure S29). While the possibility of soluble aggregates forming at high concentrations and subsequently precipitating cannot be completely ruled out, the collective results support the notion that **2**-*Z* exists primarily in a monomeric form in aqueous solution.

**Figure 11:**
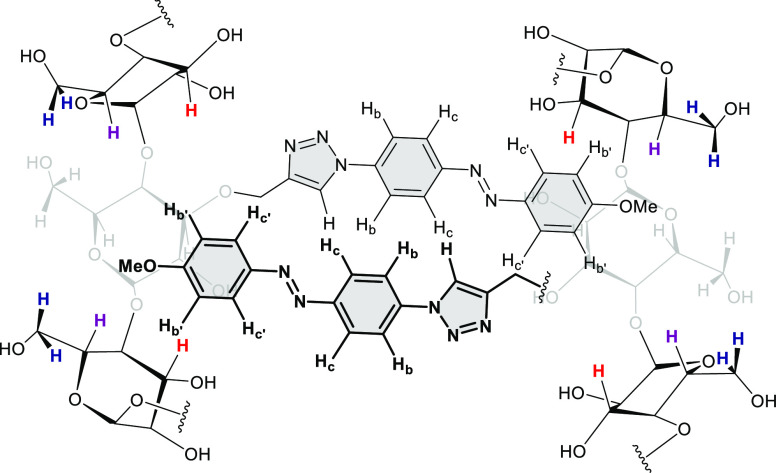
Schematic representation of **2**-*E* dimer structure showing the triazolylazobenzene modules barely included in the cavity of the neighbor cyclooligosaccharide (only three monosaccharide residues are shown) but remaining largely outside, in close proximity, as inferred from serial dilution NMR and NOE contacts. The βCyD protons H-3, H-5, and H-6a,b are colored in red, magenta, and deep blue, respectively.

The addition of βCyD to a 1 mM solution of **2**-*E* in D_2_O led to chemical shift displacements of the aromatic and triazole proton signals that are consistent with the formation of heterodimers in competition with homodimers (Figure 10, right; see the Supporting Information, Figure S30, for the full set of data). The apparent association constant for **2**-*E*:βCyD complexation obtained from the corresponding chemical shift versus [βCyD] plots was 271 ± 15 M^–1^, in the same order of magnitude as the homodimerization constant but one order of magnitude lower than the *K* value for **1**:βCyD complex formation. It can be inferred that the *E*-configured triazolylazobenzene appendage covalently linked to the secondary rim of βCyD is less prone to undergo efficient inclusion in the cavity of a second βCyD entity. In further support, the NOESY spectrum of a 5 mM solution of **2**-*E* in D_2_O evidenced NOE contacts between the aromatic protons of the terminal phenyl ring (H_b′_ and H_c′_) with the H-3 βCyD proton, but not (or very week) between the internal phenyl ring or triazole protons and βCyD protons (Supporting Information, Figure S31). This finding suggests that the triazolylazobenzene modules in the dimer predominantly reside outside the internal cavity of βCyD, in close proximity to each other, presumably involved in aromatic–aromatic interactions. This further confirms the conclusions drawn from the ICD data (Figure 11).

### Molecular Mechanics and Molecular Dynamics Calculations

We have used molecular modeling to complement the experimental results on the supramolecular properties of the systems under investigation. The choice of the force field for computational studies on cyclodextrin complexes is delicate.^36^ We have previously found that the Sybyl X-2.0 and the Tripos force field^37^ satisfactorily reproduced the experimental results for the inclusion of hydrophobic guests in the βCyD cavity, although it tends to overestimate the binding energies.^38^ Lindhorst and coworkers have also used this force field to investigate structural and protein binding aspects of glycosylazobenzene derivatives.^39^ Based on these antecedents, we decided to apply it in the context of this work.

#### Complexation of the Model Compound **1** (*E* and *Z*-Isomers) with βCyD

**Figure 12:**
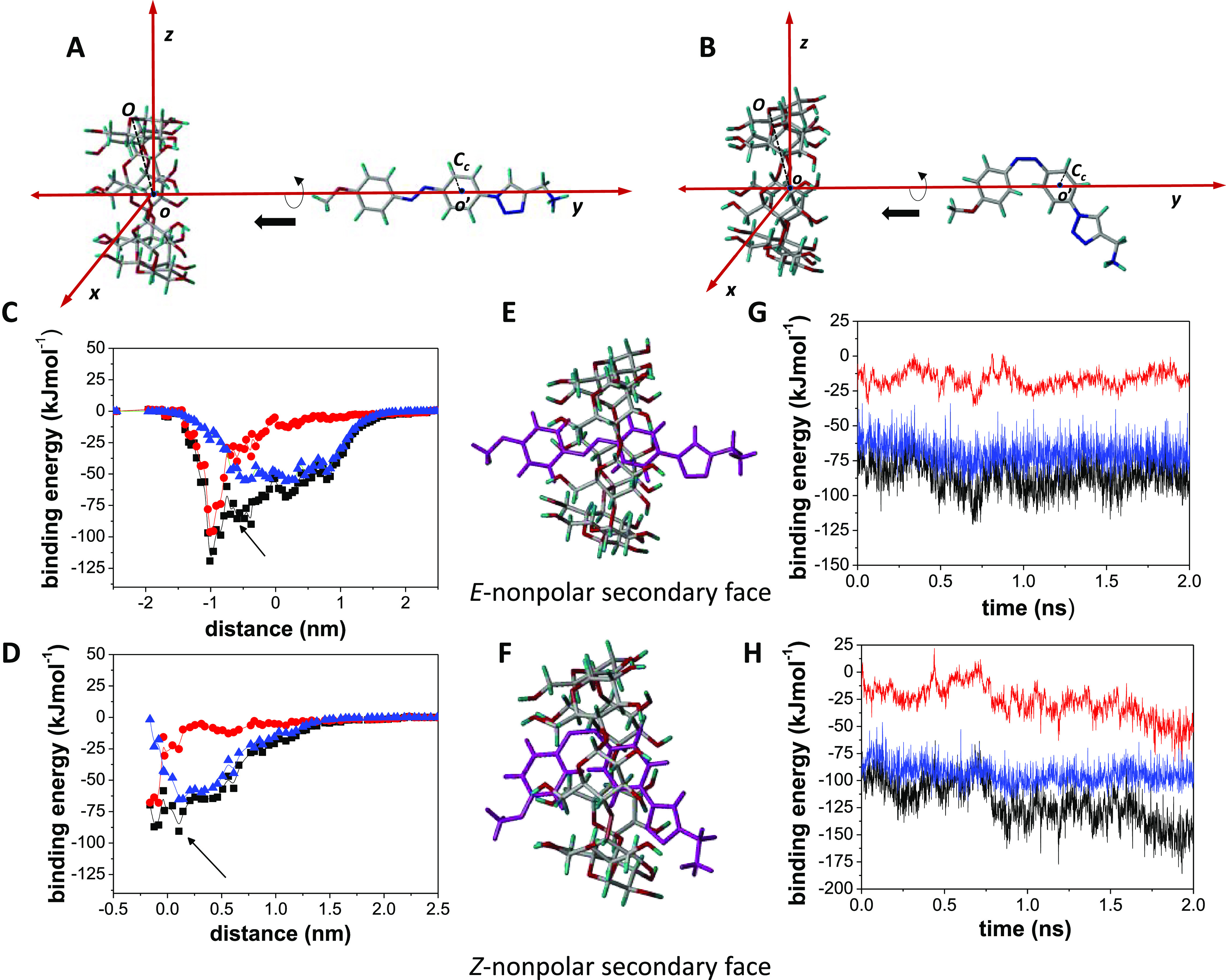
(A) and (B) Coordinate system used for **1**-*E* and **1**-*Z* approaching to βCyD by the secondary face along the *y* coordinate through the nonpolar (methoxyphenyl) end. (C) and (D) Total **1**-*E*/βCyD and **1**-*Z*/βCyD binding energies (black squares) and electrostatics (red circles) and van der Waals (blue triangles) contributions, respectively, as a function of the *o*–*o*′ distance along the *y* coordinate, for the **1** (*E* or Z isomer)-to-βCyD approaching according to A (**1**-*E*) and B (**1**-*Z*). (E) and (F) Optimal MBE structures, indicated by the arrows in C and D. (G) and (H) Histories for **1**-*E*/βCyD and **1**-*Z*/βCyD total binding energies (black) and electrostatics (red) and van der Waals (blue) contributions obtained from the analysis of 2 ns MD trajectories performed on the MBE (C and D) structures, respectively.

While computational approaches have been extensively used to study the isomerization mechanism of azo moieties in various contexts, the investigation of the inclusion of azobenzene derivatives within macrocyclic hosts remains relatively scarce in the literature.^14^ We therefore settled to analyze the inclusion of the model triazolylazobencene derivative **1** in the βCyD cavity by means of molecular mechanics (MM) and molecular dynamics (MD) simulations conducted in explicit water (see the Supporting Information for details; the information on the periodic box size and the number of water molecules in each MD experiment is given in the corresponding figure legends). In the first calculation, a molecule of βCyD was located with its center of mass (*o*) at the origin of a coordinate system and oriented along the *y* axis. Next, an *E*- or *Z*-isomer of **1** was approximated along the *y* coordinate axis, keeping its center of mass (*o*′) on that axis, with different orientations and from distances at which **1** and βCyD barely interacted. The process for two representative orientations, namely, the azobenzene derivative approaching the secondary face of the βCyD host through the nonpolar methoxyphenyl extremity is schematized in Figure 12A,B (see the Supporting Information, Figure S33, for the schematic representation of the whole set of **1**-*E*- or **1**-*Z* to βCyD approaches considered in our study). The most favorable **1**-*E*- or **1**-*Z* relative to βCyD orientations for approaching was obtained by scanning the *O-o-o*′*-C_c_* dihedral angles, where *O* and *C_c_* are a glycosidic *O*-4 oxygen and the aromatic carbon indicated in Figure 12A,B, and obtaining binding energies as a function of the *o*–*o*′ distance in the absence of water. Once the *O-o-o*′*-C_c_* dihedral angle fixed, the structures generated by scanning the *o–o*′ distances from 2.5 to −2.5 nm (for **1**-*E*) or from 2.5 to −0.2 nm (for **1**-*Z*) at 0.05 nm intervals, followed by solvation and optimization (gradient 3.0 kcal/molÅ), were analyzed.^8^ The local minima binding energy (MBE) structures were optimized once again (gradient 0.5 kcal/molÅ) and used as the starting conformations for the 2.0 ns MD simulations.

Figure 12C,D depicts the MM results of the total binding energies as well as the electrostatics and van der Waals contributions obtained for **1**-*E*:βCyD and **1**-*Z*:βCyD inclusion complex formation as a function of the distance between the centers of mass of the interacting partners in Figure 12A,B. The latter represents the main term of the binding energy, although electrostatic interactions are not negligible. The optimized MBE structures are shown in Figure 12E,F. The 2 ns MD simulations in the presence of water showed that the complexes remained stable (Figure 12G,H), the final structures being close to that of the initial MBE structure. The average angles between the main axis of the macroring and the S_0_ → S_1_ (S_0_ → S_2_) transition moments were 12.5 ± 6.5° (12.1 ± 6.5°) for the **1**-*E*:βCyD complex and 14.4 ± 6.6° (16.8 ± 6.1°) for the **1**-*Z*:βCyD complex (Supporting Information, Figure S34), which agree with the positive sign of both bands for the π → π* (S_0_ → S_2_) and n → π* (S_0_ → S_1_) transitions observed in the respective ICD spectra. MD data obtained for other initial orientations of the guest and host partners (Supporting Information, Figure S33) that showed the azo moieties penetrating inside the βCyD cavity were also consistent with the experimental ICD bands sign. However, some approaches involving **1**-*Z* either dissociated during the MD trajectories (*Z*-polar primary face) or showed the azobenzene part significantly outside the βCyD cavity (*Z*-polar secondary face); in these cases, the average angles did not reproduce the signs of transition bands (Supporting Information, Figure S35).

#### Formation of Dimers of the βCyD-Azobenzene Derivative **2**

**Figure 13:**
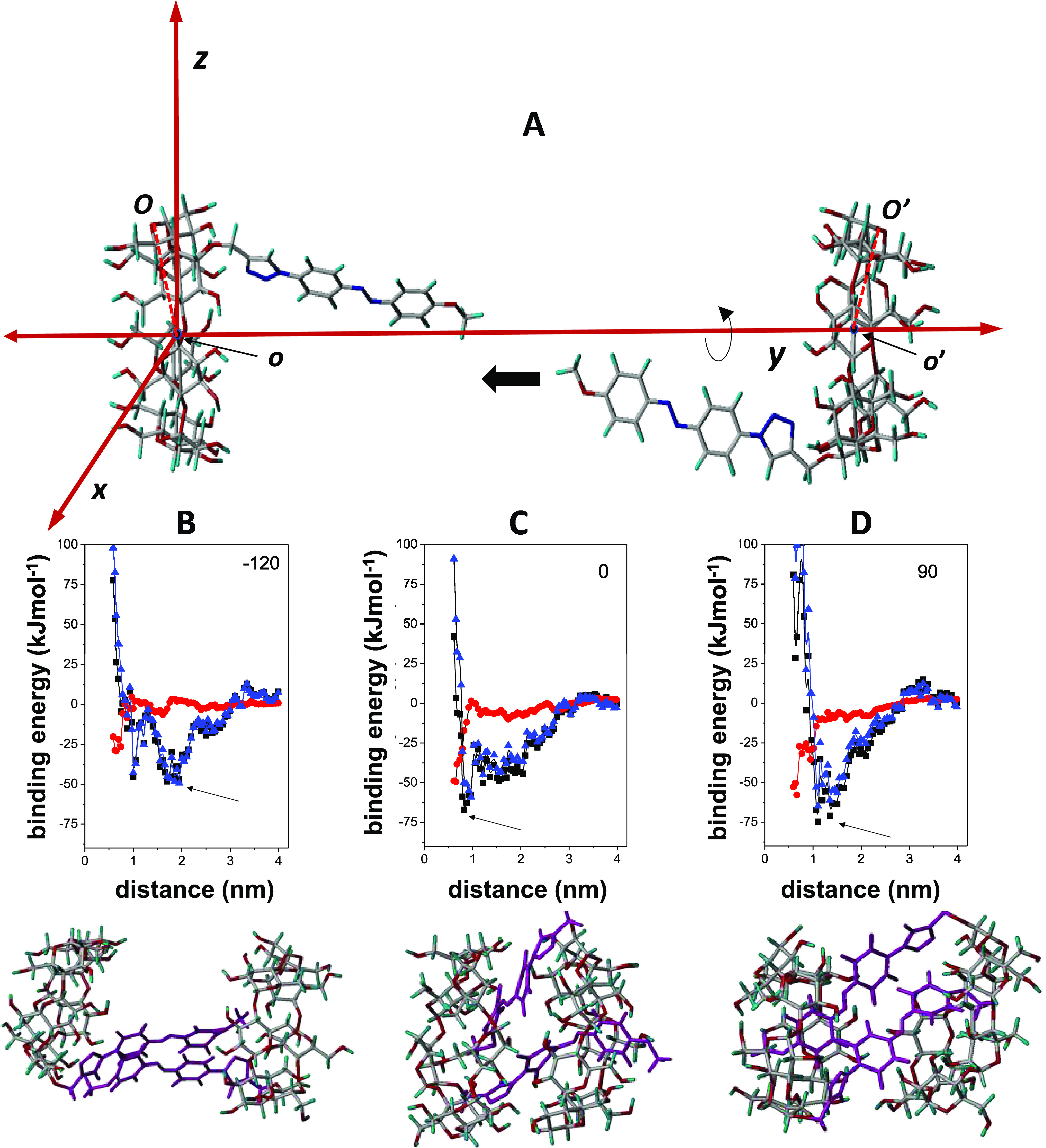
(A) Coordinate system used for the **2**-*E* head-to-head approaching by the secondary face along the *y* coordinate. (B), (C) and (D) Total binding energies (black squares), electrostatics (red circles), and van der Waals (blue triangles) contributions as a function of the **2**–**2′** distance along the *y* coordinate (*o*–*o*′) for the **2**-to-**2′** approaching with −120, 0, and 90° dihedral angles, respectively; the optimal MBE structures, indicated by the arrows, are also shown.

The protocol described above, which effectively replicates the experimental findings on the supramolecular interaction between the model compound **1** and βCyD, was subsequently employed to investigate the supramolecular dimer formation ability and stability of the βCyD derivatives bearing the azobenzene module at a secondary position. To minimize the computational cost, the nonmethylated compound **2** was chosen for this purpose. First, an MM optimized structure of **2** in the *E*-configuration was positioned with the center of mass of the macrocycle (*o*) at the origin of a coordinate system and the extended triazolylazobenzene moiety oriented toward the *y* axis positive direction. Then, an identical **2′**-*E* derivative was placed in a similar manner but with its azo-group in the opposite direction, at distances where it hardly interacted with the first **2**-*E* derivative (Figure 13A). The most favorable orientations for dimer formation were scanned by the complete rotation of **2′**-*E* around the *y* axis, namely, changing the dihedral *O-o-o*′*-O*′ angle from −180 to 180°, while simultaneously approaching it to **2**-*E* at small intervals and obtaining the binding energy for each structure in the vacuum. They corresponded to −120, 0, and 90° dihedral angles. For each of these orientations, **2′**-*E* was approached to **2**-*E* in 0.05 nm steps along the *y* coordinate from *y* = +4 to 0.5 nm in the presence of explicit water. Briefly, the first structure generated with **2′**-*E* at *y* = +4 nm was solvated and optimized (gradient 3 kcal/molÅ); the solvent was then removed and the **2′**–**2** distance was decreased by 0.05 nm, and an iterative process analogous to that described above for the **1**-*E*:βCyD complex formation was applied.

As expected, the larger contribution to the binding energy came from van der Waals contributions, which, at the shorter distances, became strongly repulsive due to unfavorable steric contacts (Figure 13B–D). For the −120° dimer, the planar *E*-configured azo moieties in the MBE structure adopted a sandwich-type structure and were located between the two CyD macrorings, neither of them penetrating inside the neighboring cavities. In the 0 and 90° MBE dimers, however, the attached azo moieties slightly penetrated the neighboring cavities. These structures were optimized once again (0.5 kcal/molÅ) and used for 1 ns MD simulations. For the three systems, the interaction energies remained favorable during the whole trajectories, especially for the 90° structure. At the end point, the *E*-azobenzene moieties were positioned between the neighboring macrorings, with limited penetration into the cavity and in close proximity to each other. This arrangement facilitated favorable interactions while preventing clashes between the macrorings (Supporting Information, Figures S36–S38). The histories for the angles between the S_0_ → S_1_ transition dipole moments and the main axis of the βCyD rings for the 90° structure provided average values of 56.4 ± 5.3° and 26.3 ± 11.8°, in agreement with a negative weak signal in the ICD spectra for the low energy band (n → π*). Note that, according to the theory, the intensity would be zero for an angle of 54.7° and would become negative for smaller angle values when the azobenzene group is located outside the CyD cavity (Supporting Information, Figure S36). Notably, the proximity of the azobenzene groups in the dimers would also agree with the presence of the excitonic coupling signal observed in the most intense π → π* transition region of the ICD spectra of **2**-*E* and **3**-*E* aqueous solutions.

To simulate the effect of *E*-to-*Z* photoisomerization, the azobenzene moieties in the MBE structures of the **2**-*E* dimer depicted in Figure 13 were changed to the *Z*-configuration, optimized (MM), and the resulting **2**-*Z* dimer MBE structures used in 1 ns MD simulations. The 90° and −120° (Supporting Information, Figures S36 and S37) dimer structures dissociated before the end of the MD trajectories (after approx. 0.5 and 0.9 ns respectively). The 0° structure remained stable (Supporting Information, Figure S38) but dissociated after 1.5 ns (data not shown). Altogether, the computational data emulate dimer dissociation upon irradiation of the **2**-*E* dimer aqueous solution at 365 nm.

## Conclusions

Following our combined efforts in the development of self-assembling cyclooligosaccharides^18^ and photoresponsive carbohydrate derivatives,^40^ a new prototype combining both functionalities is here presented: secondary face-linked βCyD-azobenzene derivatives. The synthesis was achieved efficiently using the click CuAAC reaction, known for its compatibility with selective hydroxyl elaboration for specific applications. Through comprehensive spectroscopic (UV–vis, ICD, NMR) and computational (MM, MD) investigations, we have explored the photoswitching, supramolecular, and structural properties of these derivatives.

Control experiments have demonstrated that the generated triazoylazobenzene motif can enter the βCyD cavity in both *E*- and *Z*-configurations, forming 1:1 inclusion complexes with similar association constants. However, when covalently attached to a single *O*-2 position of βCyD, self-inclusion is prevented due to the rigidity imposed by the triazole tether. Furthermore, the capability of the *E*-configured triazoylazobenzene module to enter the cavity of a neighboring βCyD and form heterodimers is significantly reduced.

Remarkably, the *E*-isomer self-assembles in aqueous media, forming stable face-to-face homodimers, while the *Z*-isomer remains in a monomeric form. The homodimer species is stabilized by synergistic aromatic–aromatic and aromatic–cavity interactions and demonstrates resistance to competition with a third guest species, even in medium polarity environments (up to 75% methanol–water mixtures). Encouragingly, *E*-to-*Z* photoisomerization completely disrupts the supramolecular assemblies.

The significance of this work in the field of stimuli-responsive cyclooligosaccharide-based supramolecular materials is threefold. First, it offers a straightforward strategy to introduce self-organization abilities that can be controlled by light while maintaining full diastereomeric purity. Second, it provides insights into suitable techniques for monitoring the system in the presence of competing species. Third, it lays the foundation for designing more sophisticated architectures with hierarchical self-assembling behaviors, such as coupling homodimer formation with co-assembling processes. Currently, we are exploring the application of these concepts in the development of light-sensitive molecular containers in our laboratories.

## Data Availability

The data underlying this study are available in the published article and its Supporting Information.

## Supporting Information Available

Synthetic methods; spectroscopic UV–vis, fluorescence, and induced circular dichroism methods; NMR and MS spectra; additional UV–Vis and ICD spectra; full titration data; and computational methods (PDF)
