# Supplementary material for: Reversible Light-Induced Dimerization of Secondary Face Azobenzene-Functionalized β-Cyclodextrin Derivatives
Source: J Org Chem. 2023 Jun 21;88(13):8674–89. doi: 10.1021/acs.joc.3c00564 (PMC10337044; doi:10.1021/acs.joc.3c00564)
Supplement: Supplementary file 1 — jo3c00564_si_001.pdf [file jo3c00564_si_001.pdf]

SUPPORTING INFORMATION

Reversible light-induced dimerization of secondary face azobenzene-functionalized β-cyclodextrin derivatives

Gonzalo Rivero-Barbarroja,[a§] Carlos Fernández-Clavero,[b§] Cristina García-Iriepa,[b] Gema Marcelo,[b] M. Carmen Padilla-Pérez,[a] Tania Neva,[c] Juan M. Benito,[c] Stéphane Maisonneuve,[d] Carmen Ortiz Mellet,[a]* Juan Xie,[d]* José M. García Fernández[c]* and Francisco Mendicuti[b]*

[a] Department of Organic Chemistry, Faculty of Chemistry, University of Seville, 41012 Sevilla, Spain

[b] Universidad de Alcalá, Departamento de Química Analítica, Química Física e Ingeniería Química and Instituto de Investigación Química "Andrés del Rio", 28805, Alcalá de Henares, Madrid, Spain

[c] Instituto de Investigaciones Químicas (IIQ), CSIC – Universidad de Sevilla, Américo Vespucio 49, 41092 Sevilla, Spain

[d] ENS Paris-Saclay, CNRS, Photophysique et Photochimie Supramoléculaires et Macromoléculaires, Université Paris-Saclay, Gif-sur-Yvette 91190, France

[§] These authors contributed equally.

E-mail Addresses

mellet@us.es; joanne.xie@ens-paris-saclay.fr; jogarcia@iiq.csic.es; francisco.mendicuti@uah.es

TABLE OF CONTENTS

1. **General Methods** S3
2. **Spectroscopic Measurements** S3
3. **Thermodynamics of Complexation** S4
4. **Photoisomerization and Thermal Isomerization Studies for Compounds 2 and 3. Irradiation Conditions** S5
5. **General Procedure for the Determination of the Photoconversion Yields by Absorption and ^{1}H NMR** S5
6. **General Procedure for the Determination the Molar Absorption Coefficient (ε)** S6
7. **General Procedure for the Determination of the Half-Life** S6
8. **General Procedure for the Fatigue Resistance Measurements** S7
9. **Fluorescence Spectroscopy Studies** S7
10. **Theoretical Studies. $S_0 \rightarrow S_1$ and $S_0 \rightarrow S_2$ Transition Moments Orientation** S8
11. **Molecular Mechanics and Molecular Dynamics Protocols** S8
12. **Detailed Synthetic Procedures** S10
13. **NMR (Freshly Prepared Solutions – *E*-Isomer) and MS Spectra of the New Compounds (Figure 1S-4S)** S14
14. **1-*E* / 1-*Z* Photoconversion (Figure 5S and 6S)** S18
15. **Photoisomerization and Thermal Isomerization Studies (Figure S7)** S19
16. **Determination of the Half-Life (Figure S8)** S19
17. **Fatigue Resistance Measurements (Figure S9)** S20
18. **Fluorescence Spectroscopy Studies (Figure S10)** S21
19. **UV-Vis and Induced Circular Dichroism (ICD) Studies (Figure S11-S21)** S21
20. **NMR Studies (Figure S22-S31)** S27
21. **Theoretical Studies (Figure S32-S38)** S36

1. General Methods. Reagents and solvents were purchased from commercial sources and used without further purification. Optical rotations were measured using a sodium lamp (λ= 589 nm) at 25 ºC in 1 dm tubes. NMR experiments were performed on Bruker 400 (400 and 100.6 MHz), Bruker Ascend 400/R (400 and 100.6 MHz), Bruker Ascend 500 (500 and 125.7 MHz) and Bruker Ascend 600 (600 and 150.8 MHz) spectrometers. 2D COSY (Correlated Spectroscopy), 1D and 2D TOCSY (Totally Correlated Spectroscopy), HMQC (Heteronuclear Multiple-Quantum Coherence Spectroscopy), HSQC (Heteronuclear Single-Quantum Coherence Spectroscopy), HMBC (Heteronuclear Multiple-Bond Correlation), 2D ROESY (Rotating-frame Overhauser Effect Spectroscopy) and 2D NOESY (Nuclear Overhauser Spectroscopy) experiments were employed to assist NMR assignments. CD_3OD and $(CD_3)_2SO$ have been used as solvents. Chemical shifts (δ) are reported in parts per million (ppm) relative to the residual solvent peak. The values of the coupling constants (*J*) are measured in Hz. The abbreviations to indicate the multiplicity of the signals are: s (singlet), d (doublet), t (triplet), and m (multiplet). The abbreviation "b" indicates that the signal is "broad". For ESI mass spectra, 0.1 µM sample concentrations were used, the mobile phase consisting of 50% aq MeOH at 0.1 $mL{\cdot}min^{-1}$. Thin-layer chromatography (TLC) was performed on aluminum sheets coated with Silica Gel 60 F254 Merck, with visualization by UV light (254 nm) and by charring with 10% H_2SO_4 in ethanol or ninhydrin 0.1% in ethanol and heating at 100 ºC. Column chromatography was performed on Silicagel 60 (E. Merck, Geduran® Si 60, 40-63 µm). Deionized water (Milli-Q) was employed for all aqueous solutions. Elemental analyses were performed at the Servicio de Microanálisis of the Instituto de Investigaciones Químicas, using an elemental analyser Leco TruSpec CHN.

2. Spectroscopic Measurements. Absorption spectra were monitored by using UV–Vis Uvikon 941 (Kontron Instruments), V-630 (Jasco), Cary-100, Cary-4000 or Cary-5000 (Agilent Technologies) spectrophotometers. Spectra were recorded with a scan speed 100 nm/min (Uvikon 941, Cary-100, Cary-4000, Cary-5000) and 200 nm/min (V-630) and 1 nm resolution.

Unless otherwise stated, a pair of Hellma® or Starna Scientific quartz (23/Q/10) cells of 10 mm path length have been used for solution measurements. Response of 0.5 s.

Steady-state fluorescence measurements were carried out by using a PTI Quanta Master spectrofluorimeter equipped with a Xenon flash lamp as a light source, with single concave grating monochromators and Glan-Thompson polarizers in the excitation and emission paths. The detector was a photomultiplier cooled by a Peltier system. Polarizers were fixed at the "magic angle" condition. Corrections of the inner effect were made by Equation S1[1]

$$I_{corr} = I_{obs}\ 10^{\left(\frac{A_{ex}+A_{em}}{2}\right)} \qquad \text{(S1)}$$

where A_{ex} and A_{em} are the absorption at the wavelength of excitation and emission respectively.

Circular dichroism measurements were performed using a JASCO 715 spectropolarimeter. The recorded spectra were the average of two scans taken at a speed of 200 nm/min with a time-response of 0.5 s. The sensitivity and resolution were fixed at 0.1 deg and 0.5 nm respectively. Measurements were performed in a 2 mm (10 mm) path quartz cell when monitoring the spectra of **2** and **3** (**1**). The samples were irradiated in their own quartz cuvettes (2 mm or 10 mm) by using the LZC-4Xb photoreactor distributed by Luzchem which is equipped with 4 LED tubes of 8 W each of blue light, whose maximum emission wavelength is centered at 456 nm. In case of irradiation at 365 nm (or 254 nm) a Desaga (Heidelberg) light emitting HP-UVIS dispositive containing (8 W) tubes which produce radiation at these two wavelengths was used.

3. Thermodynamics of Complexation. For the 1:1 complexation of a guest molecule (G) with a CyD host molecule, whose association is described by the 1:1 equilibrium G + CyD ⇆ GCyD, the association constant *K*, is given by Equation S2

$$\mathrm{K} = \frac{[\mathrm{G\,CyD}]}{[\mathrm{G}][\mathrm{CyD}]} \qquad \text{(S2)}$$

where [G], [CyD], and [GCyD] are the concentration of each species, guest, CyD and complex at the equilibrium.

By measuring a spectroscopic magnitude X whose intensity is sensitive to the guest complexation, it can be related to the initial X_0 and X_∞ values for the guest in the absence of CyD (at $[CyD]_0 = 0$) and for the total complexed guest (at $[CyD]_{0\to\infty}$) and the *K* values by the well-known Equation S2[2]

$$\mathrm{X} = \frac{\mathrm{X_0} + \mathrm{X_\infty K[CyD]_0}}{1 + \mathrm{K[CyD]_0}} \qquad \text{(S3)}$$

that can be reorganized as Equation S4

$$\frac{[\mathrm{CyD}]_0}{(\mathrm{X} - \mathrm{X_0})} = \frac{1}{(\mathrm{X_\infty} - \mathrm{X_0})K} + \frac{[\mathrm{CyD}]_0}{(\mathrm{X_\infty} - \mathrm{X_0})} \qquad \text{(S4)}$$

Both Equations S3 and S4 provide association constants of the 1:1 [G:CyD] complex from the so-called non-linear and linear fits, respectively, and under the assumption that the [CyD] is in excess. Then, the concentration of complexed CyD is $[G{:}CyD] \ll [CyD]_0$ and the free [CyD] is almost the same as $[CyD]_0$ at any cyclodextrin concentration.

4. Photoisomerization and Thermal Isomerization Studies for Compounds 2 and 3. Irradiation Conditions. Photochromic reactions (ENS Paris-Saclay) were induced *in situ* by continuous irradiation with a Hg/Xe lamp (Hamamatsu, LC6- or LC8-Lightningcure, 200 W) equipped with narrow band interference filters of appropriate wavelengths Semrock FF01-340/12-25 for λ_{irr} = 340 nm, Semrock FF01-370/10-25 for λ_{irr} = 365 nm; Semrock FF01-543/22-25 for λ_{irr} = 543 nm. The incident lamp power was measured by means of an Ophir PD300-UV photodiode. NIR contribution (P_{LP}) has been measured and subtracted from the total value using Schott long pass filters LP-545 and LP-595 nm, respectively for irradiations at 365 nm and 543 nm that is let through the Semrock filter (P_{Total}), and considering a 90% transmittance: $P_{\lambda irr} = P_{Total} – (10/9 \times P_{LP})$.

5. General Procedure for the Determination of the Photoconversion Yields by Absorption and ^{1}H NMR. ^{1}H-NMR spectra were recorded on a JEOL ECS-400 spectrometer (399.78 MHz for ^{1}H) equipped with an autotunable broad band probe. A freshly prepared solution of *E*-azobenzene derivative was prepared in a D_2O with an absorbance range of 0.2 to 4, which corresponds to an acceptable accuracy for the limit of absorbance of the spectrophotometer at the maximum wavelength of the compound, and also, to a sufficient concentration for NMR measurements. The deuterated solution was monitored by UV-Vis absorption and ^{1}H NMR before and after irradiation. More precisely, the absorbance of solution was firstly recorded, and a small amount of the solution was taken for the ^{1}H NMR monitoring (see Figure S7). This sequence was then repeated after irradiation of the solution to the photostationary state (PSS). The ^{1}H NMR spectra were recorded with a relaxation delay of 2 s, and 512 or 1024 scans. Then, the corresponding photoconversion yield (CY) was calculated, after integration of the signal areas, according to the following Equation S5,

$$CY_i = \frac{A_{\boldsymbol{E}}}{A_{\boldsymbol{E}} + A_{\boldsymbol{Z}}} \quad (S5)$$

where,

CY_i, represents the conversion yield for a given state (i) of the solution,
$A_{\boldsymbol{E}}$, represents the area taken under the signal of *E*-isomer,
$A_{\boldsymbol{Z}}$, represents the area taken under the signal of *Z*-isomer.

6. General Procedure for the Determination the Molar Absorption Coefficient (ε). A mother solution of the tested compound was freshly prepared in water in a precise concentration. From the mother solution, a series of 7 to 12 daughter solutions were prepared with a concentration range from 5 µM to 50 µM, and their absorbance was measured independently by UV-Vis

absorption at room temperature. To ensure the reproducibility and evaluate the accuracy of the measurements, each recorded spectrum was divided by its concentration in order to obtain the corresponding normalized spectrum; only the overlapped normalized spectra are used for the determination of the molar absorption coefficient. Then, the absorption was taken at the maximum of wavelength and at the isosbestic point of each original spectrum to calculate the molar absorption coefficients by a linear regression fitting using the least squares method and with a forcing to pass through the origin of coordinates. The molar absorption coefficient determined at the isosbestic point was then used for the further normalizations, which let us avoid the consideration *E*/*Z* proportion in the solution.

The molar absorption coefficient is calculated according to the Beer-Lambert's law (Equation S6),

$$A(\lambda) = \varepsilon(\lambda) \cdot l \cdot C \quad (S6)$$

where

$A(\lambda)$, represents the absorbance at the wavelength λ
$\varepsilon(\lambda)$, represents the molar absorption coefficient at the wavelength λ, in $mol.L^{-1}.cm^{-1}$
l, is the distance of the trajectory in the media, in cm
C, is the concentration of the substrate in $mol \cdot L^{-1}$

7. General Procedure for the Determination of the Half-Life. For determination of the half-life of azobenzene derivatives, the thermal *Z*→*E* isomerization was tracked by measuring UV-Vis absorption spectra at about 37±1 °C. The obtained time-dependent absorption profiles, taking *Z*-**2** as an example (black cross, Figure S8), were fitted with the following first order rate Equation S7:

$$Abs(t) = A_o - (A_o - A_i)e^{-kt} \quad (S7)$$

where

A_o, is the absorption of original *E*-isomer
A_i, is the absorption at the PSS_{365}
k, is the rate constant of thermal isomerization
t, is the time in minutes
After fitting (Figure S8, red curve), the rate constant k_{310} at 37 °C (T = 310 K) was obtained from the fitting parameters, and the half-life was then calculated according to Equation S8:

$$t_{1/2} = \frac{\ln(2)}{k_T} \quad (S8)$$

8. General Procedure for the Fatigue Resistance Measurements. Starting from a solution of compound containing 100% of the *E*-isomer form, the solution was irradiated sequentially at 365 nm and at 543 nm. The absorbance was measured before and after each illumination, realized with constant optical length distance and power. The sequence, which constitutes a cycle, was repeated for a minimum of 10 times (see Figure S9).

9. Fluorescence Spectroscopy Studies. Fluorescence is detected for the model compound **1** (fluorescence quantum yield $\Phi \approx 7\times10^{-5}$ using quinine sulfate in 0.1 M H_2SO_4 as a standard)[3] as well as for the βCyD-azobenzene hybrids **2** and **3** in aqueous solutions, either before or after irradiation at 365 nm (major *E* or *Z*-isomer, respectively). Compound **1** showed a main band in the fluorescence spectrum centered at 475 nm and a shoulder at 435 nm, whereas the cyclodextrin derivatives **2** and **3** exhibited an additional band at around 525 nm (Figure S10). The fluorescence emission intensities, corrected for the inner filter effect due to the large absorbance,[4] experienced a linear increase with concentration in the case of **1** but not for **2** or **3**, although they followed a similar trend at the three wavelengths. We were interested at scrutinizing the variations in the ratio between the intensities of the red band located at 525 nm and the band at 435 nm, since a significant enhancement of this ratio would denote the presence of intermolecular excimers resulting from aggregation. Regrettably, although a slight increase was observed, data did not reach significance, changes in the intensity ratio remained negligible upon modifying the excitation wavelength from 295 nm to 335 nm at 5 nm intervals. The low intensity of fluorescence, in the same order as the intensity of the Raman band of water used as solvent, also prevented using lifetime or fluorescence polarization measurements to further investigate potential self-assembling behaviors.

10. Theoretical Studies. $S_0 \rightarrow S_1$ and $S_0 \rightarrow S_2$ Transition Moments Orientation. The excited state transition electric dipole moments were calculated for both the model compound **1** and the βCyD-azobenzene hybrids **2** and **3** derivatives following procedures described previously. $S_0 \rightarrow S_1$ ($n \rightarrow \pi^*$) and $S_0 \rightarrow S_2$ ($\pi \rightarrow \pi^*$) in the *E*-isomer are usually considered collinear.[5,6] Our calculations revealed that the angle between transition moments for the *E*-isomer of **1** and **2** were 1.2 and 2.4 deg respectively. For the *Z*-isomer the angles were slightly higher, 15.5 and 14.7 deg respectively. The presence of the βCyD macroring did not significantly change the orientation of the dipoles

or the angle between them. Permethylation of the βCyD module in **3** did also not significantly influence the orientation of transition dipoles moments (see Figure S32).

11. Molecular Mechanics and Molecular Dynamics Protocols. Molecular Mechanics (MM) and Molecular Dynamics (MD) calculations were performed with Sybyl X-2.0 and the Tripos Force Field.[7,8] A relative permittivity ε of 1 (3.5) was used in presence of explicit water (vacuo). Charges for compounds **1-3** were obtained by using the Gaussian suite of quantum chemical programs[9] at the HF/6-31G(d) level of theory as described later. Optimizations were carried out by the simplex algorithm, and the conjugate gradient was used as a termination method with a gradient of 3.0 Kcal/molÅ (0.2 Kcal/molÅ) for the MM calculations in water (vacuum).[10,11] Non-bonded cut-off distances were set at 12 Å. The Molecular Silverware (MS) algorithm[12] with periodic boundary conditions (PBC) was employed for simulations in the presence of water (cubic box sizes and number of explicit water molecules are included in the legends of figures S34-S38). MD simulations were performed on the MM most favorable structures, which were optimized again (gradient of 0.5 Kcal/molÅ). During MD the bonds where H atoms were involved were constrained from vibrating, but the rest of the conformational parameters were variable. Trajectories were performed starting from 1 K, and the temperature was increased by 20 K intervals equilibrating the system at each intermediate temperature for 500 fs up to reaching the temperature of interest of 300 K. Once at this temperature, an additional equilibration period up to 25 ps was used. The whole heating/equilibration period was discarded from the analysis. From this point on, the rest of the trajectory time (ns) was simulated at 2 fs integration time steps. The velocities were rescaled at 10 fs intervals. Structures obtained from the analysis of MD trajectories were saved every 500 fs, yielding *n* images [n=time (ns)$\times 10^6$/500] for subsequent analysis.

The excited state transition electric dipole moments were computed for the *E*- and the *Z*-isomers of both, **1** and **3**. Calculations in this case were performed implicitly considering water as the solvent by using the polarizable continuum model (PCM).[13] First, the structure of these four systems was optimized in the ground state at the DFT level, using the B3LYP functional and the 6-31+G (d,p) basis set.[14,15] Their minimum character was ensured by frequency calculations. Then, TD-DFT calculations were performed on top of these optimized geometries, using the CAM-B3LYP functional[16] (keeping the same basis set) to compute the aimed excited state transition electric dipole moment. Calculations were performed with the Gaussian 16 suite of programs (see Figure S33-S35).

12. Detailed Synthetic Procedures

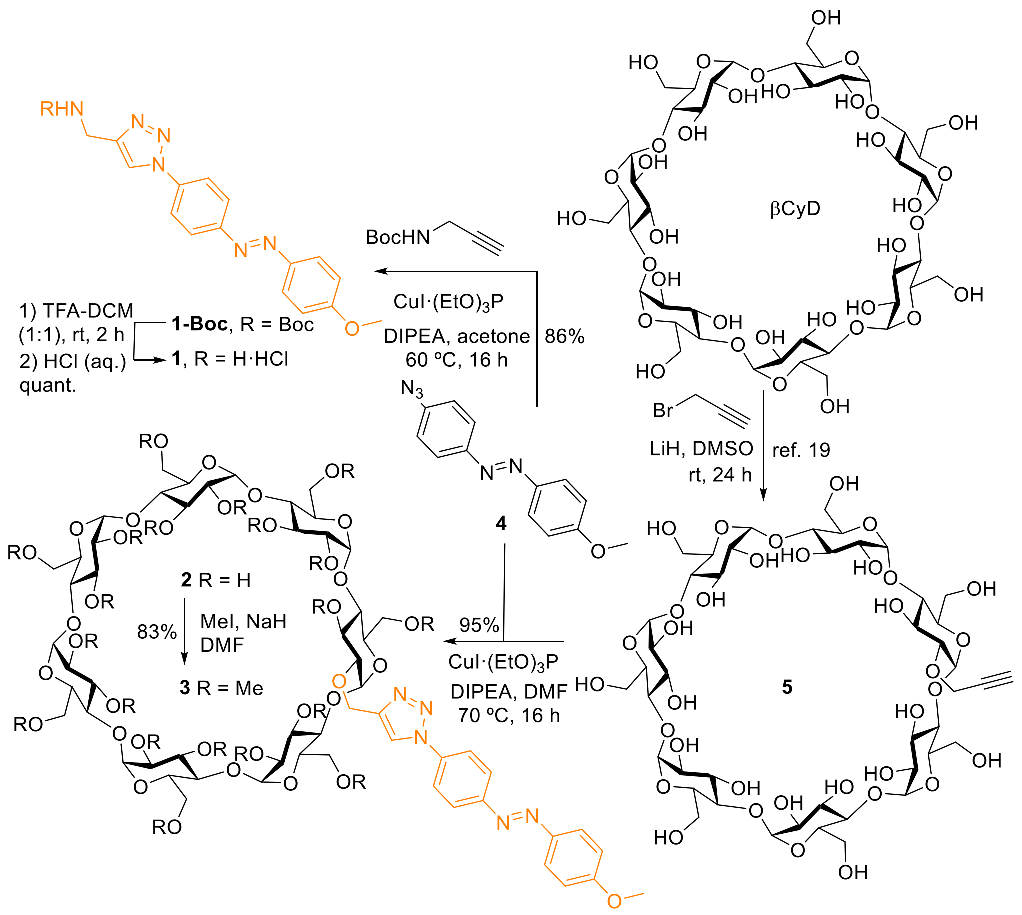

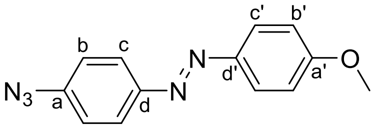

1-(4-Azidophenyl)-2-(4-methoxyphenyl)diazene (4). To a solution of 1-(4-azidophenyl)-2-(4-hydroxyphenyl)diazene[17] (770 mg, 3.22 mmol) in DMF (55 mL), K_2CO_3 (1.11 g, 8.05 mmol, 2.5 eq) was added. The mixture was stirred 10 min. Then, MeI (250 µL, 4.03 mmol, 1.25 eq.) was added and the suspension was heated at 65 ºC (digital aluminum dry block heater) for 5 h. The mixture was cooled, diluted with EtOAc (25 mL) and the aqueous phase was extracted with EtOAc (3 × 15 mL). The combined organic phase was washed with brine (25 mL), dried ($MgSO_4$), filtered and concentrated. The crude product was purified by column chromatography (1:20 → 1:5 → 1:2 EtOAc-Hex). Yield 801 mg (98%). ^{1}H NMR (400 MHz, $CDCl_3$): 7.95-7.90 (m, 4 H, H-c, H-c'), 7.17 (d, 2 H, J = 8.9 Hz, H-b), 7.04 (d, 2 H, J = 8.8 Hz, H-b'), 3.92 (s, 3 H, CH_3O-~~Azo~~). The spectroscopic data were accorded with those previously reported.[18]

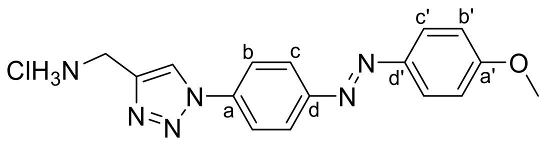

1

4-(4-Aminomethyl-1*H*-1,2,3-triazol-1-yl)-4'-methoxyazobenzene (1). To a solution of 1-(4-azidophenyl)-2-(4-methoxyphenyl)diazene (**4**, 81 mg, 0.32 mmol) in acetone (16 mL), *N*-Boc-propargylamine (60 mg, 0.38 mmol, 1.2 eq), DIPEA (55 µL, 0.32 mmol, 1.0 eq.) and CuI·$(EtO)_3P$ (5.7 mg, 0.016 mmol, 0.05 eq.) were added. The mixture was stirred at 60 ºC (digital aluminum dry block heater) for 16 h. Then, the solvent was evaporated and purified by column chromatography (1:2 EtOAc-Hex) to give 4'-methoxy-[4-(4-*tert*-butoxycarbonylaminomethyl)-1*H*-1,2,3-triazol-1-yl]-azobenzene (**1**-Boc). Yield 112 mg (86%). R_f 0.31 (1:2 EtOAc-Hex). ^{1}H NMR (400 MHz, $CDCl_3$): δ 8.05 (m, 3 H, H-c, CH-triazole), 7.97 (d, 2 H, *J* = 8.9 Hz, H-c'), 7.89 (d, 2 H, *J* = 8.9 Hz, H-b), 7.06 (d, 2 H, H-b'), 5.18 (bs, 1 H, NH), 4.52 (d, 2 H, CH_2N, *J* = 5.9 Hz), 3.93 (s, 3 H, OMe), 1.49 (s, 9 H, $(CH_3)_3$). ^{13}C NMR (100.6 MHz, $CDCl_3$): δ 162.6, 152.3, 146.9 (C-a, C-a', C-d, C-d', CO, C-triazole), 125.1 (C-c'), 124.0 (C-c, CH-triazole), 120.9 (C-b), 114.3 (C-b'), 79.9 (*C*$(CH_3)_3$), 55.6 (O*Me*), 29.7 (*C*H_2N), 28.4 (C(*C*$H_3)_3$). ESI-MS: *m/z* 409.33 [M + H]$^+$. Anal. Calcd. for $C_{21}H_{24}N_6O_3$: C 61.75, H 5.92, N 20.58. Found: C 61.83, H 6.07, N 20.64.

The above Boc-protected derivative (105 mg, 0.26 mmol, 1 eq) was dissolved in TFA:DCM (1:1, 6 mL) and stirred at rt for 1 h. The solvent was removed under reduced pressure and coevaporated with DCM (5 × 10 mL). The residue was dissolved in aq. HCl (0.1 M) and freeze-dried to obtain **1** as the corresponding hydrochloride. Yield 89.6 mg (quant). R_f 0.26 (60:10:1 DCM-MeOH-H_2O). ^{1}H NMR (600 MHz, $(CD_3)_2SO$): δ 8.96 (bs, 1 H, CH triazole), 8.47 (bs, 3 H, NH_3), 8.13 (d, 2 H, *J* = 9.1 Hz, H-b), 8.09 (d, 2 H, *J* = 9.0 Hz, H-c), 7.96 (d, 2 H, *J* = 9.0 Hz, H-c'), 7.18 (d, 2 H, *J* = 8.9 Hz, H-b'), 4.25 (s, 2 H, CH_2N), 3.89 (s, 3 H, CH_3O). ^{13}C NMR (150.9 MHz, $(CD_3)_2SO$): δ 162.9, 152.1, 146.6, 137.9 (C-a, C-a', C-d, C-d', C-triazole), 125.4 (C-c'), 124.4 (C-b), 123.3 (CH-triazole), 121.4 (C-c), 115.2 (C-b'), 56.2 (CH_3O), 34.3 (CH_2N). ESI-MS: *m/z* 309.08 [M + H]$^+$. Anal. Calcd. for $C_{16}H_{17}ClN_6O$: C 55.74, H 4.97, N 24.37. Found: C 55.61, H 4.85, N 24.26.

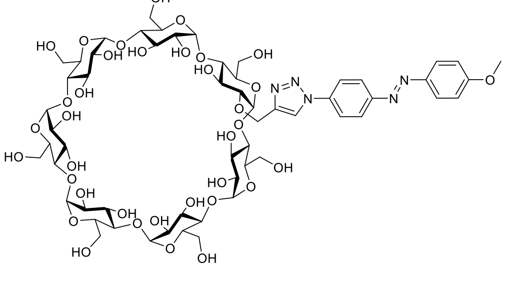

(*E*)-2^I-*O*-{[1-(4-((4-Methoxyphenyl)diazenyl)phenyl)-1*H*-1,2,3-triazol-4-yl]methyl}-cyclomaltoheptaose (2). To a solution of 2^{I}-*O*-propargylcyclomaltoheptaose[19] (**5**, 1.50 g, 1.28 mmol) in DMF (125 mL), 1-(4-azidophenyl)-2-(4-methoxyphenyl)diazene (**4**, 388 mg, 1.53 mmol, 1.2 eq), DIPEA (275 µl, 1.66 mmol, 1.3 eq.) and CuI·$(EtO)_3P$ (23 mg, 0.064 mmol, 0.05 eq.) were added. The mixture was stirred at 70 ºC (digital aluminum dry block heater) for 16 h. Then, the mixture was poured into acetone (1 L) and the precipitate was collected by filtration to give **2**. Yield 1.72 g (95%). R_f 0.66 (6:3:1 MeCN-H_2O-NH_4OH). $[\alpha]_D$ = +120.3 (c 1.0, DMSO). ^{1}H NMR (600 MHz, CD_3OD): δ 8.70 (s, 1 H, C*H*-triazole), 8.11-8.06 (m, 4 H, H-b, H-c), 7.98 (2 H, *J* = 8.9 Hz, H-c'), 7.12 (2 H, *J* = 8.9 Hz, H-b'), 5.12 (d, 1 H, $^2J_{H,H}$ = 12.2 Hz, CH_2a-Azo), 5.11 (d, 1 H, $J_{1,2}$ = 3.5 Hz, H-1^{I}), 5.12 (d, 1 H, CH_2b-Azo), 5.02 (d, 1 H, $J_{1,2}$ = 3.3 Hz, H-1^{II}), 4.99-4.95 (m, 5 H, H-$1^{III-VII}$), 4.09 (t, 1 H, $J_{2,3}$ = $J_{3,4}$ = 9.3 Hz, H-3^{I}), 3.93 (s, 3 H, CH_3O-Azo), 3.90 (t, 1 H, $J_{2,3}$ = $J_{3,4}$ = 9.3 Hz, H-3^{II}), 3.89 (dd, 1 H, $J_{5,6a}$ = 4.4 Hz, $J_{6a,6b}$ = 13.1 Hz, H-$6a^{I}$), 3.85 (bd, 1 H, H-$6b^{I}$), 3.84 (m, 2 H, H-$6a^{II}$), 3.88-3.80 (m, 15 H, H-$3^{III-VII}$, H-$6a^{III-VII}$), 3.77 (m, 1 H, H-5^{II}), 3.75 (1 H, $J_{4,5}$ = 9.5 Hz, H-5^{I}), 3.77-3.72 (m, 5 H, H-$5^{III-VII}$), 3.65 (dd, 1 H, H-2^{I}), 3.61 (t, 1 H, $J_{4,5}$ = 9.3 Hz, H-4^{I}), 3.54 (dd, 1 H, H-2^{II}), 3.52 (dd, 1 H, H-4^{II}), 3.53-3.47 (m, 10 H, H-$2^{III-VII}$, H-$4^{III-VII}$). ^{13}C NMR (150.9 MHz, CD_3OD): δ 162.9, 152.4, 146.8, 145.2, 137.9 (C-a, C-d, C-a', C-d', C-triazole), 124.7 (C-c'), 123.6 (C-b), 122.4 (CH-triazole), 120.8 (C-c), 114.1 (C-b'), 102.6, 102.5, 102.4, 102.2 (C-1^{II-VII}), 100.7 (C-1^{I}), 82.2 (C-4^{II}), 81.6, 81.5 (C-$4^{III-VII}$), 81.6 (C-4^{I}), 81.5 (C-5^{I}), 80.7 (C-2^{I}), 72.8 (C-3^{I}), 73.4, 73.3 (C-$3^{III-VII}$), 72.9, 72.8, 72.6 (C-$2^{III-VII}$), 72.3, 72.2, 72.1, 72.0 (C-$5^{III-VII}$), 64.6 (CH_2O-), 60.6, 60.4, 60.4, 60.3 (C-6^{I-VII}), 54.8 (CH_3O-).ESI-MS: *m/z* 1426.84 $[M + H]^+$, 713.97 $[M + 2H]^{2+}$. Anal. Calcd. for $C_{58}H_{83}N_5O_{36}$: C 48.84, H 5.87, N 4.91. Found: C 48.51, H 5.69, N 4.59.

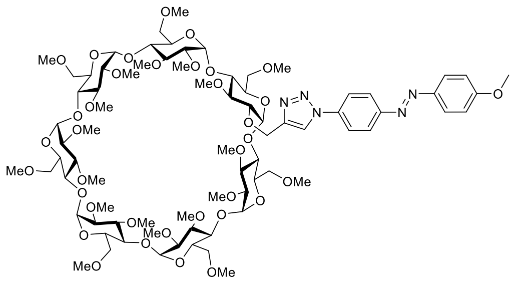

(*E*)-2^{II-VII}-3^{I-VII}-6^{I-VII}-Eicosamethyl-2^{I}-*O*-{[1-(4-((4-methoxyphenyl)diazenyl)phenyl)-1*H*-1,2,3-triazol-4-yl]methyl}cyclomaltoheptaose (3). To a solution of **2** (122 mg, 0.088 mmol) in dry DMF (8.5 mL), NaH (60%, 274 mg, 6.84 mmol, 4 eq) and MeI (320 µL, 5.13 mmol, 3 eq) were added at 0 ºC, under N_2 atmosphere. The reaction mixture was stirred for 14 h at RT, quenched with H_2O (1 mL) and extracted with Et_2O (3 × 25 mL). The organic layer was dried ($MgSO_4$), concentrated and purified by column chromatography (Hex → 2:1 EtOAc-Hex → EtOAc → 20:1 EtOAc-EtOH) to give **3**. Yield: 121 mg (83%). R_f 0.59 (30:6:3 EtOAc-EtOH-

H_2O); $[\alpha]_D$ = +102.7 (c 0.4, MeOH). ^{1}H NMR (600 MHz, CD_3OD): δ 8.77 (s, 1 H, C*H*-triazole), 8.09-8.08 (m, 4 H, H-b, H-c), 7.97 (2 H, *J* = 9.0 Hz, H-c'), 7.13 (2 H, *J* = 8.9 Hz, H-b'), 5.21-5.14 (m, 6 H, H-1^{II-VII}), 5.11 (d, 1 H, $J_{1,2}$ = 3.7 Hz, H-1^{I}), 4.99, 4.93 (2d, 2 H, $^2J_{H,H}$ = 12.4 Hz, *C*H_2O-Azo), 3.96 (dd, 1 H, $J_{6a,6b}$ = 11.0 Hz, $J_{5,6a}$ = 4.4 Hz, H-6a^{VII}), 3.93 (s, 3 H, CH_3O-Azo), 3.93-3.76 (m, 13 H, H-5^{I-VII}, H-6a^{II-VII}), 3.69 (s, 3 H, 1 × CH_3O), 3.66-3.50 (m, 58 H, H-2^{I}, H-3^{I-VII}, H-4^{I-VII}, H-6b^{I-VII}, 12 × CH_3O), 3.41-3.36 (m, 21 H, 7 × CH_3O), 3.19-3.13 (m, 6 H, H-2^{II-VII}). ^{13}C NMR (150.9 MHz, CD_3OD): δ 162.9, 152.4, 146.8, 146.4, 138.0 (C-a, C-d, C-a', C-d', C-triazole), 124.7 (C-c'), 123.6 (C-b), 122.1 (CH-triazole), 120.7 (C-c), 114.1 (C-b'), 98.4, 98.2, 98.1, 98.1 (C-1^{I-VII}), 82.3 (C-3^{I}), 82.1, 82.0, 81.9, 81.8, 81.7, 81.4 (C-2^{II-VII}, C-3^{II-VII}), 80.3 (C-2^{I}), 79.6, 76.5, 79.3, 79.2, 78.9 (C-4^{I-VII}), 71.5, 71.4, 71.1 (C-6^{I-VII}), 71.0, 70.9, 70.8 (C-5^{I-VII}), 63.9 (*C*H_2O-Azo), 60.6, 60.5, 60.4, 60.3, 58.0, 57.9, 57.7, 57.6, 57.5 (CH_3O), 54.8 (CH_3O-Azo). ESI-MS: *m/z* 1707.26 $[M + H]^+$. Anal. Calcd. for $C_{78}H_{123}N_5O_{36}$: C 54.89, H 7.26, N 4.10. Found: C 54.67, H 6.98, N 3.84.

13. NMR (Freshly Prepared Solutions – *E*-Isomer) and MS Spectra of the New Compounds (Figures S1-S4).

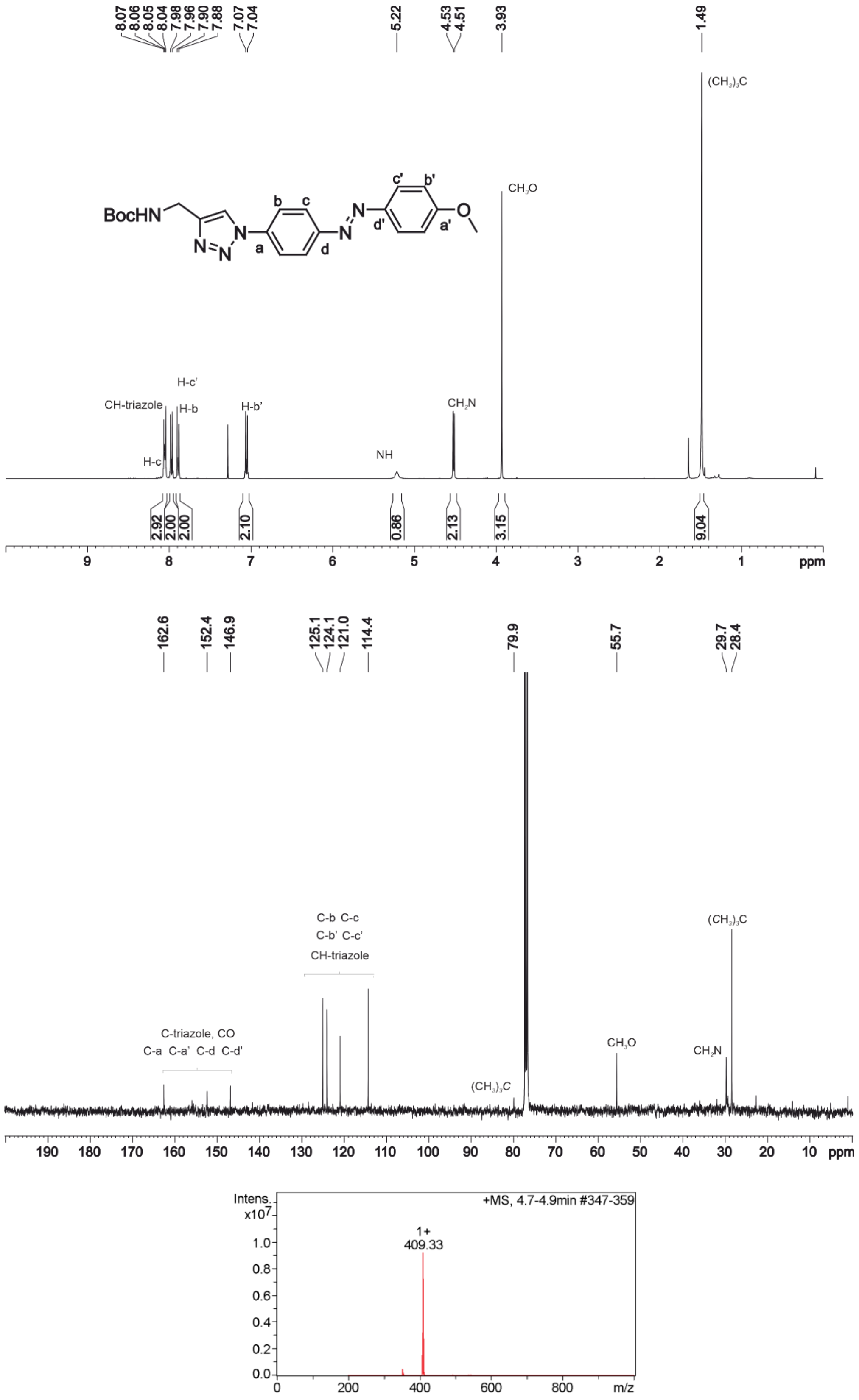

Figure S1. ^{1}H / ^{13}C NMR (400 MHz / 100.6 MHz, $CDCl_3$) and ESI-MS spectra of **1-Boc**.

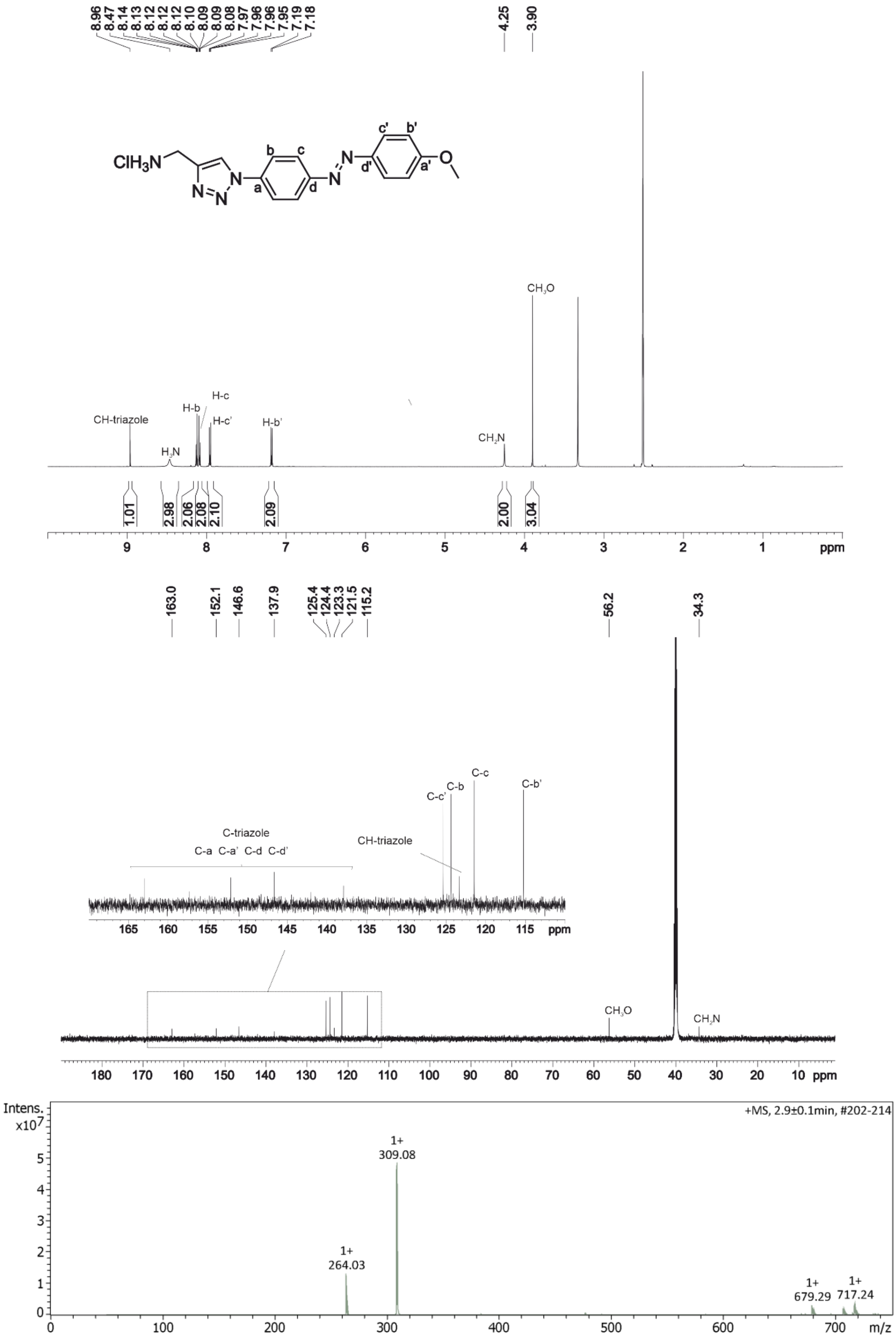

Figure S2. ^{1}H / ^{13}C NMR (600 MHz / 150.9 MHz, CD_3OD) and ESI-MS spectra of **1**.

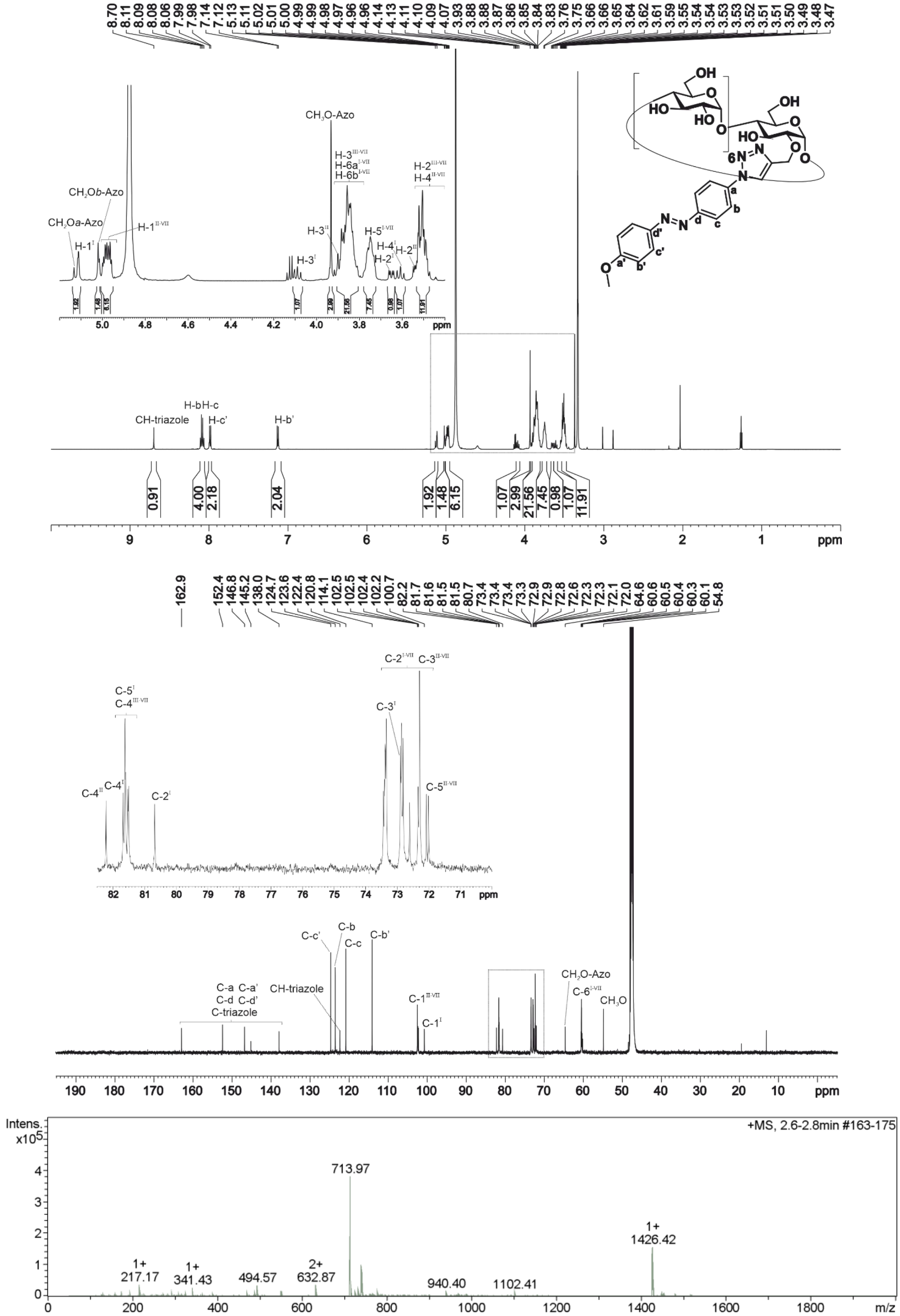

Figure S3. ^{1}H / ^{13}C NMR (600 MHz / 150.9 MHz, CD_3OD) and ESI-MS spectra of **2**.

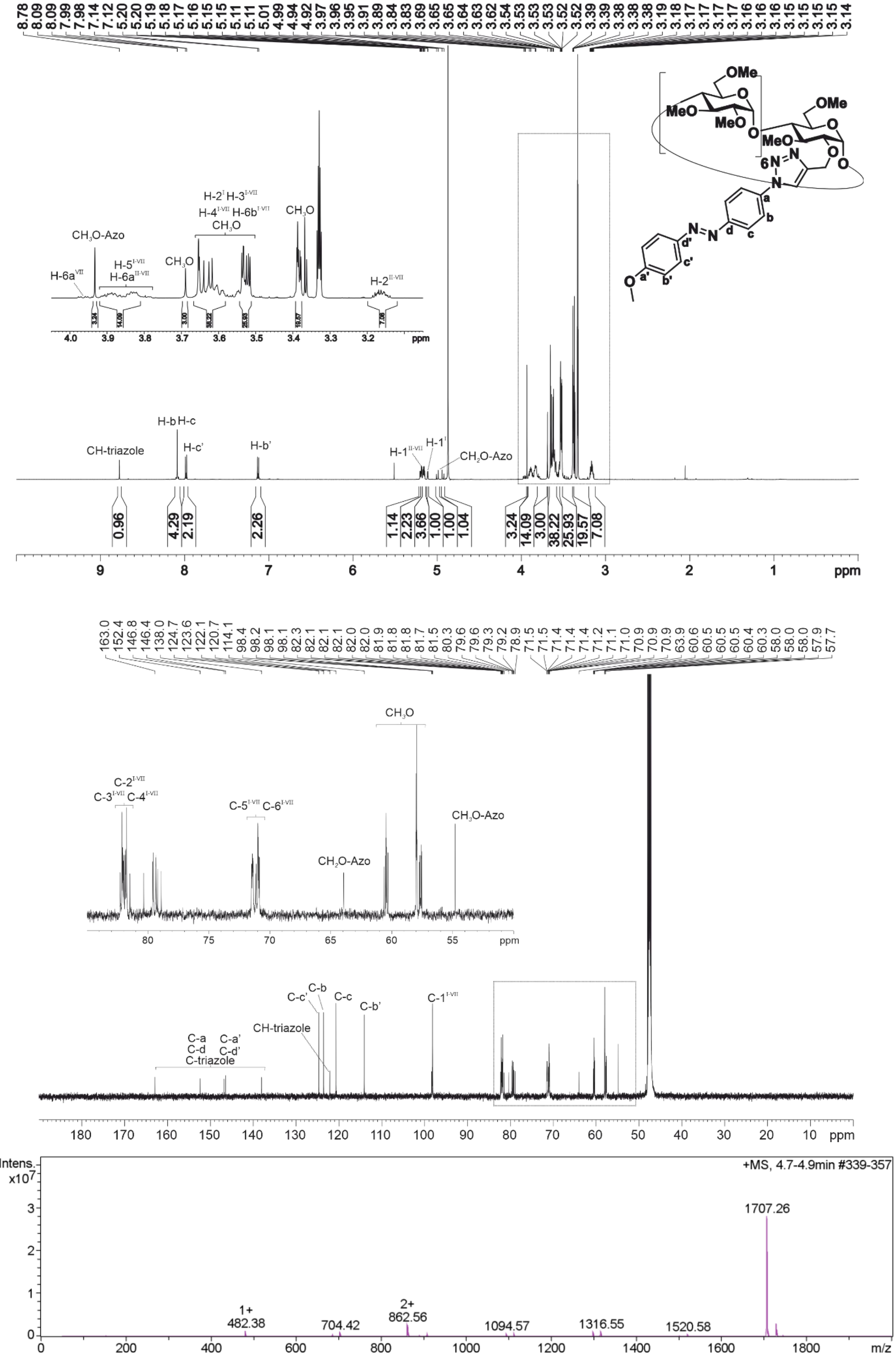

Figure S4. ^1H / ^{13}C NMR (600 MHz / 150.9 MHz, CD_3OD) and ESI-MS spectra of **3**.

14. 1-*E* / 1-*Z* Photoconversion (Figures S5-S6).

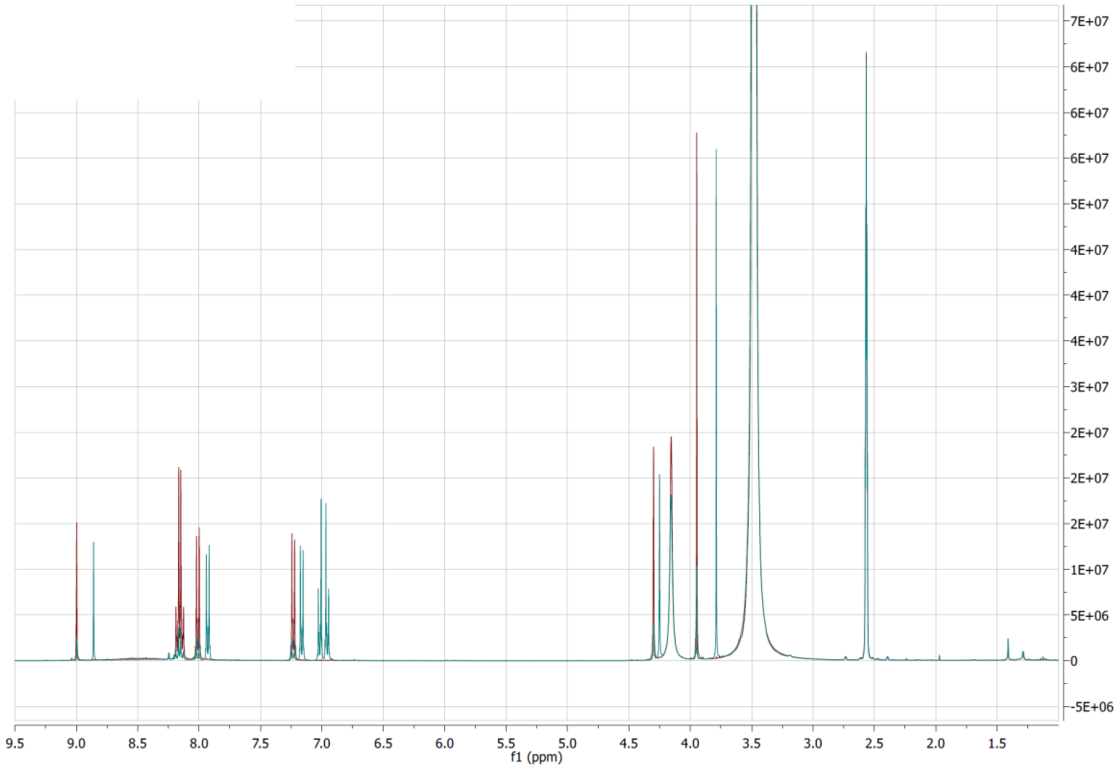

Figure S5. ^{1}H NMR Spectrum (500 MHz) for a fresh aqueous solution of **1** before (red line; *E*-isomer) and after irradiation at 365 nm (blue line – mostly Z-isomer).

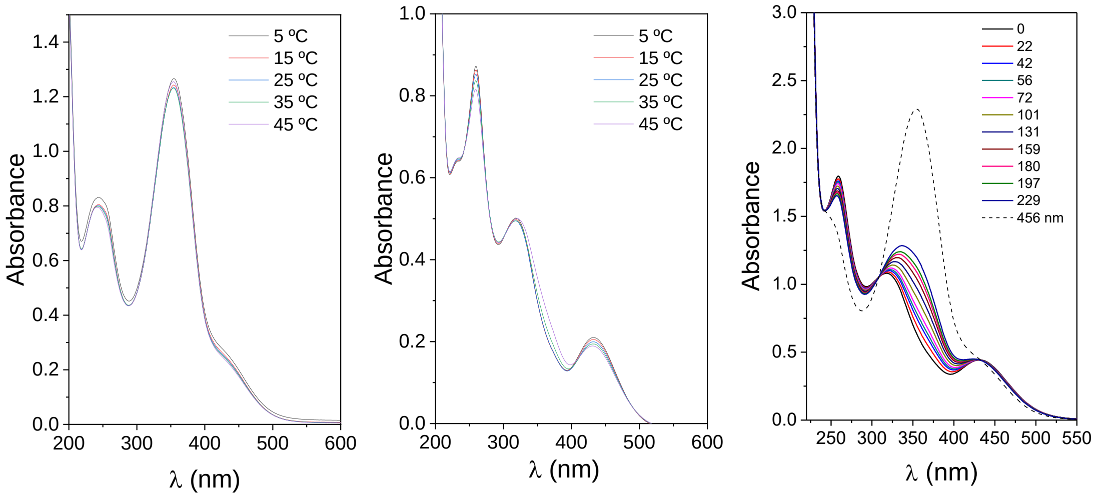

Figure S6. Left and middle panels: Absorption spectra for a 0.7 mM **1** in water before irradiation and after irradiation at 365 nm to reach PSS_{365}, respectively, at different temperatures. Experiments were carried out starting from 5 °C and maintaining the sample at each temperature for 30 min (2 mm optical path cuvettes). Right panel: Absorption spectra monitored at different times for a [**1**] ≈ 0.1 mM water solution after irradiation at 365 nm at 37ºC (10 mm optical path cuvettes were used). The dashed line corresponds to the spectrum for the solution after irradiation with blue light of 456 nm.

15. Photoisomerization and Thermal Isomerization Studies (Figure S7).

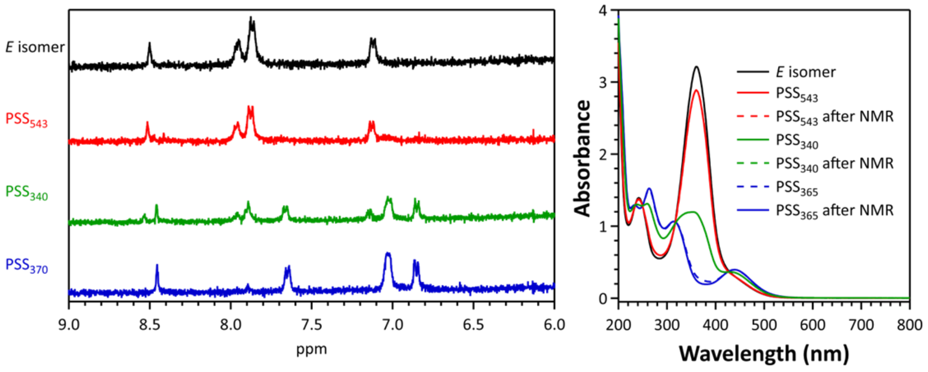

Figure S7. Partial ^{1}H NMR spectra (left) and absorption spectra (right) of **2** in D_2O (400 MHz) as pure *E*-isomer and at PSS_{340}, PSS_{365} and PSS_{543}.

16. Determination of the Half-Life (Figure S8).

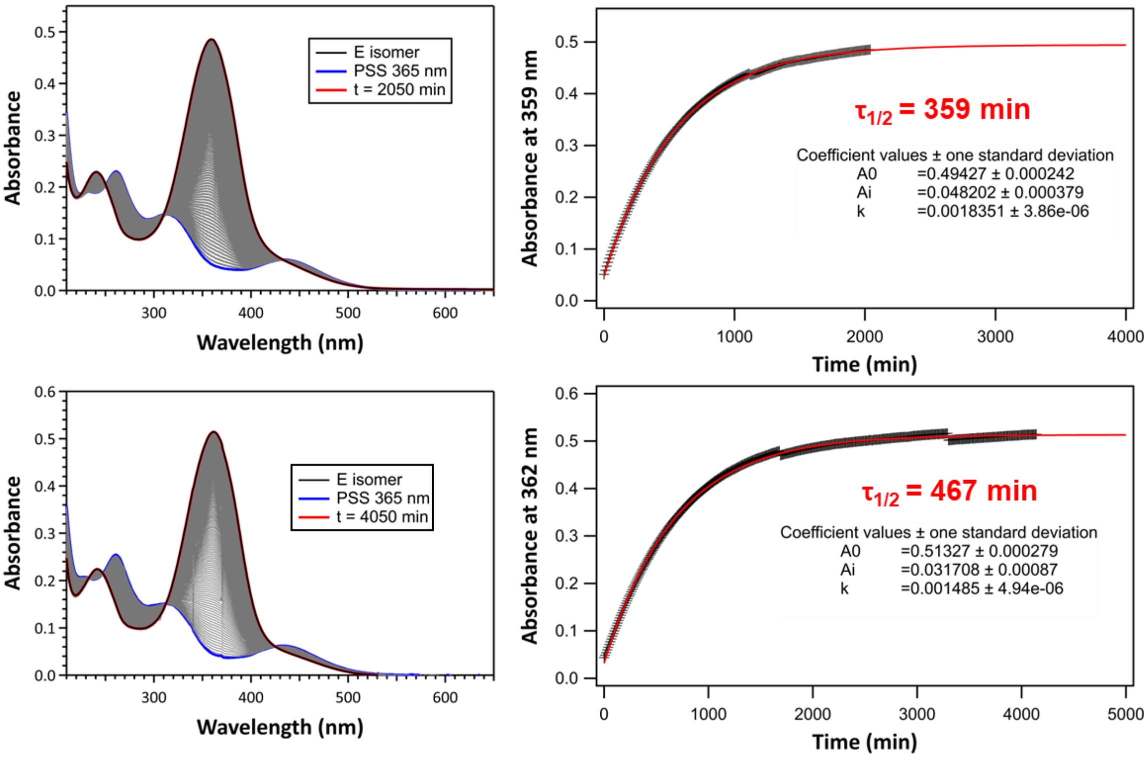

Figure S8. Time-dependent absorption profiles (PSS_{365}) of **2** (upper panel) and **3** (lower panel) at 37 °C in H_2O.

17. Determination of the Molar Absorption Coefficient and Fatigue Resistance Measurements (Figure S9).

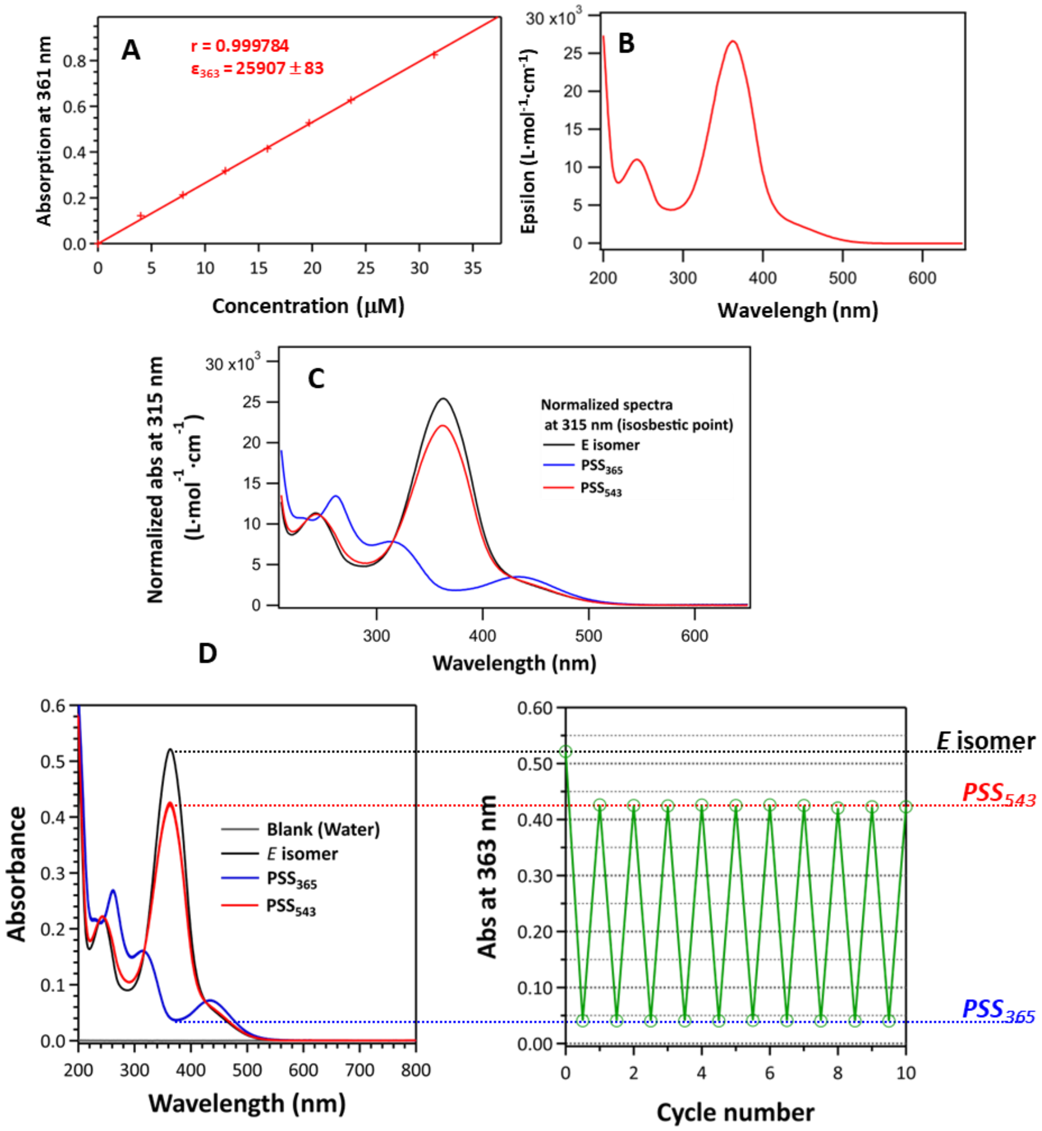

Figure S9. A) Determination of the molar absorption coefficient for **3** in H_2O from the plot of the absorbance at 361 nm vs concentration (red crosses), and the corresponding linear regression (red line). B) Calculated epsilon spectrum of **3** in H_2O obtained from different daughter solutions and representing the average value of the normalized spectra at the maximum of wavelength (361 or 363 nm). C) Photoisomerization studies of **3** in H_2O: the black spectrum corresponds to the freshly prepared solution (**3**-*E*), the blue one is recorded after irradiation at 365 nm (mostly **3**-*Z*) and the red one is recorded after irradiation at 543 nm (mostly **3**-*E*); the spectra were normalized at 315 nm with the corresponding Epsilon value. D) Fatigue resistance of **2** monitored by measuring the intensity of the absorption band at 361 nm under alternate 365 nm (P = 7.5 mW·cm^2) / 543 nm (P = 5.4 mW·cm^2) irradiation cycles in H_2O. Measurements were performed at 25 °C.

18. Fluorescence Spectroscopy Studies

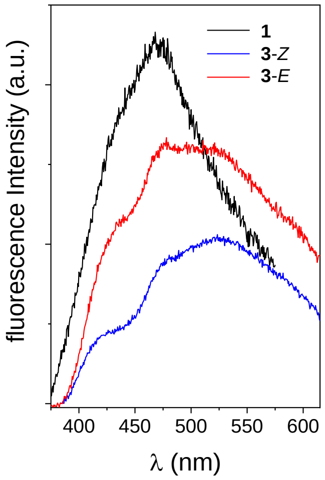

Figure S10. Emission spectra upon excitation at 335 nm for 0.7 mM **1** in H_2O (freshly prepared solution, major **1**-*E*; black line) and 0.68 mM **3** in H_2O before (red line; major *E*-isomer) and after irradiation at 365 nm to reach PSS_{365} (blue line; mostly *Z*-isomer).

19. UV-Vis and Induced Circular Dichroism Studies

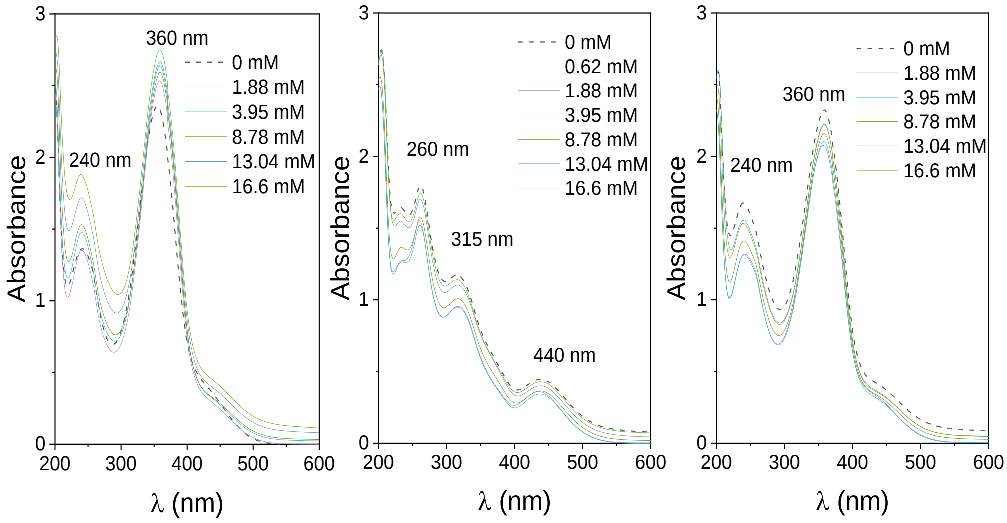

Figure S11. Absorption spectra for **1** in water in the absence and in the presence of βCyD at different concentrations before irradiation (left panel), after irradiation at 365 nm to reach PSS_{365} (middle panel) and after irradiation at 456 nm for 5 h (right panel) at 25 ºC. The concentration of **1** was fixed at 0.094 mM. [βCyD] were 0, 0.62, 0.96, 1.27, 1.88, 2.64, 3.95, 5.62, 8.78, 10.96, 13.04 and 16.6 mM, some of which are not shown in the spectra. 10-mm optical path cuvettes were used.

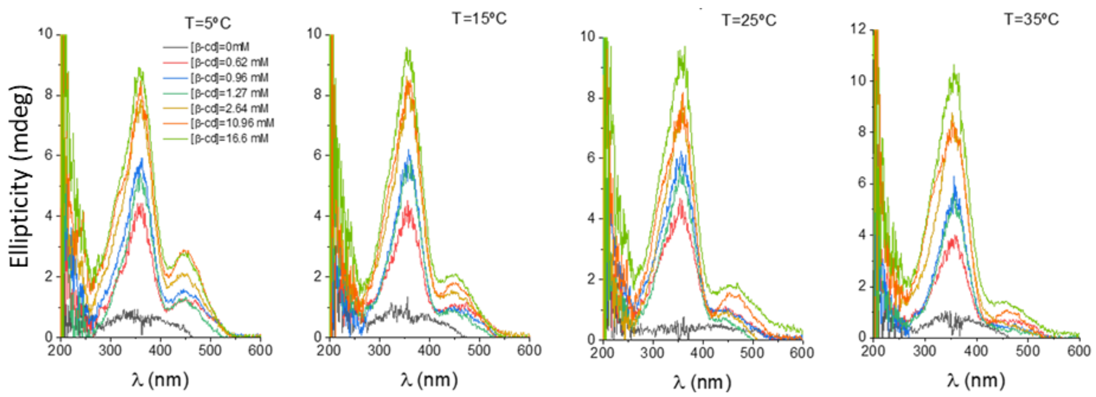

Figure S12. ICD plots of ellipticities vs wavelength at different temperatures for water solutions of **1 (**0.094 mM) in the absence and in the presence of βCyD before irradiation. [βCyD] used were 0, 0.62, 0.96, 1.27, 1.88, 2.64, 3.95, 5.62, 8.78, 10.96, 13.04 and 16.6 mM, some of which are shown in the plots. 10-mm optical path cuvettes were used.

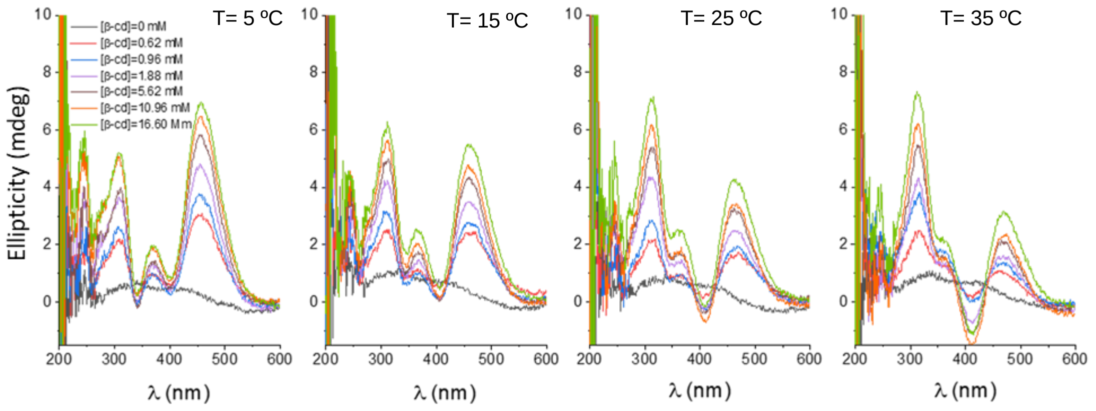

Figure S13. ICD plots of ellipticities vs wavelength at different temperatures for water solutions of **1 (**0.094 mM) in the absence and in the presence of βCyD after irradiation at 365 nm to reach PSS_{365}. [βCyD] used were 0, 0.62, 0.96, 1.27, 1.88, 2.64, 3.95, 5.62, 8.78, 10.96, 13.04 and 16.6 mM, some of which are shown in the plots. 10-mm optical path cuvettes were used.

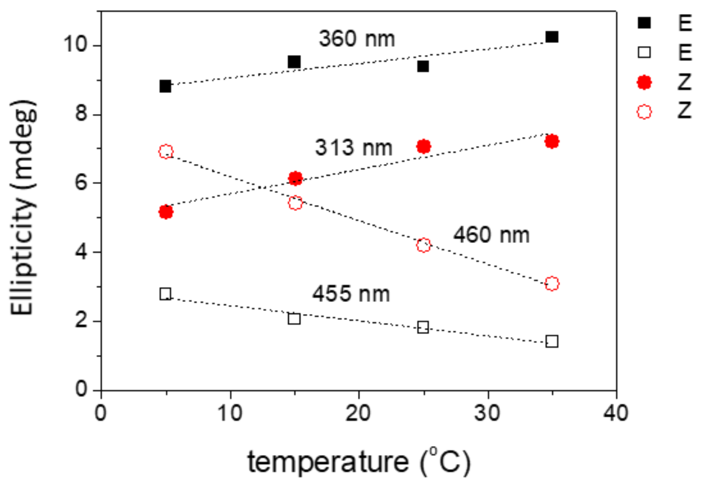

Figure S14. Ellipticity changes with temperature at the indicated wavelengths obtained from the ICD spectra at different temperatures of the **1** (0.094 mM) in water in the presence of βCyD (16.6 mM) before (squares) and after irradiation at 365 nm to reach PSS_{365} (circles).

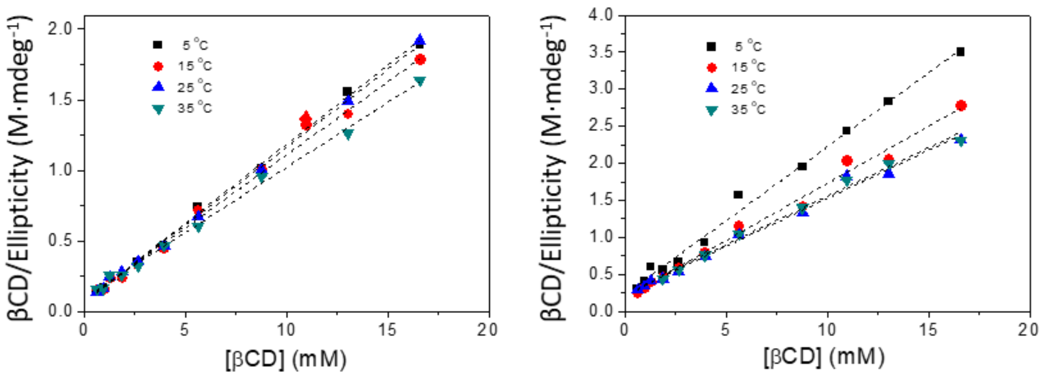

Figure S15. Linear plots of [βCyD]/ellipticity vs βCyD concentration at different temperatures, for a freshly prepared water solution of **1** (0.094 mM) before (left panel) and after irradiation at 365 nm to reach PSS_{365} (right panel) at several temperatures, obtained from the analysis of the ICD spectra shown in Figures S13 and S14. [βCyD] used were 0, 0.62, 0.96, 1.27, 1.88, 2.64, 3.95, 5.62, 8.78, 10.96, 13.04 and 16.6 mM.

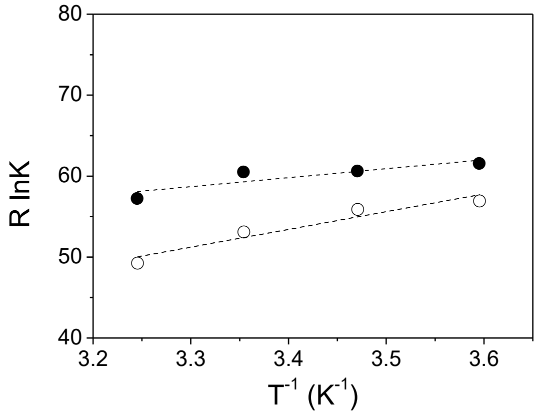

Figure S16. Van't Hoff representation of the association constants (*K*) obtained for **1** in water in the presence of βCyD before (black dots) and after irradiation at 365 nm to reach PSS_{365} (white dots).

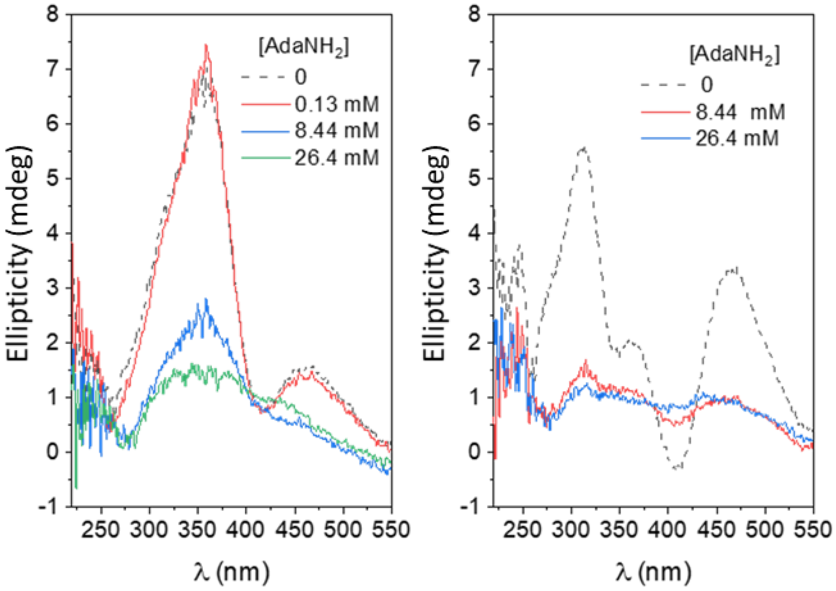

Figure S17. Left panel: ICD spectra for freshly prepared aqueous solutions of **1** (0.094 mM) containing βCyD (8.31 mM) in the absence and in the presence of adamantylamine ($AdaNH_2$) at different concentrations. Left panel: ICD spectra of three of the previous solutions after irradiation at 365 nm to reach PSS_{365}. 10 mm optical path cuvettes were used.

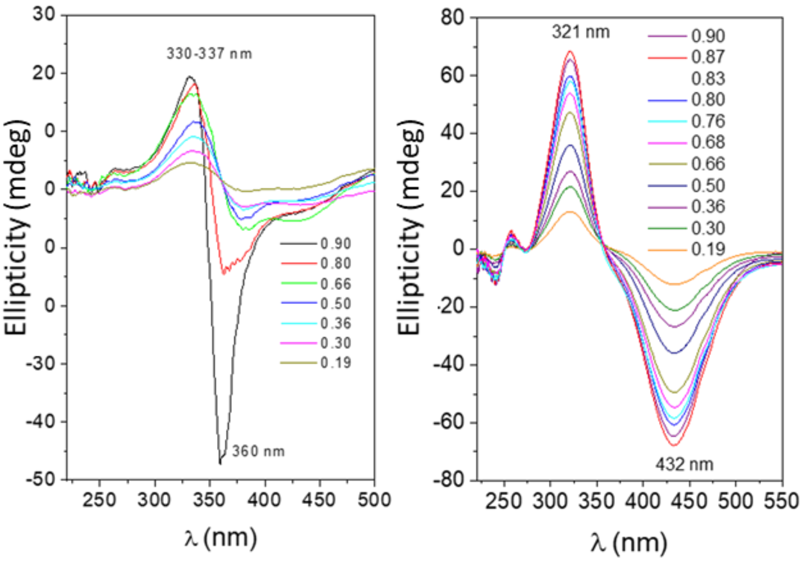

Figure S18. ICD spectra for freshly prepared aqueous solutions of **2** before (left panel) and after irradiation at 365 nm to reach PSS_{365} (right panel) at 25 ºC. [**2**] = 0.19, 0.30, 0.36, 0.50, 0.66, 0.68, 0.76, 0.83, 0.87 and 0.90 mM. 2 mm optical path cuvettes were used.

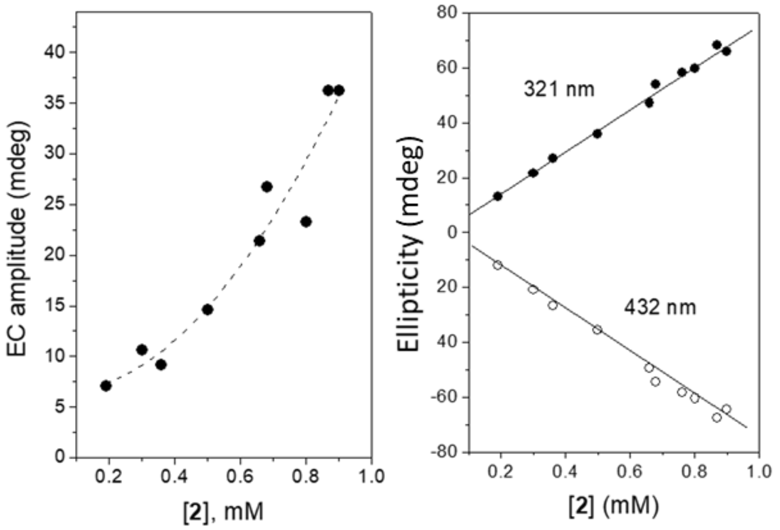

Figure S19. Exciton coupling amplitude obtained from ICD spectra analysis of freshly prepared aqueous solutions of **2** at 25 ºC (left panel) and plots of the ellipticity values for the main bands obtained after irradiation at 365 nm versus [**2**] (right panel).

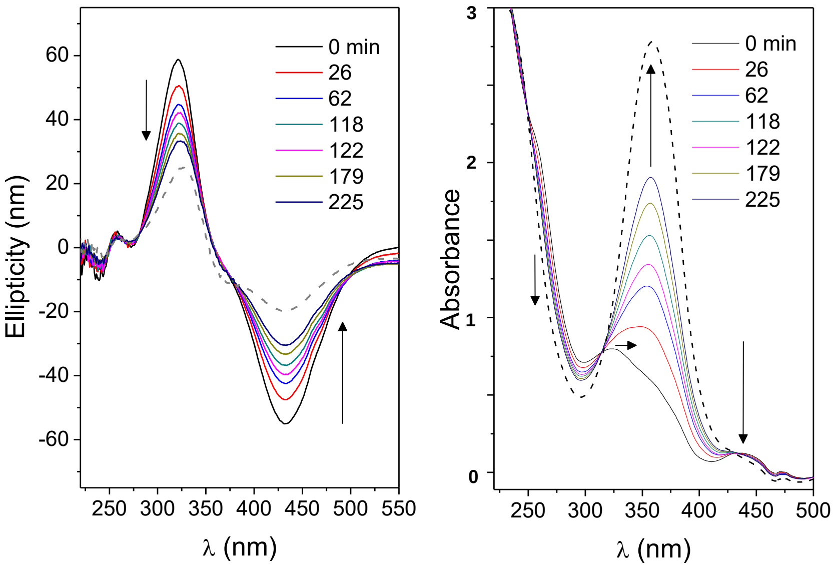

Figure S20. ICD (left panel) and absorption spectra (right panel) monitored at different times for **2** (0.901 mM) in aqueous solution after irradiation at 365 nm at 37 ºC. The dashed line corresponds to the spectrum of the same solution after irradiation at 456 nm.

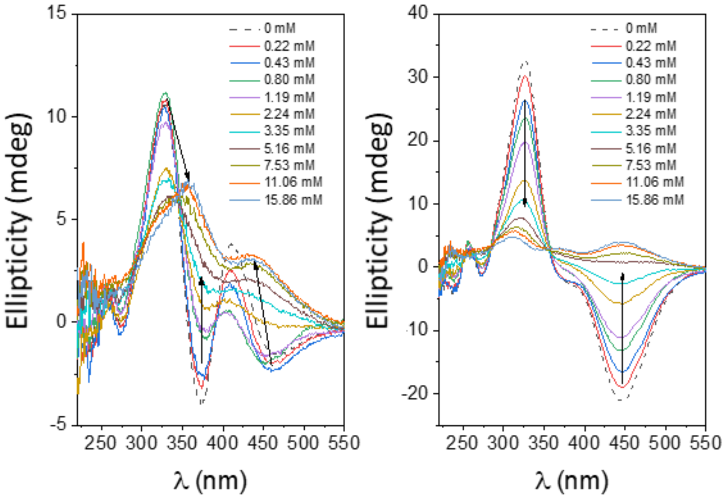

Figure S21. ICD spectra for a freshly prepared aqueous solution of **3** (0.10 mM) in the absence and in the presence of βCyD at increasing concentration before (left panel) and after irradiation at 365 nm (right panel) at 25 ºC. 10-mm optical path cuvettes were used.

20. NMR studies (Figures S22-S31).

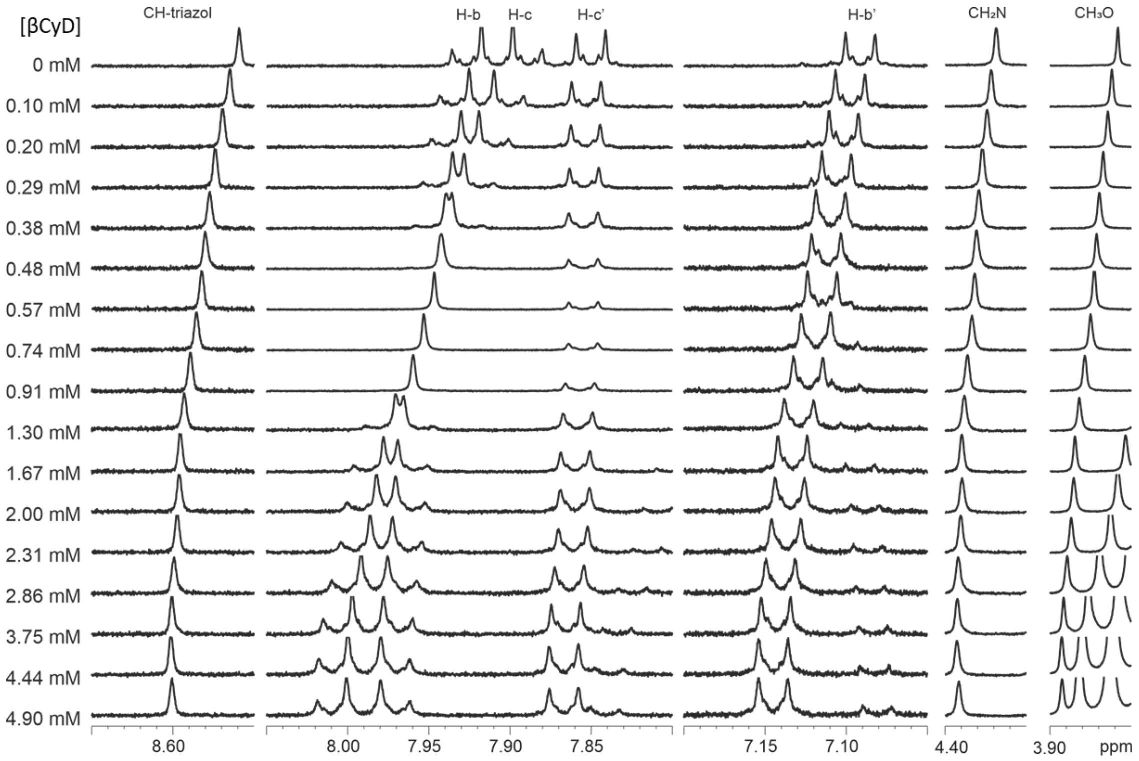

Figure S22. Selected regions of the ^{1}H NMR spectra (500 MHz, D_2O) of **1-*E*** (0.6 mM) in the absence and in the presence of increasing amounts of βCyD, showing the shift of CH-triazole, aromatic, CH_2N, and OCH_3 signals induced by the addition of the host.

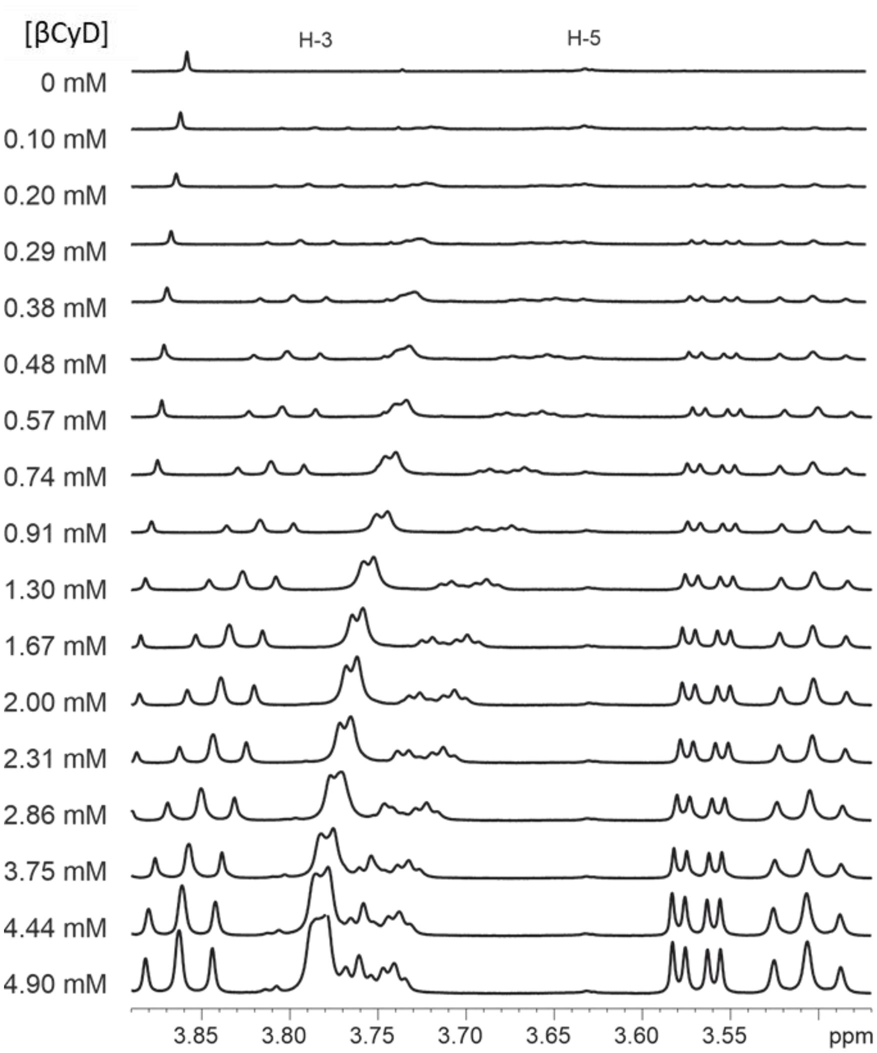

Figure S23. Selected regions of the ^1H NMR spectra (500 MHz, D_2O) of the **1-*E***/βCyD titration experiment showing the shift of H-3 and H-5 proton signals of the host.

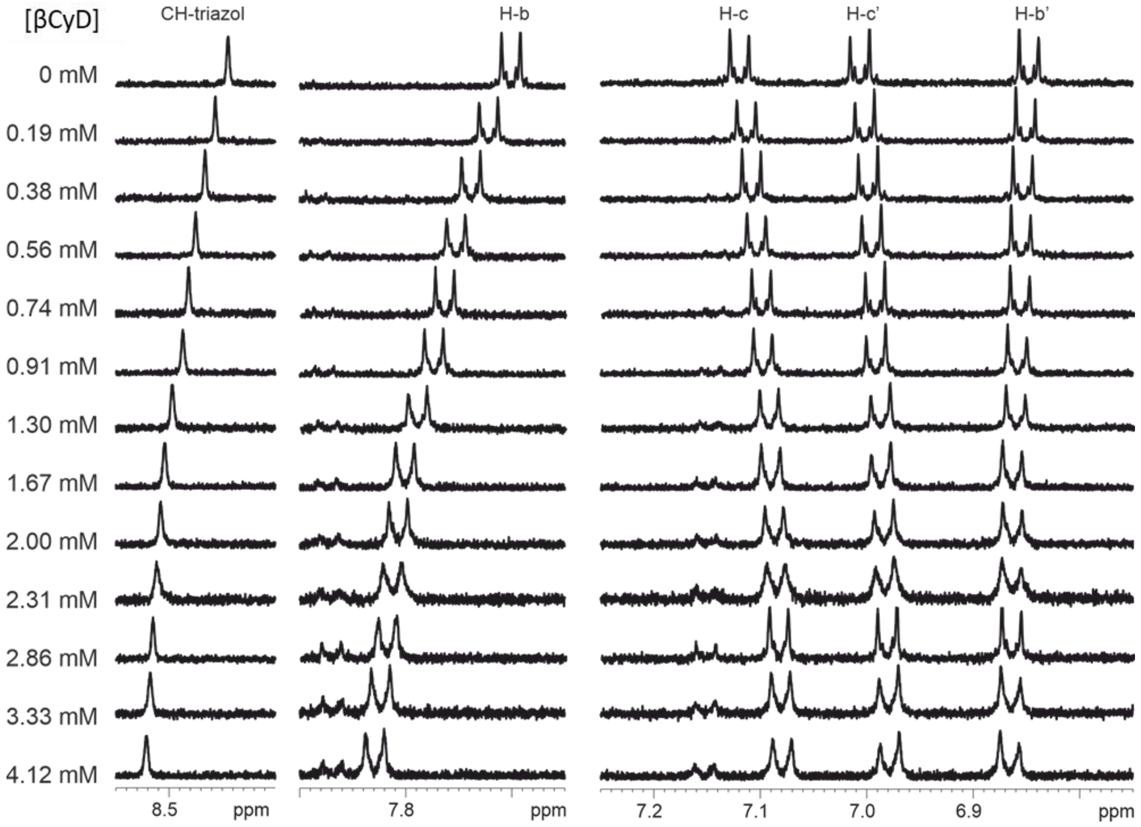

Figure S24. Selected regions of the ^{1}H NMR spectra (500 MHz, D_2O) of **1-*Z*** (0.6 mM) in the absence and in the presence of increasing amounts of βCyD, showing the shift of CH-triazole and aromatic signals induced by the addition of the host.

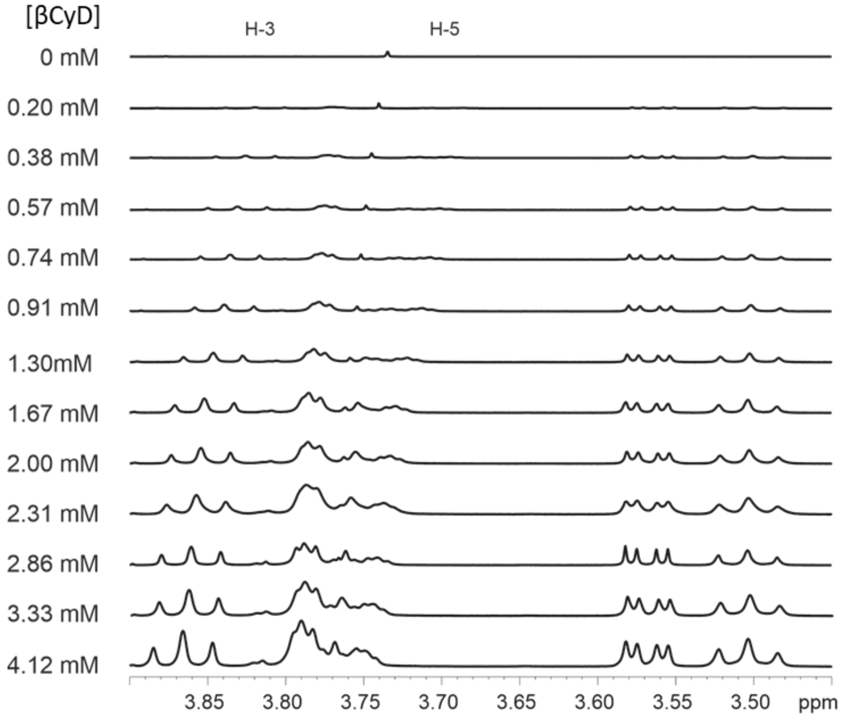

Figure S25. Selected regions of the ^{1}H NMR spectra (500 MHz, D_2O) of the **1-*E***/βCyD titration experiment showing the shift of H-3 and H-5 proton signals of the host.

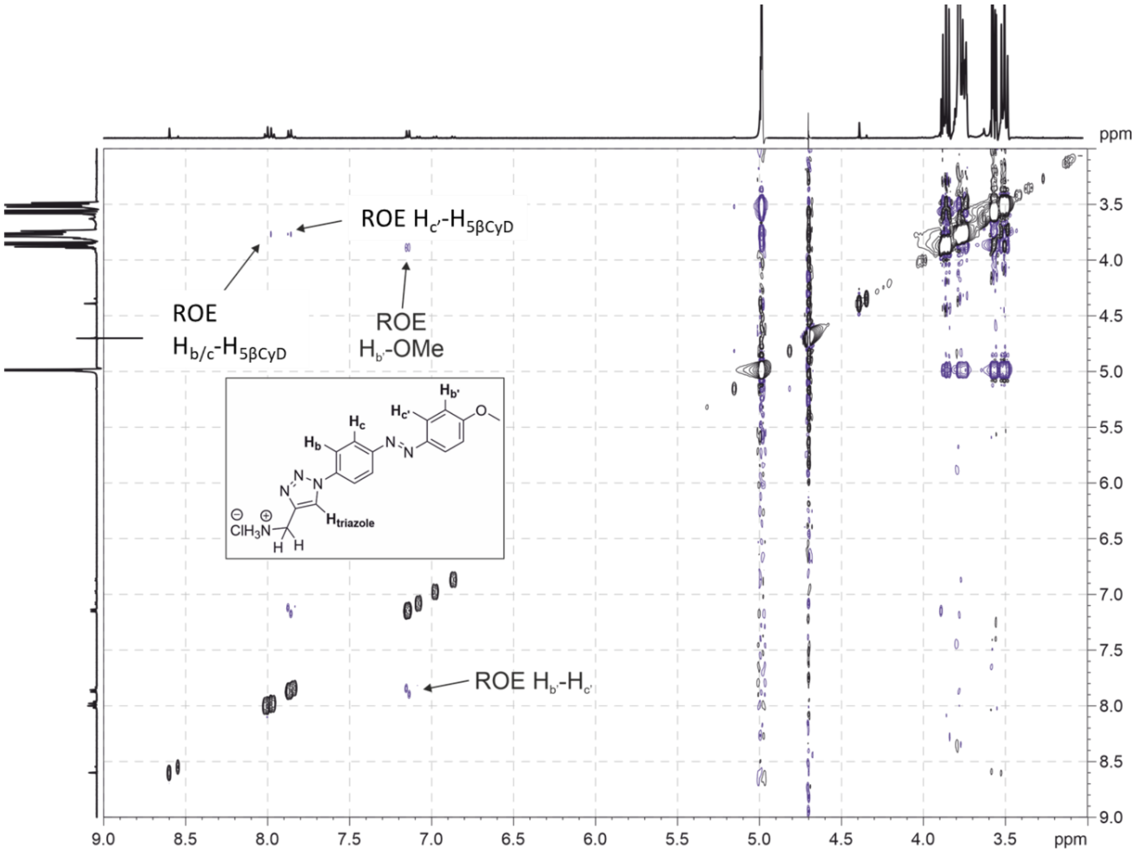

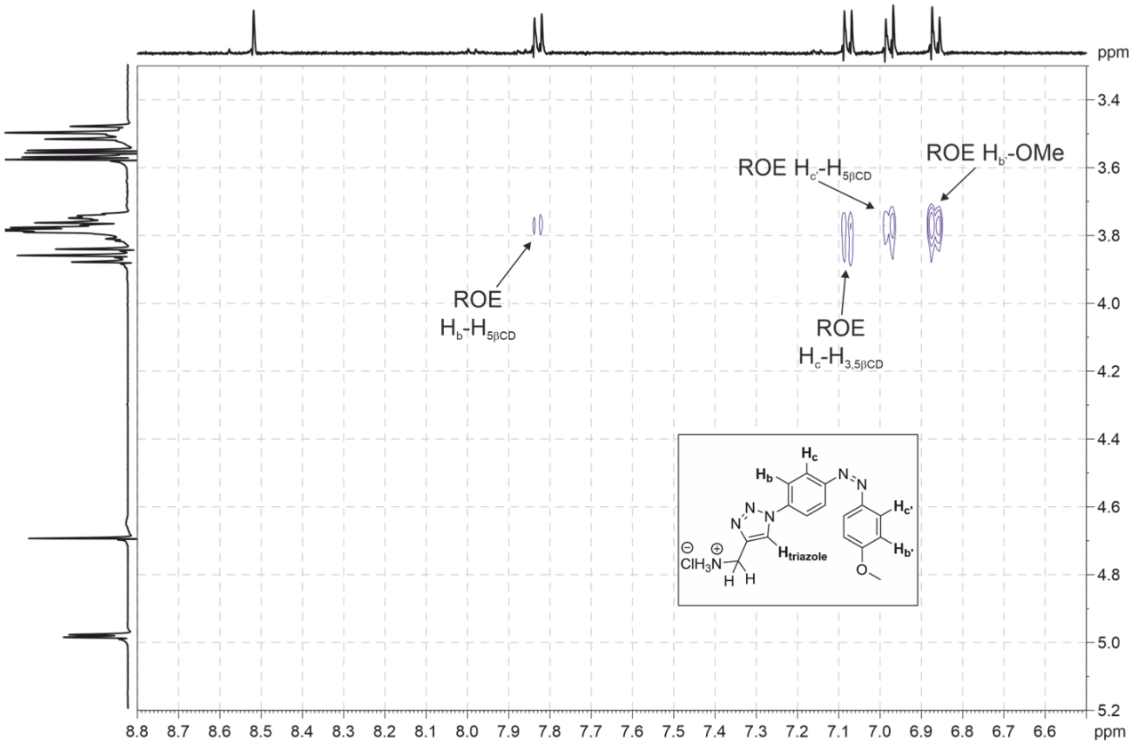

Figure S26. Full (upper) and selected region (lower) 2D ROESY spectrum (600 MHz, D_2O) of a mixture of **1**-*E* and βCyD (0.6 and 10 mM, respectively) showing diagnostic cross peaks. Mixing time: 0.7 s.

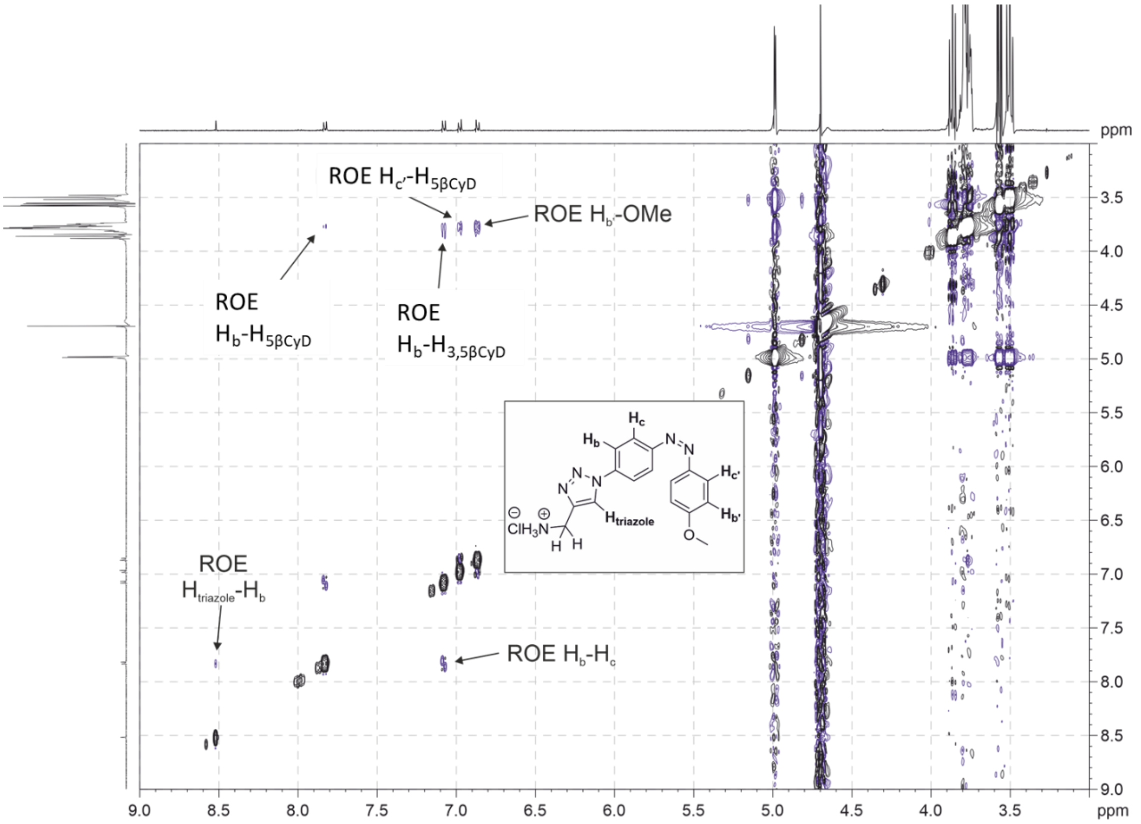

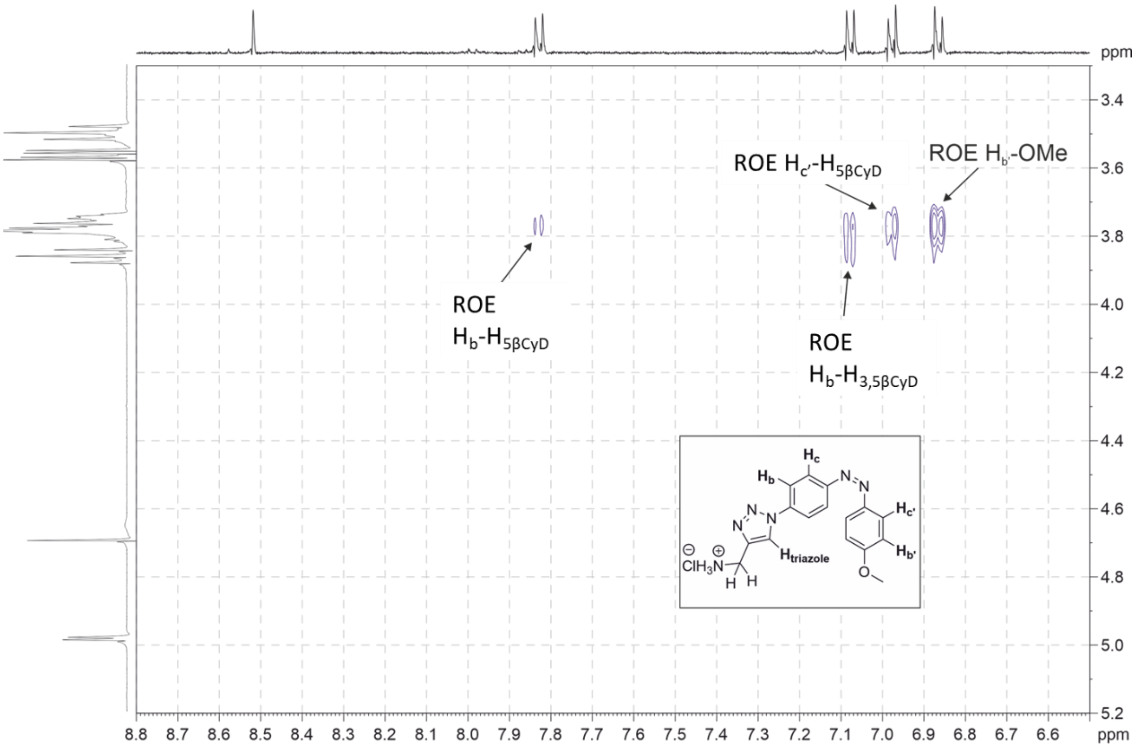

Figure S27. Full (upper) and selected region (lower) 2D ROESY spectrum (600 MHz, D_2O) of a mixture of **1**-*Z* and βCyD (0.6 and 10 mM, respectively) showing diagnostic cross peaks. Mixing time: 0.7 s.

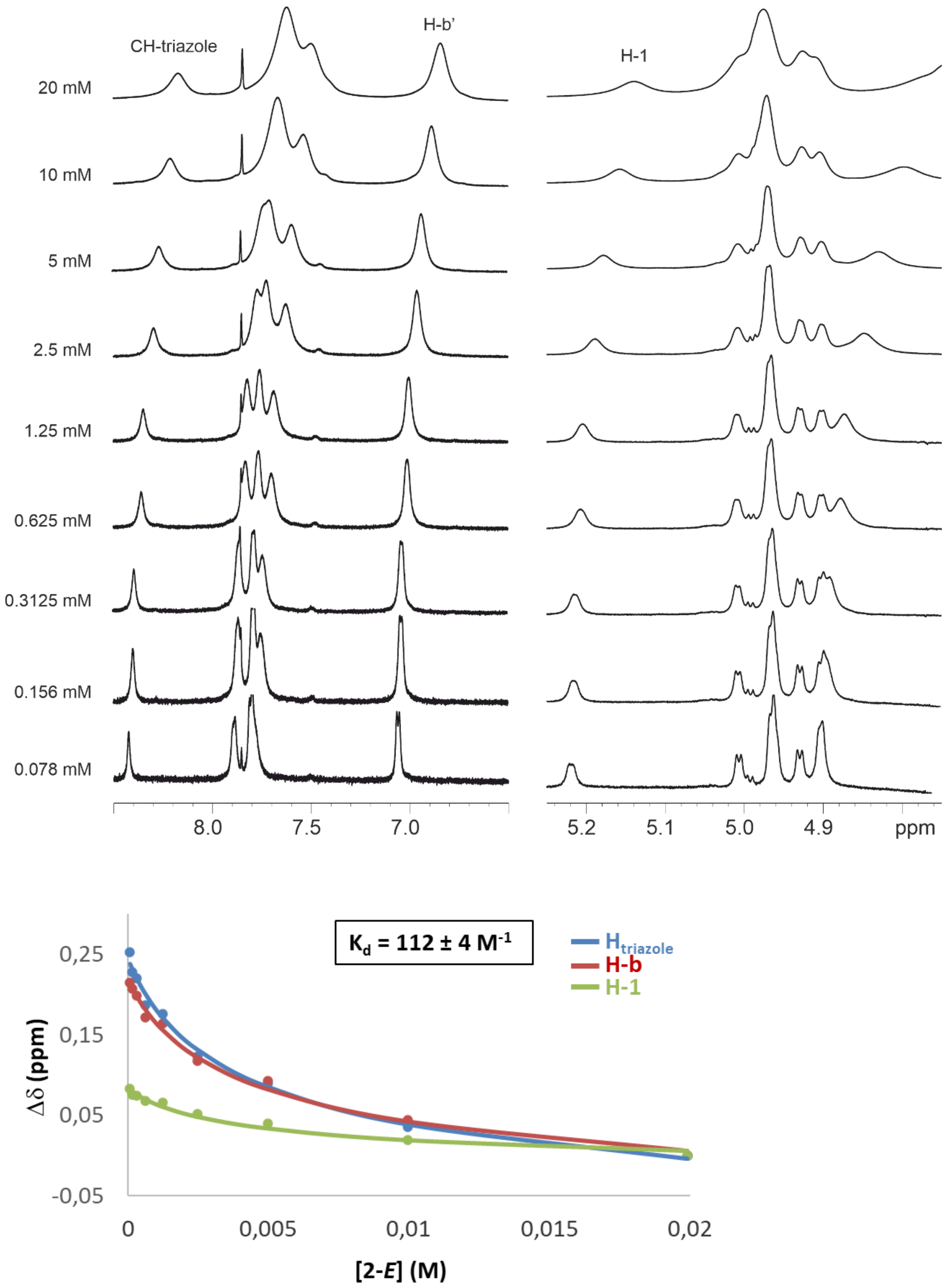

Figure S28. Selected regions of the concentration-dependent ^{1}H NMR (500 MHz) spectra of **2-*E*** in D_2O, showing the shift of the CH-triazole, aromatic, and H-1 (βCyD) signals (upper), and titration isotherms (lower) with indication of the derived dimerization constant (K_d) for (**2-*E***)$_2$ dimer formation.

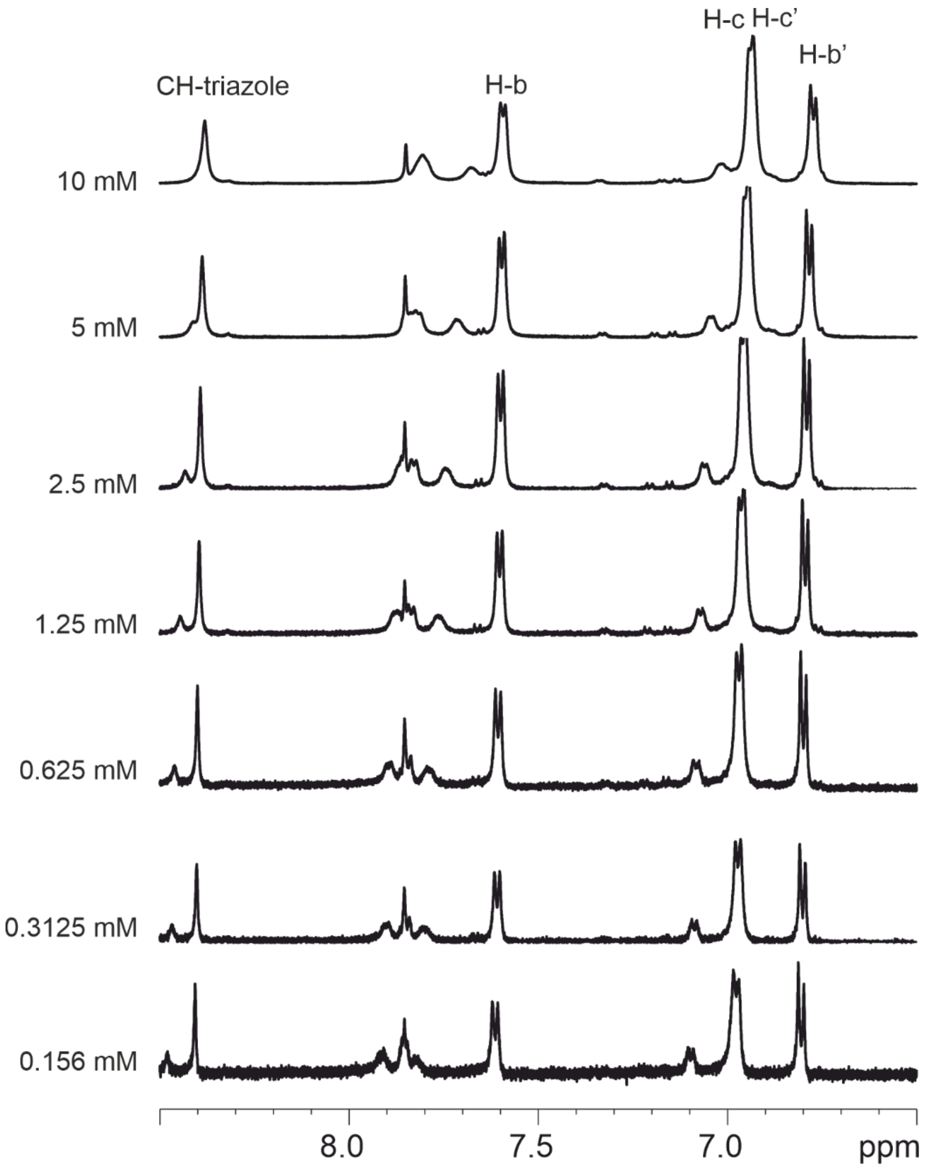

Figure S29. Selected region of the concentration-dependent ^{1}H NMR (500 MHz) spectra of **2-*Z*** in D_2O, showing the signals for the CH-triazole and aromatic protons

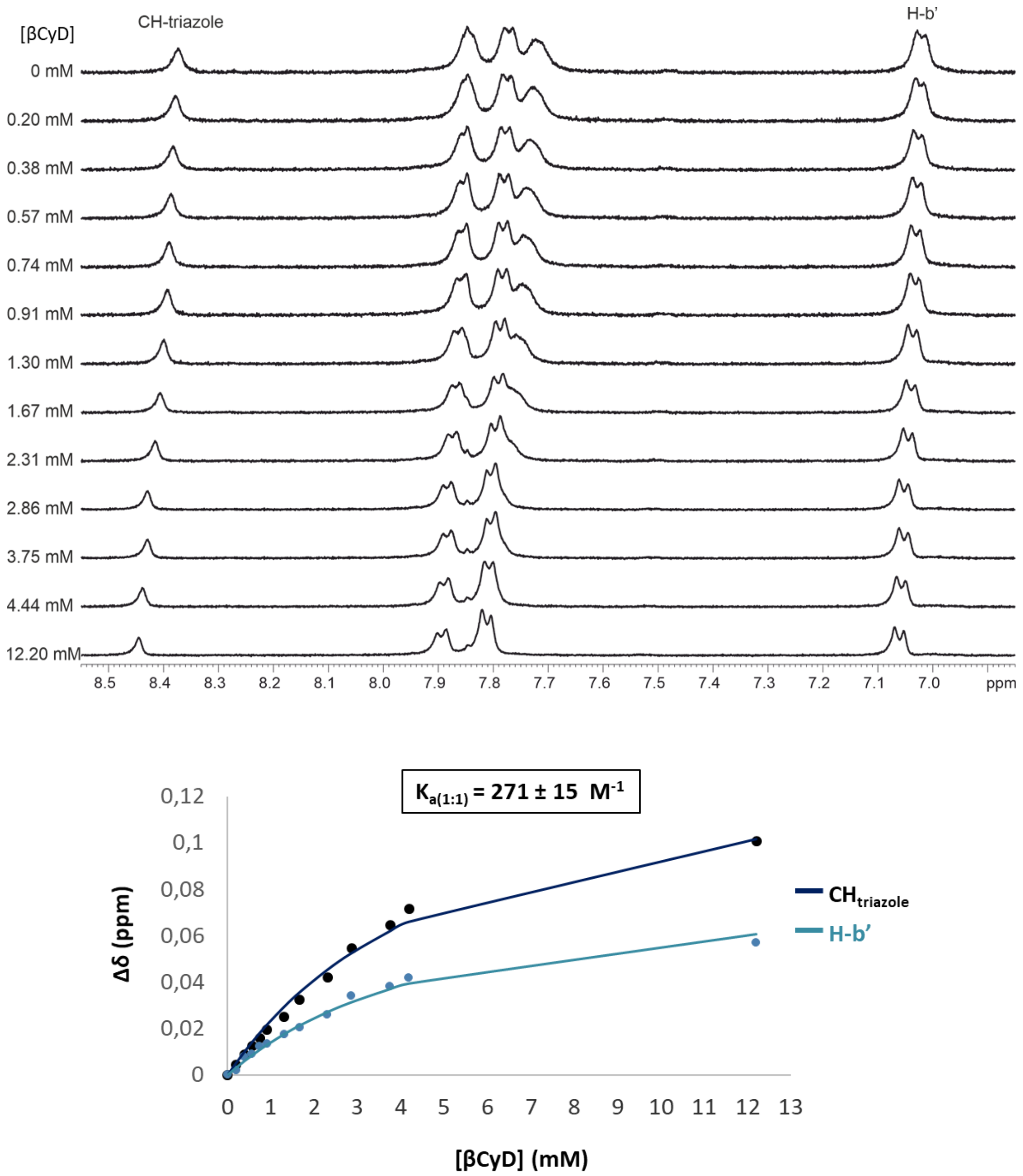

Figure S30. Selected regions of the ^{1}H NMR (600 MHz) spectra of **2-*E*** in D_2O (1 mM) in the absence and in the presence of increasing amounts of βCyD, showing the shift of the CH-triazole and aromatic proton signals (upper), and titration isotherms (lower) with indication of the derived apparent homodimerization constant ($K_{a(1:1)}$) for the formation of the **2-*E***:βCyD complex

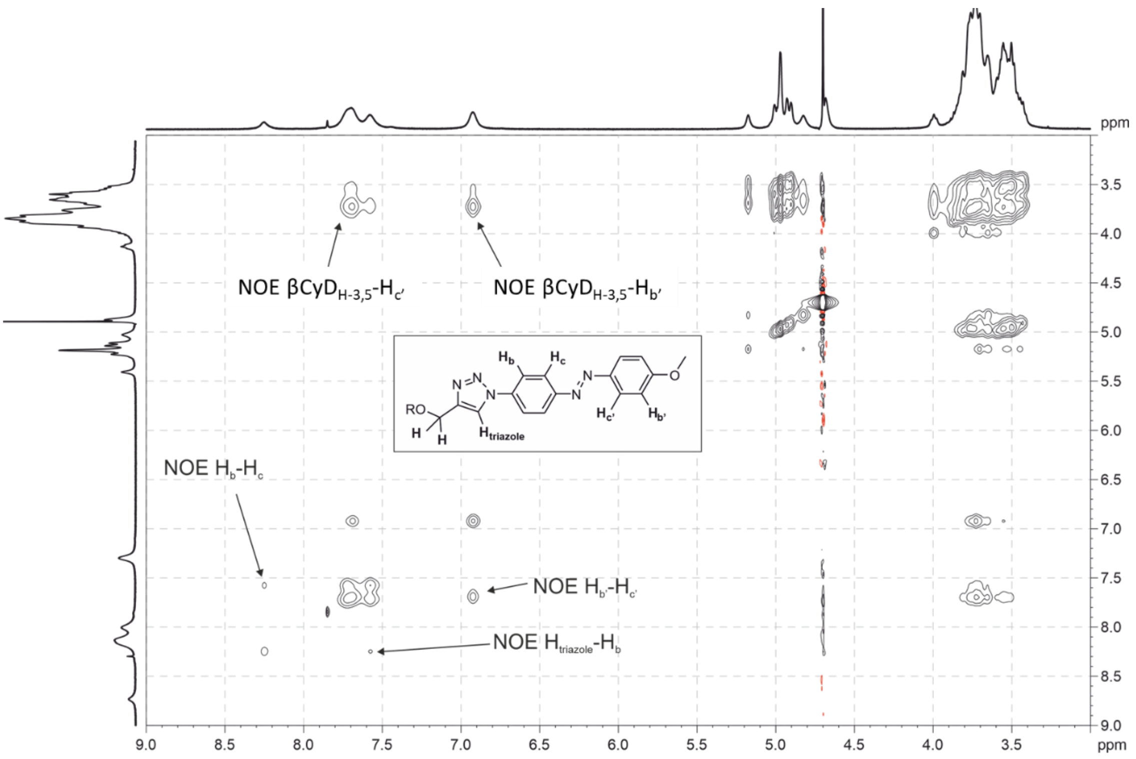

Figure S31. 2D NOESY spectrum (500 MHz, D_2O) of **2**-*E* (5 mM) showing diagnostic cross peaks. R represents the O-2-linked cyclodextrin moiety. Mixing time: 0.7 s.

21. Theoretical Studies (Figures S32-S38).

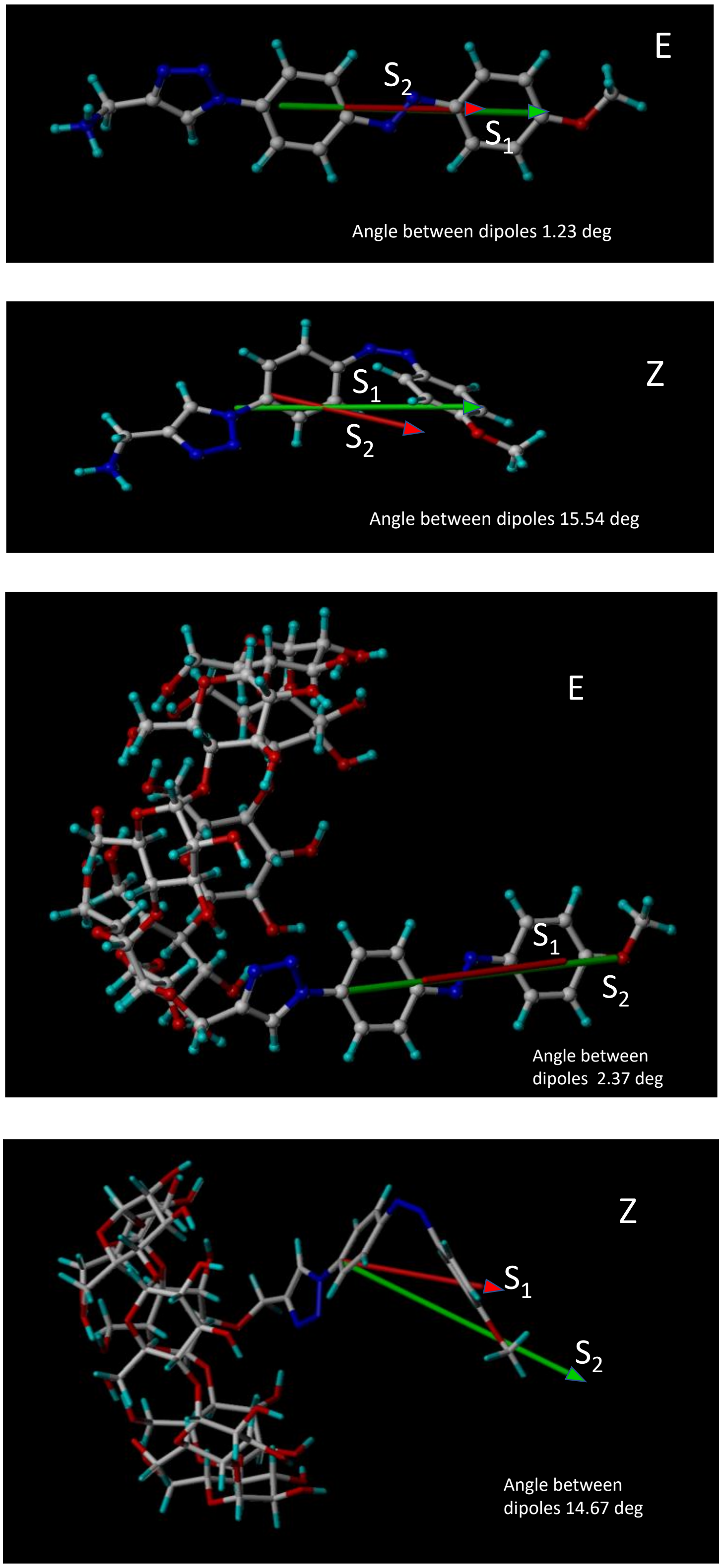

Figure S32. Dipole moments for the $S_0 \rightarrow S_1$ ($n \rightarrow \pi^*$) and $S_0 \rightarrow S_2$ ($\pi \rightarrow \pi^*$) transitions. The orientation and angle between them for the E- and Z-isomers of **1** (two upper panels) and **2** (two lower panels) are shown.

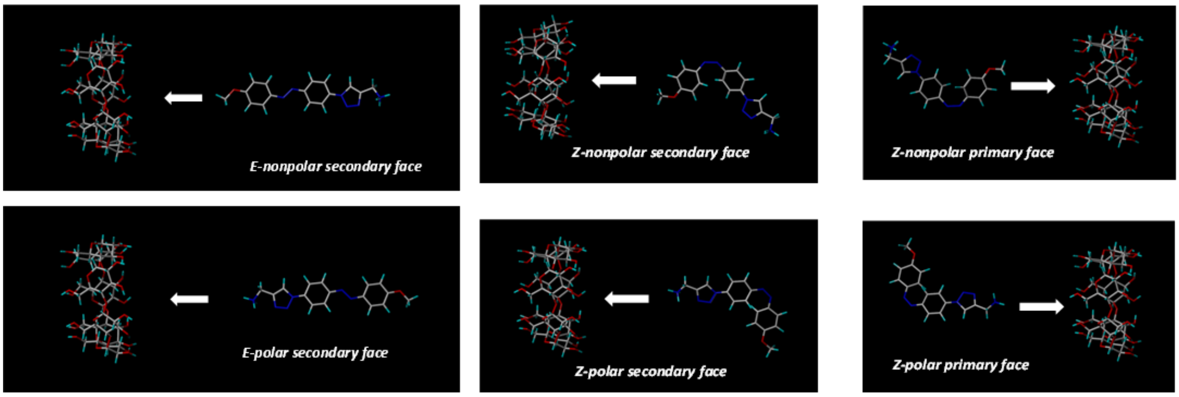

Figure S33. Most favorable orientations used for the *E*- and *Z*-forms of **1** approaching to βCyD by the secondary and primary faces along the *y* coordinate.

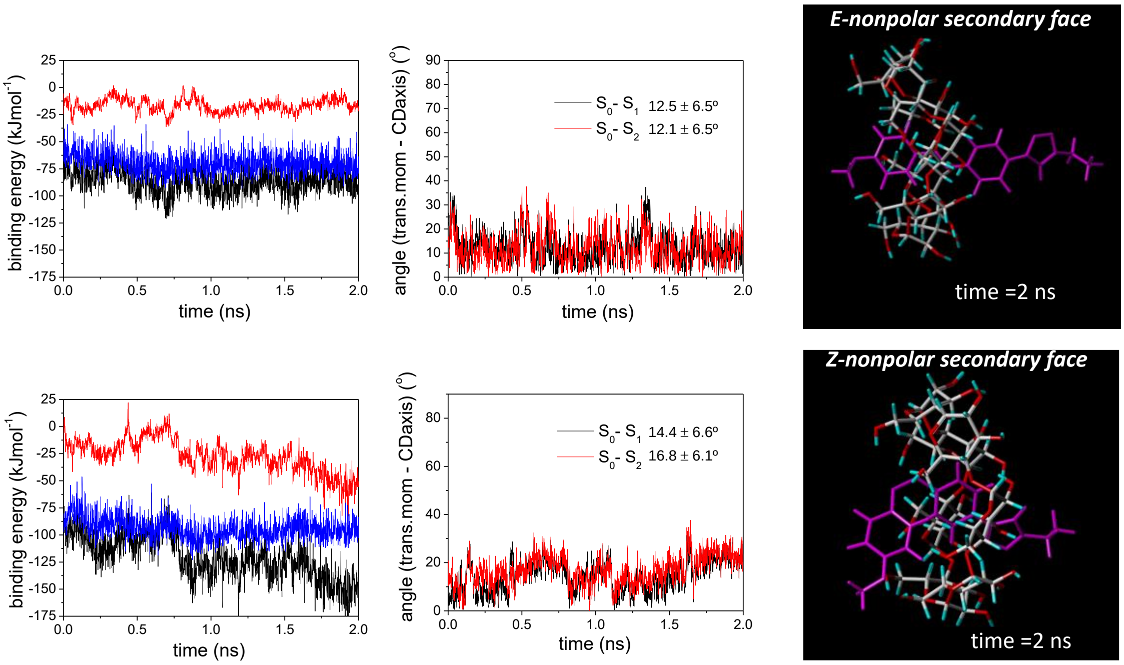

Figure S34. Histories for **1**-*E*/βCyD (upper) and **1**-*Z*/βCyD (lower) total binding energies (black) and electrostatics (red) and van der Waals (blue) contributions (left panels), angles between dipole moments for the $S_0 \rightarrow S_1$ (black) and $S_0 \rightarrow S_2$ (red) transitions and the seven-fold CyD main axis (middle panels) for approaches involving the nonpolar end of **1** and the secondary face of βCyD, (angle averages are indicated in the inserts), and structure of the complexes at the end of the MD trajectories (left panels). Results were obtained from the analysis of the 2 ns MD trajectories for the **1**-*E*/βCyD (and **1**-*Z*/βCyD) complex in a 48.86^3 (47.00^3) $Å^3$ periodic cubic box containing 5090 (4153) water molecules.

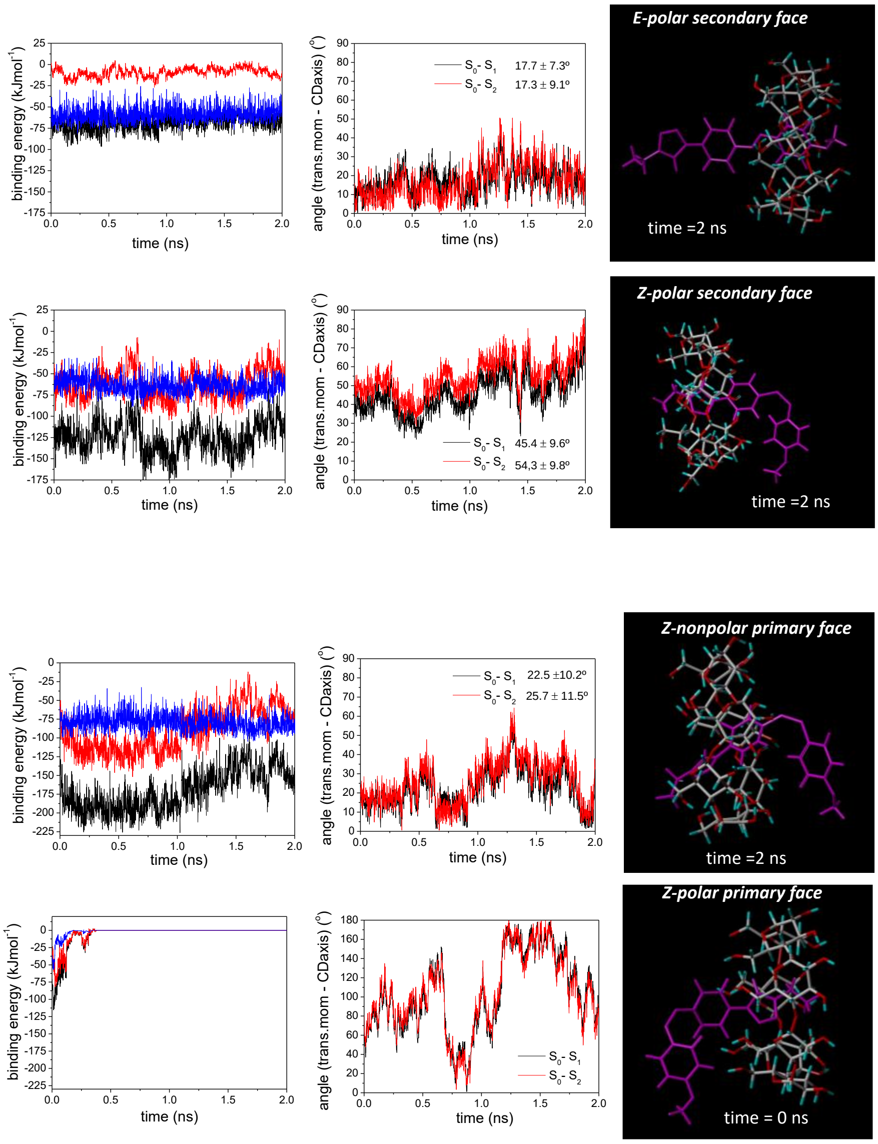

Figure S35. (left panels) Histories for **1**–βCyD total binding energies (black) and electrostatics (red) and van der Waals (blue) contributions and (middle panels) angles between dipole moments for the $S_0 \rightarrow S_1$ (black) and $S_0 \rightarrow S_2$ (red) transitions and the seven-fold CyD main axis for isomers *E* and *Z* (angle averages are indicated in the inserts) for other nonpolar or polar **1** approaches to βCyD by the secondary or primary faces. Structure of the complexes at the end of the MD trajectories or at t=0 (in case of dissociation). Results were obtained from the analysis of the 2 ns MD trajectories for the **1**-*E*/βCyD complex in a 48.84^3 Å^3 periodic cubic box containing 5099 water molecules and **1**-*Z*/βCyD complexes in periodic cubic boxes of 47.17^3, 47.33^3 and 47.05^3 Å^3 containing 4524, 4454 and 4426 water molecules respectively (from top to the bottom).

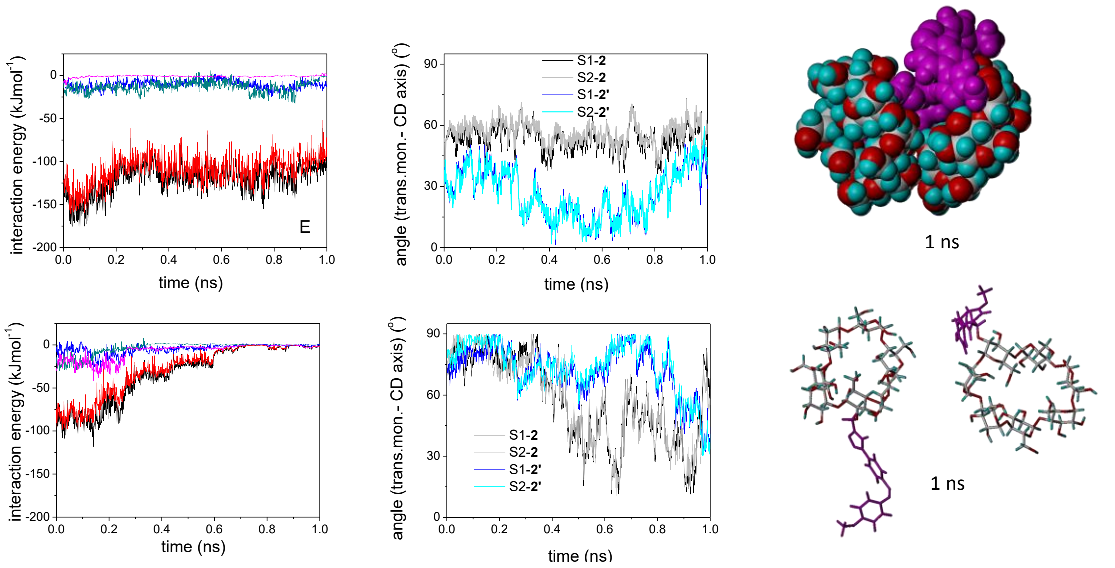

Figure S36. Left panels: Histories of the **2**–**2'** (total (black) interaction energies, as well as electrostatics (blue) and van der Waals (red) contributions, and the interaction energy between the azobenzene groups (green) and between the βCyD macrorings (magenta) obtained from the analysis of the 1 ns MD trajectories for the (**2**-*E*)$_2$ and (**2**-*Z*)$_2$ dimers in a 47.20^3 Å^3 periodic cubic box containing 4515 water molecules (upper and lower lane, respectively), which started on the MBE structures obtained by MM (Figure 14 in the main manuscript, 90 deg). Middle panel: Histories of the angle between the main βCyD axis and transition dipoles moments $S_0 \rightarrow S_1$ and $S_0 \rightarrow S_2$ for the corresponding azo moiety groups. Right panel: Snapshot of the structures at the end of the MD trajectory.

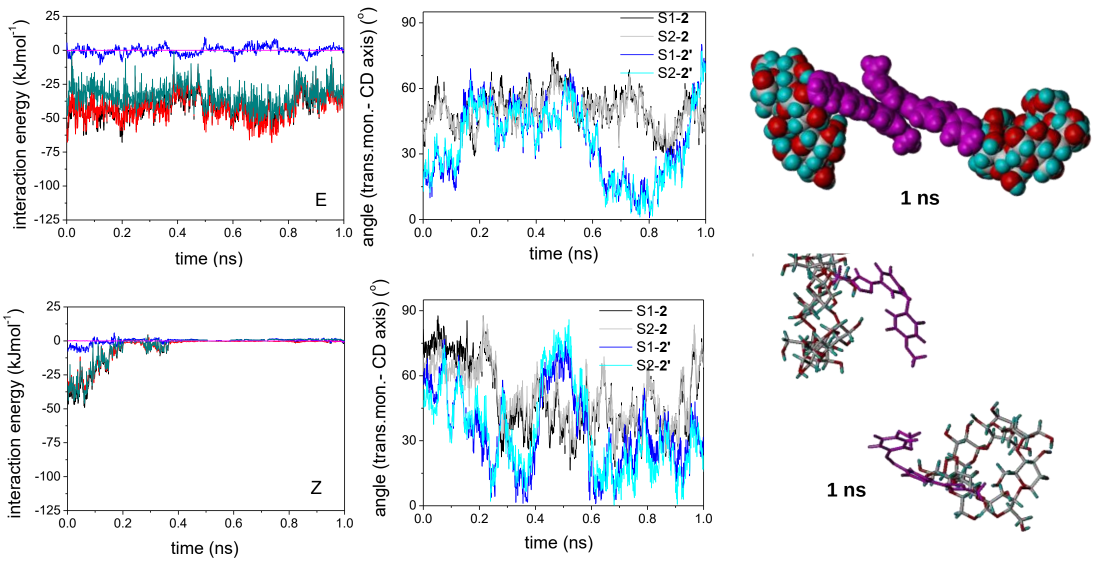

Figure S37. Left panels: Histories of the **2**–**2'** (total (black) interaction energies, as well as electrostatics (blue) and van der Waals (red) contributions, and the interaction energy between the azobenzene groups (green) and between the βCyD macrorings (magenta) obtained from the analysis of the 1 ns MD trajectories for the (**2**-*E*)$_2$ and (**2**-*Z*)$_2$ dimers in a 48.88^3 Å^3 periodic cubic box containing 4521 water molecules (upper and lower lane, respectively), which started on the MBE structures obtained by MM (Figure 14 in the main manuscript, -120 deg). Middle panel: Histories of the angle between the main βCyD axis and transition dipoles moments $S_0 \rightarrow S_1$ and $S_0 \rightarrow S_2$ for the corresponding azo moiety groups. Right panel: Snapshot of the structures at the end of the MD trajectory.

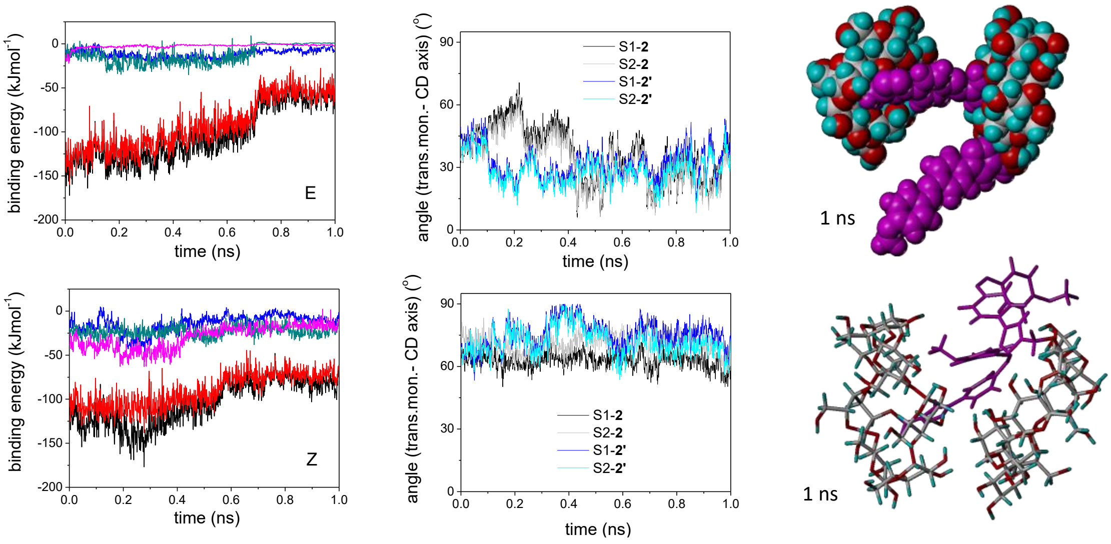

Figure S38. Left panels: Histories of the **2–2'** (total (black) interaction energies, as well as electrostatics (blue) and van der Waals (red) contributions, and the interaction energy between the azobenzene groups (green) and between the βCyD macrorings (magenta) obtained from the analysis of the 1 ns MD trajectories for the $(\mathbf{2}\text{-}E)_2$ and $(\mathbf{2}\text{-}Z)_2$ dimers in a 47.17^3 $Å^3$ periodic cubic box containing 4528 water molecules (upper and lower lane, respectively), which started on the MBE structures obtained by MM (Figure 14 in the main manuscript, 0 deg). Middle panel: Histories of the angle between the main βCyD axis and transition dipoles moments $S_0 \rightarrow S_1$ and $S_0 \rightarrow S_2$ for the corresponding azo moiety groups. Right panel: Snapshot of the structures at the end of the MD trajectory.

---

[1] J. R. Lakowicz. In *Principle of Fluorescence Spectroscopy*, 3rd ed.; Springer, 2006; ch2, p 56.

[2] J. W. Park, Fluorescence Methods for Studies of Cyclodextrin Inclusion, Complexation and Excitation Transfer in Cyclodextrin Complexes. In *Cyclodextrin Materials Photochemistry, Photophysics and Photobiology*, 1st ed.; Douhal, A., Ed.; Elsevier Science, 2006; ch1, pp 1-26.

[3] (a) Brouwer, A. M. Standards for photoluminescence quantum yield measurements in solution. *Pure Appl. Chem.* **2011**, *83*, 2213-2228. (b) Eaton, D. F. Reference materials for fluorescence measurement. *Pure Appl. Chem.* **1988**, *60*, 1107-1114.

[4] Kimball, J.; Chavez, J.; Ceresa, L.; Kitchner, E.; Nurekeyev, Z.; Doan, H.; Szabelski, M.; Borejdo, J.; Gryczynski, I.; Gryczynski, Z. On the origin and correction for inner filter effects in fluorescence Part I: primary inner filter effect - the proper approach for sample absorbance correction. *Methods Appl. Fluoresc.* **2020**, *8*, 033002.

[5] Grebenkin, S. Y.; Syutkin, V. M.; Baranov, D. S. Mutual orientation of the n→π* and π → π* transition dipole moments in azo compounds: determination by light-induced optical anisotropy. *J. Photochem. Photobiol. A.* **2017**, *344*, 1-7.

[6] Cusati, T.; Granucci, G.; Persico, M.; Spighi, G. Oscillator strength and polarization of the forbidden n→π* band of trans-azobenzene: A computational study. *J. Chem. Phys.* **2008**, *128*, 194312.

[7] Sybyl-X 2.0, Tripos International, 1699 South Hanley Rd., St. Louis, Missouri, 63144, USA .

[8] M. Clark, R. D. Cramer III, N. Van Opdenbosch. Validation of the general purpose tripos 5.2 force field. *J. Comput. Chem.* **1989**, *10*, 982-1012

[9] Frisch, M. J.; Trucks, G. W.; Schlegel, H. B.; Scuseria, G. E.; Robb, M. A.; Cheeseman, J. R.; Scalmani, G.; Barone, V.; Petersson, G. A.; Nakatsuji, H.; Li, X.; Caricato, M.; Marenich, A. V.; Bloino, J.; Janesko, B. G.; Gomperts, R.; Mennucci, B.; Hratchian, H. P.; Ortiz, J. V.; Izmaylov, A. F.; Sonnenberg, J. L.; Williams-Young, D.; Ding, F.; Lipparini, F.; Egidi, F.; Goings, J.; Peng, B.; Petrone, A.; Henderson, T.; Ranasinghe, D.; Zakrzewski, V. G.; Gao, J.; Rega, N.; Zheng, G.; Liang, W.; Hada, M.; Ehara, M.; Toyota, K.; Fukuda, R.; Hasegawa, J.; Ishida, M.; Nakajima, T.; Honda, Y.; Kitao, O.; Nakai, H.; Vreven, T.; Throssell, K.;

Montgomery, Jr., J. A.; Peralta, J. E.; Ogliaro, F.; Bearpark, M. J.; Heyd, J. J.; Brothers, E. N.; Kudin, K. N.; Staroverov, V. N.; Keith, T. A.; Kobayashi, R.; Normand, J.; Raghavachari, K.; Rendell, A. P.; Burant, J. C.; Iyengar, S. S.; Tomasi, J.; Cossi, M.; Millam, J. M.; Klene, M.; Adamo, C.; Cammi, R.; Ochterski, J. W.; Martin, R. L.; Morokuma, K.; Farkas, O.; Foresman, J. B.; Fox, D. J. Gaussian 16, revision B.01. Gaussian, Inc., Wallingford CT, **2016**.

[10] Brunel, Y.; Faucher, H.; Gagnaire, D.; Rassat, A. Program of minimization of the empirical energy of a molecule by a simple method. *Tetrahedron* **1975**, *31*, 1075-1091.

[11] Press, W. H.; Teukolski, S. A.; Vetterling, W. T.; Flannery, B. P. *Numerical recipes: the art of scientific computing*, 3rd ed.; Cambridge University Press, 2007.

[12] Blanco, M. Molecular Silverware. I. General solutions to excluded volume constrained problems. *J. Comput. Chem.* **1991**, *12*, 237-247.

[13] Tomasi, J.; Mennucci, B.; Cammi, R. Quantum mechanical continuum solvation models. *Chem. Rev.* **2005**, *105*, 2999-3093.

[14] Becke, A. D. Density-functional thermochemistry. III. The role of exact exchange. *J. Chem. Phys.* **1993**, *98*, 1372-1377.

[15] Lee, C.; Yang, W.; Parr, R. G. Development of the Colle-Salvetti correlation-energy formula into a functional of the electron density. *Phys. Rev. B* **1988**, *37*, 785-789.

[16] Yanai, T.; Tew, D. P.; Handy, N. C. A New hybrid exchange–correlation functional using the Coulomb-attenuating method (CAM-B3LYP). *Chem. Phys. Lett.* **2004**, *393*, 51-57.

[17] Fehrentz, T.; Huber, F. M. E.; Hartrampf, N.; Bruegmann, T.; Frank, J. A.; Fine, N. H. F.; Malan, D.; Danzl, J. G.; Tikhonov, D. B.; Sumser, M.; Sasse, P.; Hodson, D. J.; Zhorov, B. S.; Klöcker, N.; Trauner, ~~and~~ D. Optical control of L-type Ca2+ channels using a diltiazem photoswitch. *Nat. Chem. Biol*. **2018**, *14,* 764-767.

[18] Protasova, I.; Heißler, S.; Jung, N.; Bräse, S. Monitoring Reactions on Solid Phases with Raman Spectroscopy. *Chem. Eur. J.* **2017**, *23,* 8703-8711.

[19] Casas-Solvas, J. M.; Ortiz-Salmerón, E; Fernández, I.; García-Fuentes, L.; Santoyo-González, F.; Vargas-Berenguel, A. Ferrocene–β-Cyclodextrin Conjugates: Synthesis, Supramolecular Behavior, and Use as Electrochemical Sensors. *Chem. Eur. J.* **2009**, *15*, 8146-8162.
